# The Local Coexistence Pattern of Selfing Genotypes in *Caenorhabditis elegans* Natural Metapopulations

**DOI:** 10.1534/genetics.117.300564

**Published:** 2017-12-12

**Authors:** Aurélien Richaud, Gaotian Zhang, Daehan Lee, Junho Lee, Marie-Anne Félix

**Affiliations:** * IBENS, Département de Biologie, Ecole Normale Supérieure, CNRS, Inserm, PSL Research University, 75005 Paris, France; † Department of Biological Sciences, Seoul National University, Seoul 08826, Korea

**Keywords:** *C. elegans*, selfing, local populations, colonization, 2b-RAD

## Abstract

To study the interplay of rare outcrossing and metapopulation structure, we focus on the nematode *Caenorhabditis elegans*. Its remarkably low outcrossing rate is at the extreme end of the spectrum for facultative selfing organisms. At the demographic level, *C. elegans* natural populations undergo boom and bust dynamics on ephemeral resources, with the dauer diapause larva acting as the dispersal form. Here we investigate the small-scale genetic structure of *C. elegans* populations in two localities over several years, using 2b restriction-associated DNA sequencing of nearly 1000 individuals. We find a remarkably small number of genome-wide haplotypes, almost exclusively in the homozygous state, confirming the low effective outcrossing rate. Most strikingly, the major haplotypes in a locality remain intact and do not effectively recombine over several years. From the spatial pattern of diversity, we estimate that each subpopulation or deme is seeded by a mean of 3–10 immigrating individuals. Populations are thus formed by clones that compete at two levels, within a subpopulation and at the metapopulation level. We test for the presence of local phenotypic variation in pathogen resistance and dauer larva nictation, which could possibly explain the maintenance of different genotypes by heterogeneous selection in different local environments or lifecycles. This study is the first to address the local spatiotemporal genetic structure of *C. elegans* on feeding substrates. We conclude that these animals coexist as competing homozygous clones at the smallest population scale as well as in the metapopulation.

THE demographic and genetic structures of an organism depend on its lifecycle, life history traits, and reproductive mode. In turn, population structure affects the genetic and phenotypic evolution of the species. The small nematode *Caenorhabditis elegans*, besides being a major model organism in biology, allies interesting life history characteristics for studies of its natural populations (Cutter 2015; Frézal and Félix 2015): (1) a boom and bust metapopulation dynamics with a diapausing dispersal stage, the dauer larva; and (2) reproduction by selfing of hermaphrodites or rare outcrossing between hermaphrodites and males, at the very low end of the outcrossing frequency spectrum for facultative outcrossing organisms (Jarne and Auld 2006). Here we address the spatiotemporal genetic structure and the presence of ecologically relevant phenotypic polymorphisms in local natural populations of *C. elegans*.

The first genetic surveys of natural *C. elegans* populations focused on compost heap populations (Barrière and Félix 2005, 2007; Haber *et al.* 2005; Sivasundar and Hey 2005). Compost heaps are largely dominated by human intervention; moreover, heat during the composting process dooms the *C. elegans* populations therein. Therefore, we next sought more natural and lasting habitats. *C. elegans* was found feeding in rotting fruits and rotting stems and carried by invertebrates (Kiontke *et al.* 2011; Félix and Duveau 2012; Petersen *et al.* 2015). In a previous article, we reported on the demography of such *C. elegans* populations, focusing on two locations in France, an apple orchard in Orsay and a wood in Santeuil (Félix and Duveau 2012). These demographic data were consistent with a metapopulation structure: an exponential growth phase via the feeding larval and adult stages, followed by entry of the young larvae into the dauer diapause stage and dispersal to a new food source (Cutter 2015; Frézal and Félix 2015). The dauer larvae disperse using their own locomotion or attaching to larger animals such as isopods.

At the genetic level, two spatial scales have been considered so far: the worldwide level and the local level, focusing on the then available populations from compost heaps. At the worldwide level, *C. elegans* isolates (which were previously isogenized by selfing) were genotyped by SNP genotyping (Rockman and Kruglyak 2009), restriction-associated DNA (RAD) sequencing, or whole-genome sequencing (Andersen *et al.* 2012; Cook *et al.* 2016). These studies demonstrated chromosome-wide linkage disequilibrium in the species. When combined with selfing, migration, and positive selection, a low recombination rate may result in loss of diversity. In particular, the weakly recombining centers of several chromosomes (I, IV, and V) show little polymorphism as a likely consequence of recent worldwide selective sweeps (Andersen *et al.* 2012). At the global scale, the species displays a weak geographic structure between the European and North American continents (Andersen *et al.* 2012), and further diversity has recently been found in the Pacific area (Cook *et al.* 2016).

At the local scale, compost heap populations were analyzed using microsatellite or amplified fragment length polymorphism genotyping, or sequencing of a few loci (Barrière and Félix 2005; Haber *et al.* 2005; Sivasundar and Hey 2005; Cutter 2006). These studies showed that compost heaps often contain several *C. elegans* multilocus haplotypes, with a relatively low percentage of heterozygotes and an even lower occurrence of recombinants. However, these studies had limitations: the artificial nature of compost heap formation and the fact that mostly dauer larvae were found. The compost heap populations are likely mostly population sinks for dauer larvae. These features make it difficult to assess their evolutionary relevance.

Here, we report for the first time the genotyping of local natural populations of *C. elegans* in rotting fruits and stems, populations that may contribute to the future of the species. Our questions were the following: How do life history characteristics shape the demographic and genetic structures of the organism? Can distinct genotypes be found competing within a single booming, reproductively active subpopulation (a deme)? If so, what is the average number of colonizing individuals at each boom cycle? Are heterozygote and recombination frequencies indeed low? Given that *C. elegans* could not be found easily in the orchard from winter through to summer, were genotypes of the previous year reseeding the populations in the fall? And finally, are local genetic polymorphisms reflected in variation in ecologically relevant traits that could explain the observed long-term maintenance of distinct genotypes?

A small part of the local populations we collected in Félix and Duveau (2012) were analyzed using microsatellites and genome hybridization methods by Volkers *et al.* (2013), who concluded to the presence of a significant structure between two locations but did not investigate intralocation spatial and temporal structure. To assay multilocus genotypes with an unbiased genotyping method, we chose the 2b-RAD sequencing approach (Wang *et al.* 2012), where a type IIb restriction enzyme is used to generate short DNA fragments that can be directly ligated to sequencing adapters. For high-throughput sequencing of these fragments, we used a combinatorial multiplexing strategy and genotyped ∼1000 individuals.

We complemented the genetic analysis by an analysis of ecologically relevant phenotypic variation in the Orsay location. In particular, we wondered whether selection acting during the different stages of the lifecycle (exponential growth and dauer migration stage) could explain the maintenance of genetic diversity. We thus introduce the known ecologically relevant phenotypic variation in *C. elegans* acting either during the exponential growth phase or during the dauer stage. For some of these phenotypes, underlying molecular variants have been found, which we could directly genotype.

First, during the exponential growth phase of populations, pathogens represent an important source of selection. The microsporidia *Nematocida parisii* and *N. ausubeli* are the most common intracellular pathogens found in wild *C. elegans*, including in the locations we study (Troemel *et al.* 2008; Zhang *et al.* 2016; Schulenburg and Félix 2017). Compared to healthy *C. elegans*, infected *C. elegans* display a paler color appearance, a thinner and smaller body size, a large reduction in fecundity and longevity (Troemel *et al.* 2008; Balla *et al.* 2015, 2016; Zhang *et al.* 2016). Linkage mapping between the N2 reference strain and the distant Hawaiian CB4856 isolate uncovered resistance loci, but they were not so far narrowed down to the gene level (Balla *et al.* 2015). Another pathogen slowing down the exponential growth of *C. elegans* population is a RNA virus that was discovered in the Orsay location studied here (Félix *et al.* 2011). Infection by the Orsay virus of the Orsay JU1580 strain slows down progeny production (Ashe *et al.* 2013). Genome-wide association mapping showed that a large part of the phenotypic variation in *C. elegans*’ viral load after experimental infection was explained by an indel polymorphism in the *drh-1/RIG-I* gene (Ashe *et al.* 2013), which we could genotype in our local *C. elegans* collection.

Second, at the metapopulation level, the dauer diapause stage matters for migration to a new food source and long-term maintenance (Frézal and Félix 2015). *C. elegans* dauer larvae are known to nictate, a behavior whereby they stand on their tail either individually or in groups, and wave. This behavior increases their chance of being carried away by larger animals (Lee *et al.* 2011), which in turn facilitates their colonization of a new, nutrient-rich habitat. The frequency of occurrence of this behavior has been recently shown to be polymorphic in the species (Lee *et al.* 2017). We assayed it as a proxy for its ability to be carried away by a vector.

Finally, concerning reproduction by outcrossing, *C. elegans* has been shown to be subject to outbreeding depression (Dolgin *et al.* 2007). One well-studied molecular polymorphism resulting in decreased fitness of outcrossed *C. elegans* progeny is the *peel-1zeel-1* indel polymorphism. These loci correspond to two tightly linked genes coding for a toxin–antitoxin system with paternal and zygotic effects, respectively (Seidel *et al.* 2008, 2011). Embryos that do not bear the *zeel-1* antitoxin gene but receive the sperm PEEL-1 toxin will die. The spread of the insertion is thus favored in a cross with an isolate not bearing it. We wondered whether this indel polymorphism was present locally.

Overall, we found a limited number of homozygous diploid genotypes (treated as haplotypes) over several years in each location. The haplotype frequency spectrum strongly differs between the two main sampling sites. In the Orsay apple orchard, one homozygous genotype strongly dominates over a 7-year span, cohabiting with a dozen minor haplotypes. The Santeuil wood harbors only three haplotypes, all at intermediate frequencies. At the scale of a few meters, when two genotypes are present, a given sample (fruit, stem) is likely to harbor both. Simulations suggest that each deme is seeded by 3–10 individuals on average. The heterozygote frequency as well as the effective recombination rate are extremely low, supporting previous findings. Finally, we detect phenotypic polymorphisms in pathogen resistance and dauer nictation that may be maintained locally by heterogeneous selection in varying environments.

## Materials and Methods

### *C. elegans* collection

Samples potentially containing *C. elegans* were collected from three locations in France: Orsay, Santeuil, and Plougasnou (Félix and Duveau 2012) (Supplemental Material, Figure S1). In Orsay (GPS coordinates 48,702, 2172), rotten apples were collected in an orchard between October 2008 and October 2014. In Santeuil (49,121, 1951), rotten stems, snails, and slugs were collected in a wood along a small stream between October 2009 and October 2014. In Plougasnou, rotten stems were collected in a wet shrubland in August 2009 (48,705, −3795). Each sample was called “O” (for Orsay), “S” (for Santeuil), or “P” (for Plougasnou), followed by an identifying number. Population census sizes and developmental stage compositions are reported in Félix and Duveau (2012), except for later timepoints of Orsay and Santeuil, which are reported in Table S2.

Freshly collected samples were placed around an OP50 *Escherichia coli* lawn on NGM 90 mm plates (Stiernagle 2006). A single *C. elegans* hermaphrodite individual was isolated on a NGM 55 mm plate (1–12 individuals per sample). We thus isolated 10–12 wild-born individuals in samples that contained a sufficiently large population. A single individual was kept for samples where *C. elegans* was only found after 2–3 days. The animals were let to reproduce for two generations before freezing the progeny of a single wild-born individual (Barrière and Félix 2005, 2014). Populations derived from a single wild individual were maintained without bottleneck to maintain heterozygosity (if present). The wild-sampled individuals have names such as O749.7 for the third individual of Orsay apple O749.

In some cases, especially for phenotypic analysis, isogenic lines were derived by isolating a single selfed hermaphrodite for a few generations and given a laboratory strain name such as JU2815. Individuals of the first timepoint in Orsay were selfed and singled for a few generations to produce stable isogenic strains (Table S3).

### Sampling structure

The Orsay location (30 km south of Paris, France) corresponds to an apple orchard, which was sampled at 26 timepoints from 2008 until 2014, 17 of which were positive for *C. elegans*. The Orsay samples are rotting apples, as well as some associated invertebrates such as slugs. At each timepoint, 20–26 apples were sampled throughout the orchard, a square area of ∼100 × 100 m. In addition, on two occasions in 2008, a more local sampling consisted in four groups of five apples below a single tree (Félix and Duveau 2012); the distance within a group was in the range of 1–20 cm, the distance between groups was 0.5–2.5 m, and each apple was cut into pieces in the field.

The second location was a wood in Santeuil (60 km northwest of Paris, France), sampled from 2009 to 2014 generally once or twice per year. The Santeuil samples are mostly rotting stems of herbaceous plants, such as the *Heracleum* or *Symphytum* species, as well as associated isopods, snails, and slugs. The samples were collected along a stream, in a thin band of ∼200 m in length.

The third location was a wet coastal shrubland in Plougasnou (Brittany in western France), sampled once in 2009. The Plougasnou samples are rotting stems, leaves, and sloe fruits (Table S1). They were added to the analysis to provide another example of a relatively natural setting to assess local diversity at different scales.

Details on the population census, developmental stage, and occurrence of males can be found in Félix and Duveau (2012) and Table S2 for the most recent sampling dates.

### Genomic DNA extraction

We analyzed the genotype of each individual by preparing DNA of its pooled progeny, avoiding any bottleneck, and kept part of the population frozen for further analyses. The genotype of the pool should thus represent the genotype of the sampled individual with, if any, heterozygous loci showing two alleles (although possibly deviating from a 50% frequency through some drift or selection in the laboratory).

After thawing, each strain was bleached and grown on 90 mm NGM plates enriched with agarose (for 1 liter: 3 g NaCl, 5 g bacto-peptone, 10 g agar, 7 g agarose, 1 ml cholesterol 5 mg/ml, 1 ml CaCl_2_ 1 M, 1 ml MgSO_4_ 1 M, 25 ml KPO_4_ 1 M). Worms were harvested just after starvation and washed in M9 (Stiernagle 2006) several times to remove *E. coli*. The worm pellets were resuspended in 600 µl of Cell Lysis Solution (Qiagen, Valencia, CA) complemented with 5 µl of proteinase K (20 µg/µl) and incubated overnight at 56° with shaking. The lysates were then incubated for 1 hr at 37° with 10 µl of RNAse A (20 µg/µl) and the proteins were precipitated with 200 µl of protein precipitation solution (Qiagen). After centrifugation, the supernatants were collected in new tubes. Genomic DNA was precipitated with 600 µl of isopropanol. The pellets were washed in ethanol 70% and dried for 1 hr before being resuspended in 50 µl of DNAse-free water.

### 2b-RAD library preparation

A multiplexing strategy with eight barcodes and 24 Illumina indices was adapted from the 2b-RAD method developed by Wang *et al.* (2012), to prepare libraries of 192 pooled samples (Figure S2). Oligonucleotide sequences are shown in Table S1.

First, 1 µg of genomic DNA of each sample was digested overnight at 37° in a final volume of 6 µl with the type IIB restriction endonuclease *Bcg*I (NEB). The *Bcg*I enzyme cuts outside its recognition site and creates a 32 bp fragment with 2-base 3′-overhangs (36 bp total). Selective double-stranded adaptors with eight different sample-specific barcodes (Table S1) were then ligated with T4 ligase to the 36 bp fragments, for 1 hr at 16° in a final volume of 20 µl. The constructs produced by ligation were amplified with Phusion Taq polymerase (NEB) with 23 PCR cycles of 5 sec at 98°, 20 sec at 60°, and 10 sec at 72°, using a set of 24 primers that introduce a second sample-specific barcode (standard Illumina index) on the other end. The fragments with distinct 5′ and 3′ adapters (red and blue in Figure S2) are favored in the PCR because self-annealing strands do not amplify well. The number of PCR cycles was chosen to be minimal and still produce a visible band on a gel.

The 192 PCR fragments were then pooled and loaded onto a 2% agarose gel. The target band (160 bp) was cut out and purified on columns with a gel extraction kit (Nucleospin Macherey Nagel). A second round of gel purification was performed in order to further eliminate primer dimers. The PCR product was eluted in 30 µl of DNAse free water and dosed with Qbit (Thermo Scientific).

Seven libraries of 192 samples were constructed on the same model and sequenced on a HiSequation (51 bp single read using a TruSeq SBS Kit v3; Illumina), by the high-throughput sequencing platform of I2BC (Gif-sur-Yvette, France), adding phage DNA to increase sequence heterogeneity.

### 2b-RAD sequence analysis

After adapter removal, the remaining 40 bp sequences were filtered according to the presence of the restriction site and of the primer W nucleotide, allowing for unclear sequence (N) at these sites. The reads were then processed using process_radtags in *Stacks 1.30* (Catchen *et al.* 2011), allowing for a maximum of one mismatch in the four-nucleotide barcode. The sequences were trimmed to 36 bp and parsed into individual fastq files bearing the sample name, then mapped to the *C. elegans* WS248 genome (downloaded from www.Wormbase.org) using *bowtie* (Langmead *et al.* 2009), allowing for two mismatches (polymorphisms). This number of mismatches was chosen based on the low level of sequence polymorphism in the species (average nucleotide diversity at neutral sites of ∼10^−3^/bp; Barrière and Félix 2005; Cutter 2006; Rockman and Kruglyak 2009; Andersen *et al.* 2012). Most unmapped reads corresponded to *E. coli* sequences, which were in variable quantity depending on the sample (most in the 2–10% range and in a few cases >50%). The mapped sequences were analyzed using *Stacks 1.30* with the ref_map routine (with parameter *n* = 1, else default parameters) and a *mysql* database.

All individuals could not be analyzed at the same time because of memory limitations, so groups of individuals were first analyzed with the *Stacks populations* script per location, and in Orsay per years, and the results exported as a vcf file. *vcftoools* was then used to filter out genomic sites with data for <70% of the individuals in the population and with mostly “heterozygous” as called variants (mismapping/duplications/PCR errors). Each individual was assigned to a haplotype or labeled as a putative heterozygote. All individuals of a given main haplotype were reanalyzed as a group for polymorphisms. 2b-RAD sequencing produces sequences on opposite strands derived from fragments ligated in opposite directions. PCR errors may appear in reads in one direction, but not the other. We used the fact that *Stacks* returns the +1 position on the minus strand (of the reference genome) to verify genotypes on both strands in the vcf file, and then eliminate the minus strand entries. The web display of the *Stacks mysql* database was used to filter dubious sites. Representatives of all haplotypes of all locations were finally analyzed as a group.

We used 7760 fragments of 36 bp in the *C. elegans* reference genome (distributed along each chromosome at an average of every 13 kb). We did not utilize polymorphisms of presence versus absence of the fragments nor small insertions or deletions. Thus, we used ∼30 bp of information for each fragment. Given the low level of polymorphisms (nucleotide diversity ∼10^−3^/bp) in *C. elegans*, we could expect an average of 30 × 7760 × 10^−3^ = 233 polymorphic sites between two random worldwide isolates, likely higher than average local diversity.

When the same RAD locus (36 bp) contained two SNPs that were fully in phase in our dataset (17 occurrences), only the first SNP was retained. If the SNPs were not in phase, both were retained. After stringent filtering, we kept 275 informative sites. Based on these 275 loci, the mean divergence among haplotypes is 4.44 × 10^−4^ substitutions per nucleotide in Orsay and 3.22 × 10^−4^ in Santeuil. Because of the stringent filtering and the collapsing of fully linked variants in the same RAD locus (Table S3), these numbers underestimate absolute nucleotide diversity at the whole genome level.

The subset of strains analyzed by Volkers *et al.* (2013) did not cover all main haplotypes in our study, for example, not the Santeuil HS2 genotype. Moreover, their groups of haplotypes in the microarray hybridization and microsatellite data do not correspond to ours. Thus, we cannot provide a comparison and prefer to use a different genotype nomenclature, numbering each haplotype in a location as HOn for a Orsay haplotype (*e.g.*, HO1), HSn in Santeuil, and HPn in Plougasnou.

### Experimental validation of genotypes

The data were further tested experimentally by genotyping SNPs using pyrosequencing (Table S4). Individuals that were putative heterozygotes at many sites were rescreened by genotyping one or a few SNPs. Apart from the three confirmed cases that were heterozygous at nearly all polymorphic sites between two main haplotypes, we did not confirm any other putative heterozygous loci. The partial heterozygosity likely corresponds to low quality and partial PCR contamination in the RAD sequencing test.

Several cases of multiple genotypes per sample were also checked, focusing on the most dubious and unexpected cases. All RAD results of mixing of genotypes within a sample were confirmed, except for the inversion of S135.3 and S151.1 in the RAD data compared to the original frozen tube.

### Population genetic analyses

STRUCTURE and principal component analysis were performed using *SNiPlay* (http://sniplay.southgreen.fr/cgi-bin/home.cgi) (Dereeper *et al.* 2011). Haplotype networks were built using *SplitsTree4* (Huson and Bryant 2006). Comparison with a worldwide dataset was made using April 2016 data on hundreds of 152 worldwide haplotypes (Cook *et al.* 2016) downloaded from https://elegansvariation.org. Hierarchical *Fst* estimations were performed using the *hierfstat* package in R (Goudet and Jombart 2015). To plot the spatial structure in Santeuil in Figure 3, a single spatial coordinate was estimated by projection of the sample position onto the main axis of the sampling (approximately north-south axis, Figure S7). The origin (0 m) is at the north end.

### Number of colonizing individuals

We selected all samples that corresponded to cases where two genotypes were present at distances of <1 m (excluding leaf litter, soil, rotting grass, moss, and snail samples). We found that 60% (*n* = 10) of the stem or fruit samples carried these two haplotypes when at least six individuals were genotyped, and 50% when at least two individuals were genotyped (*n* = 18) (Figure 5). As the number of genotyped individuals is low in some instances (*e.g.*, two), these proportions are underestimates of the true proportion. We used R version 3.2.1 (R Core Team 2015) to estimate the maximum likelihood of the proportion of samples with more than one genotype if the data were not limited by the number of genotyped individuals. Using the 18 samples, we assumed a given frequency of the two alleles in each sample drawn from a random uniform distribution, and computed the probability of the observed data on the samples with a single genotype if they in fact had contained two genotypes. The maximum likelihood estimate is 61.7 ± 0.4% (*n* = 100), matching well the observed data (60%) for samples with six or more individuals. We used this number for the estimation of the number of colonizing individuals below.

Using R and a Poisson distribution of coefficient λ, we fitted the number of colonizing individuals that could account for this proportion of 62% samples with more than one genotype. The local genotype frequency *f* that formed the source of colonizing individuals was modeled in several manners: fixed to 0.5 or 0.1 for the plots in Figure 5; a random uniform distribution; or a symmetric β-distribution, as the detection of two haplotypes locally implied that the minor allele frequency was above a certain threshold. The probability of obtaining a single genotype when colonization occurred was calculated as the sum of the probabilities for either genotype given the allele frequency *f*, summing over the probability of a given number *i* of immigrating individuals given the Poisson distribution of mean λ: $p=\;\sum_{i=1}^{200}\lambda^{i}e^{-\lambda }(f^{i}+{\left(1-f\right)}^{i})/(i!\left(1-e^{-\lambda }\right)).$ We used the R formula: for (*i* in 1:200) {*P* = *p* + dpois(*i*, l) × (*f*^*i* + (1−*f*)^*i*)/(1−dpois(0, l))}. In the unrealistic case of equal proportions of the two genotypes, we obtain a mean of 2.9 immigrating individuals (an underestimate). If we assume a minor allele frequency of 0.1, the coefficient of the Poisson distribution rises to 10 individuals. If we simulate a random uniform allele frequency distribution, the mean of the Poisson coefficient over the replicate trials is of 11 individuals and the median is of 4 (*n* = 10,000). If we simulate with a β-distribution with parameters (2, 2), we obtain a Poisson coefficient mean of 4.8 immigrating individuals and a median of 4 (*n* = 10,000).

### Genotyping of specific genes

The *drh-1* deletion polymorphism was found by association mapping to be the main locus explaining variation among *C. elegans* isolates in Orsay virus replication level after laboratory infection. The *drh-1* polymorphism was genotyped by PCR using the primers oTB40 and oTB43 as described by Ashe *et al.* (2013). The *zeel-1peel-1* deletion polymorphism was previously found to be involved in an incompatibility between isolates and embryonic lethality in some crosses and was genotyped with a three-primer PCR as described by Seidel *et al.* (2008). These primers and their sequences are listed in Table S1, sheet 3.

### Dauer induction and nictation assays

We assayed nictation of dauer larvae as a proxy for their ability to migrate. Nictation is a specific dauer larva behavior, where they stand on their tail and wave their body, helping them being carried away by larger invertebrates such as isopods (Lee *et al.* 2017). To induce dauer larvae, 10–20 L4 larvae or young adults were transferred to *E. coli* OP50-seeded synthetic pheromone plates containing agar (10 g/liter), agarose (7 g/liter), NaCl (2 g/liter), KH_2_PO_4_ (4 g/liter), K_2_HPO_4_ (0.5 g/liter), cholesterol (8 mg/liter), and ascaroside 1, 2, and 3 (0.5 mg/liter each), at 25° for dauer induction. Four days after the transfer, microdirt chips were made by pouring 3.5% agar solution onto a PDMS mold (Lee *et al.* 2011). The solidified agar microdirt chip was detached from the PDMS mold and dried for 90 min at 37°. Dauers in synthetic pheromone plates were identified morphologically (dark intestines and radially constricted bodies). More than 30 dauers were collected from the synthetic pheromone plates in M9 buffer using a glass capillary (Chase) and placed on a microdirt chip. After 10–30 min, when most dauers move actively, the fraction of nictating dauers among moving dauers was measured. The nictation ratio was calculated from the independent biological replicates of multiple nictation assays. Quiescent dauers were not scored.

We tested representatives of 11 Orsay genotypes (Figure 6). All strains were tested on eight different assay blocks (days) (one assay of JU1581 and JU1511 failed). An ANOVA analysis was conducted in R on arcsine-transformed data using strains nested in haplotype and day of assay: nic = haplotype(strain) + day. The effect of haplotype and day were highly significant (*P* = 1.11 10^−14^ and 2.04 10^−9^, respectively). The strains were grouped in a *post-hoc* test with a Tukey honest significant difference test at *P* = 0.05, using R package *agricolae* (de Mendiburu 2016). The same test was performed on the viral load data for the Orsay orchard strains in Ashe *et al.* (2013).

### Competition assays

We tested whether the presence of a microsporidian pathogen could affect the outcome of pairwise competitions between *C. elegans* isolates. Twelve competition assays involving six different pairs of strains corresponding to the Orsay genotype HO1 and another genotype (HO2 or HO8) were performed at 23°, as previously described by Duveau and Félix (2012), in the presence or absence of microsporidia infection. For each competition assay, five replicate populations were founded from 10 L4 larvae of each strain deposited onto 90 mm diameter NGM plates seeded with *E. coli* OP50. For competition assays with microsporidia infection, the *N. ausubeli* strain JUm2009 from Orsay was used for inoculation. Spore preparations of JUm2009 were extracted and quantified as previously described (Zhang *et al.* 2016). Five million microsporidian spores in 150 μl distilled water were placed on the *E. coli* lawn. The infection symptoms were checked by Nomarski optics at 48 hr after inoculation. The populations were grown at 23°. Just before starvation, worms were harvested in M9 and centrifuged 5 min at 3000 rpm. A total of 2 µl of pellet was then placed onto a fresh culture. Each uninfected population was thus transferred every 2 days. Each infected population was transferred every 4 or 5 days. The proportion of each strain was quantified at the different timepoints by pyrosequencing on a PyroMark Q96 ID instrument (Biotage/Qiagen), using an SNP identified on the chromosome V between HO1 and the two other haplotypes, HO2 and HO8 (RAD4-chV, T/G at position V: 2,639,853; see Table S1). The allele frequencies were quantified from the height of the pyrogram peaks using the Allele Quantification tool of the PyroMark ID software (Biotage/Qiagen).

### Data availability

Strains are available on request. The fastq 2b-RAD file for each individual is available through NCBI short read archive (accession number PRJNA399509). Table S1 lists primers. Table S3 contains genotypes for each individual.

## Results

### Allelic diversity but low haplotypic diversity in each location

Our sampling substructure in three locations can be found in the *Materials and Methods*. We genotyped each of 956 wild individuals using a 2b-RAD sequencing strategy. Our filtered dataset includes 275 SNP sites covering the six chromosomes (Table 1 and Table S3), a number consistent with the known level of polymorphism in *C. elegans* (see *Materials and Methods*). Over 99% of the individuals were homozygotes and are thus characterized by a haplotype (multilocus genotype). Genotypes for each individual can be found in Table S5, Table S6, and Table S7.

**Table 1:** Sample structure in the three locations over space and time

Location	Orsay	Santeuil	Plougasnou	Total
Landscape	Orchard	Wood, along a stream	Wet coastal shrubland	
Sampled area	Square 100 × 100 m	Band 100 × 10 m	Band 200 × 10 m	
Substrates	Apples, animal vectors	Stems, animal vectors	Stems	
Years	2008, 2009, 2010,	2009, 2010,	2009	
	2011, 2013, 2014	2011, 2013, 2014		
Timepoints	24 + 2	7	1	
Sampling scheme 1	24 Timepoints ×	7 Timepoints ×	1 Timepoint	
	20–26 apples across the orchard	10–40 samples along the line		
Sampling scheme 2	2 Timepoints ×	—	—	
	four groups of five subsampled apples			
Positive timepoints	15 + 2	7	1	
Positive samples	83 + 17 Apples	86	7	
Genotyped individuals	442 + 132	340	42	956
Main haplotypes	13	3	5	19
Confirmed heterozygotes	—	3	—	
Polymorphic loci	209	75	92	275
Mean nucleotide divergence among main haplotypes	4.44 × 10^−4^			
Shared with another of the three locations	108 (52%)	75 (100%)	76 (83%)	158 (57%)
Shared with the world	200 (96%)	75 (100%)	87 (95%)	261 (95%)
Singleton within-haplotype group	2	0 (1 Recombination event)	2	4

In each location, we found a strong structure of genetic diversity, where all individuals could be placed in a few homozygous genotype groups (haplotypes), revealing a high level of selfing and linkage disequilibrium. The 574 individuals in Orsay only corresponded to 13 homozygous multilocus genotypes, which we called HO1–HO13 (HO for haplotype Orsay). The 340 individuals in Santeuil corresponded to only three main haplotypes, which we called HS1–HS3, and three heterozygotes between these haplotypes. Finally, the 42 individuals in Plougasnou corresponded to five main haplotypes (HP1–HP5) (Table 1). We detected a few minor variants of these main haplotypes. For example, HO1-1 and HO1-2 are distant by one SNP from HO1, and similarly for HP1-1 and HP5-1. HS2-1 has the right tip of chromosome V from HS3, but is otherwise identical to HS2 (Table S3).

Two of the 19 haplotypes were shared between the three locations: HO2 was found in Plougasnou (called HP5) and HO7 was found in Santeuil (called HS2). In addition, most polymorphisms were not specific to a location. A total of 95% of the SNPs were present in a worldwide dataset (Cook *et al.* 2016) (https://elegansvariation.org/) and a majority (57%) were shared between at least two of the three locations (Table 1). Consistent with previous studies (Barrière and Félix 2005; Andersen *et al.* 2012), most of these alleles are likely identical by descent. Of the remaining 14 new polymorphisms, four were singletons separating minor variant haplotypes (such as HO1-1) that are likely to represent new local mutations. Thus, overall, most polymorphisms did not arise locally but corresponded to immigrating alleles.

**Figure 1:**
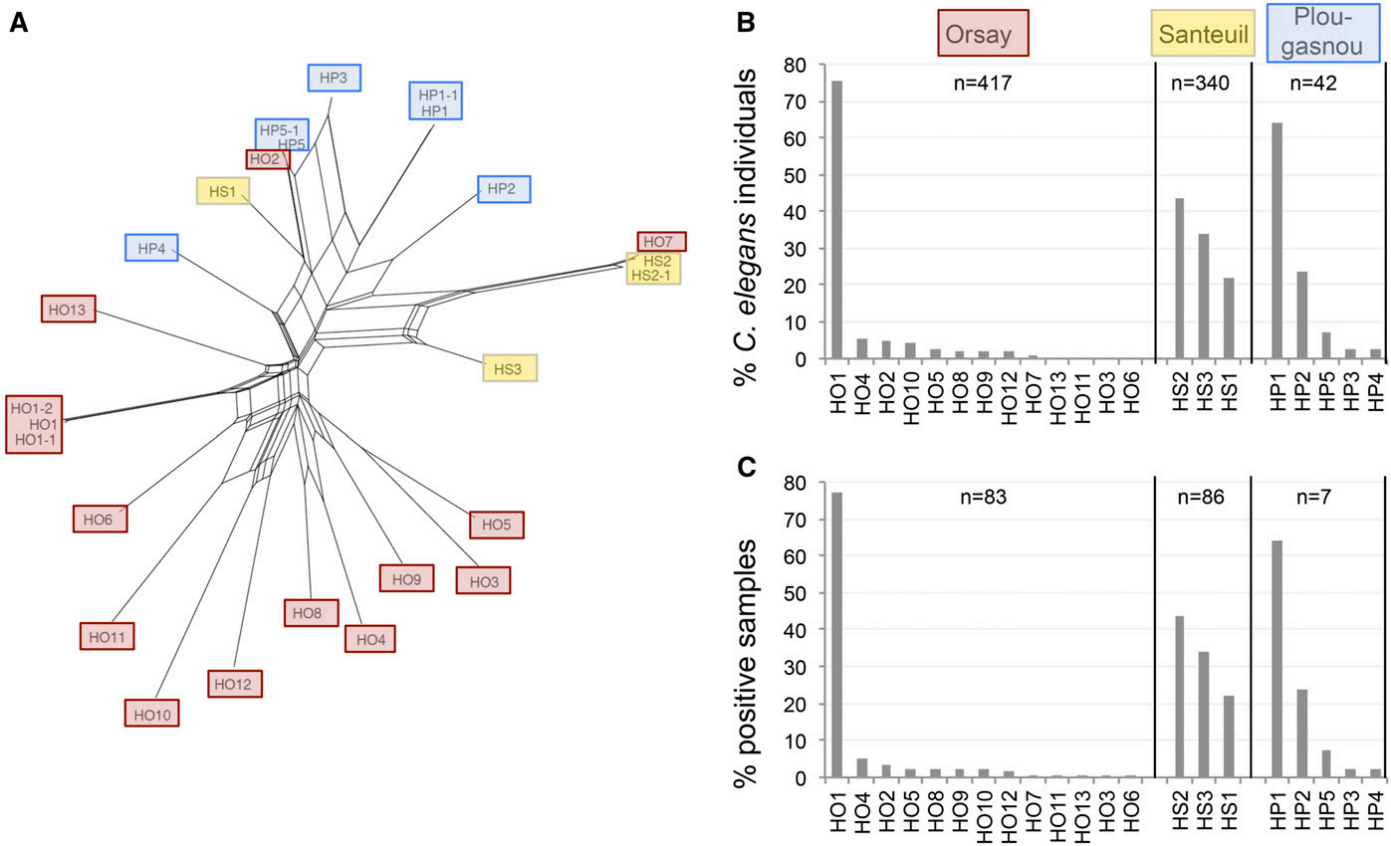
Only a few *C. elegans* haplotypes are present in each location. (A) Haplotype network over the whole dataset. Only few different genotypes were found among the 956 genotyped individuals, forming 20 haplotype groups. The Orsay haplotypes are boxed in red, those from Santeuil are in yellow, and those from Plougasnou in are blue. As an example, the distance between HO1 and HO6 is 52 SNPs of our dataset. (B) Haplotype frequency spectrum in each location, expressed as a proportion of all genotyped individuals. (C) Haplotype frequency spectrum in each location, expressed as a proportion of *C. elegans*-positive samples. Note that many samples carry two genotypes, so the proportions add up to more than one. The number of individuals and samples for each location are indicated above each respective graph. In Orsay, only individuals from sampling scheme 1 were used in (B) and (C). Note that sampling scheme 2 did not yield new haplotypes.

Figure 1A displays the relationship among the 19 main haplotype groups as a haplotype network (see Figure S3 for principal component and STRUCTURE, analyses and Figure S4 for haplotype networks per chromosome). Despite the pervasive origin of alleles by immigration, the different haplotypes tended to cluster by location, with exceptions such as HO2 and HO7.

As observed before (Barrière and Félix 2005, 2007; Haber *et al.* 2005; Sivasundar and Hey 2005), the genetic differentiation between locations (distances >10 km) was high, with *Fst* = 0.52 between the three locations. New in this dataset is the large number of subpopulations sampled in each location. From a hierarchical *Fst* analysis, we found that the differentiation of samples within locations was even higher, with *Fst* = 0.78 over the whole dataset. This high local differentiation was expected from the high rate of inbreeding coupled with bottlenecks in seeding sample-level subpopulations (Barrière and Félix 2005). We show below that the level of differentiation depends on the very local availability of genotypes within one to a few meters.

### A predominant haplotype in Orsay coexists with minor haplotypes over several years

**Figure 2:**
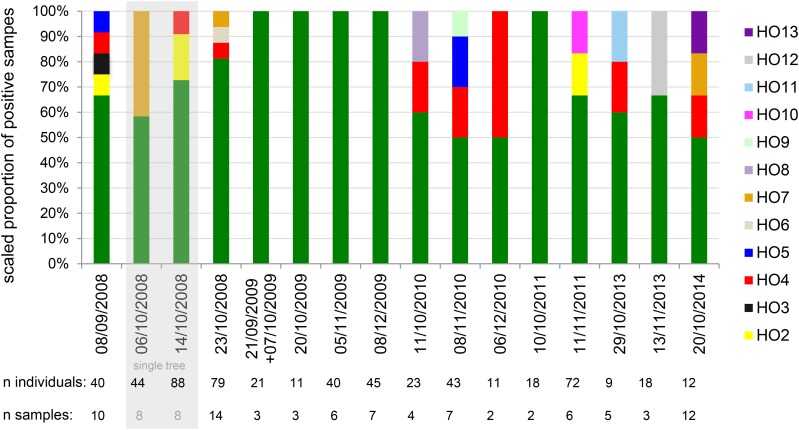
High frequency of one major genotype and stable occurrence of some minor haplotypes in Orsay. Dates on the *x* axis are expressed as DD/MM/YYYY. The % in the *y* axis corresponds to a collapse to 100% of the data expressed as proportion of *C. elegans*-positive samples with this haplotype as in Figure 1. The haplotypes are color coded. The two timepoints corresponding to sampling scheme 2 (sampling below a single tree) are labeled “single tree” and boxed in light gray. The sampled tree is different for the two timepoints. The numbers of positive samples at each timepoint are indicated above the graph. For individual data, see Table S5.

In Orsay, one haplotype (HO1) vastly predominated, covering 77% of the individuals and 78% of the *C. elegans*-positive samples (excluding 2008 collections with a different sample structure). This major haplotype remained predominant over the 7-year span of the survey (Figure 1, B and C and Figure 2).

Besides HO1, 12 minor haplotypes could be found. Of these, HO4 was the most abundant (5.4% of individuals, 10.8% of samples) and was found on 4 of the 6 years. HO2, HO5, and HO7 were found in 2008 and one other year, whereas HO3, HO6, and HO8–HO13 were found at a single timepoint (Figure 2 and Table S5). The absence of minor haplotypes in our dataset in a given year may only represent insufficient sampling given their low frequency. However, the relative proportion of the haplotypes differed significantly among years (Pearson’s chi-squared test with simulated *P*-value based on 10^6^ replicates: *P* = 0.0068). Notably, HO1 was the only haplotype found in 2009 (117 individuals from 19 samples).

The spatial distribution of genotypes (Figure S5) was dominated by HO1 and minor genotypes were too rare to form a significant pattern. The second most frequent haplotype, HO4, appeared distributed over the orchard in 2008 but suggestively only appeared in the west part in three subsequent years (seven samples).

### Stable occurrence and spatial shifts of haplotypes over several years in Santeuil

**Figure 3:**
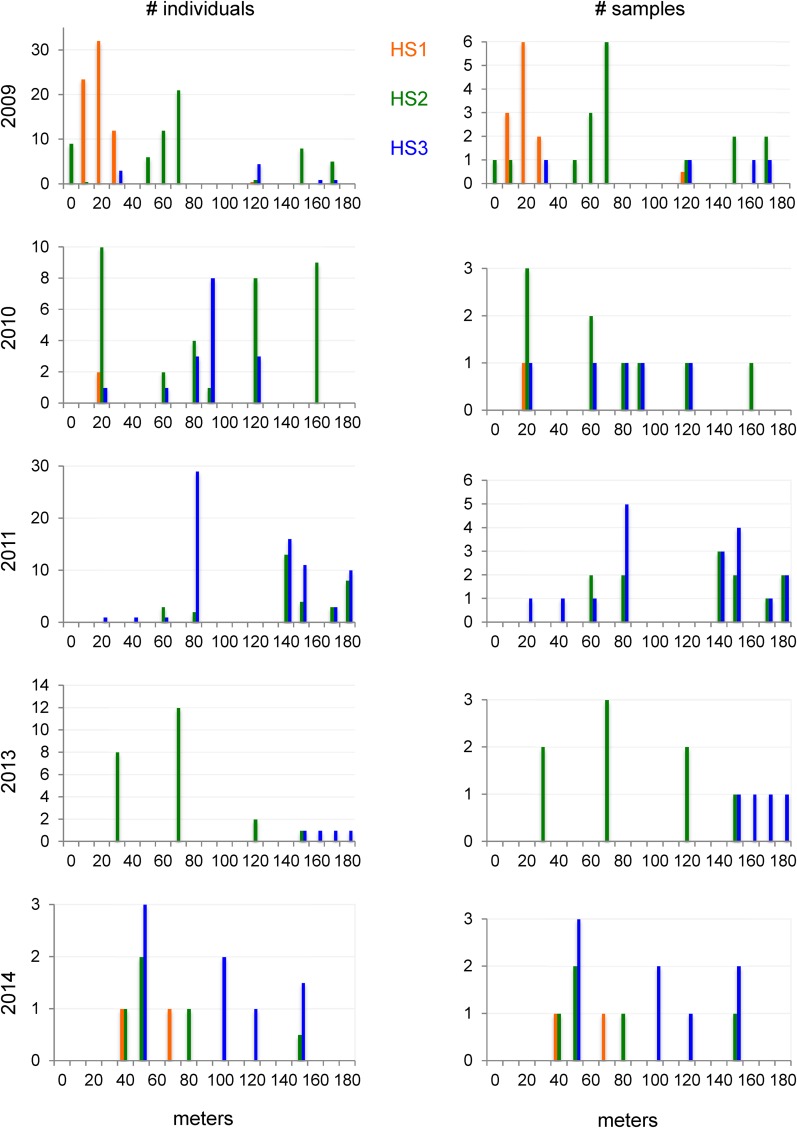
Spatiotemporal structure of major haplotypes in Santeuil. Each graph represents the distribution in space of the three local haplotypes (color coded) for a given year, on the left as a proportion of all genotyped individuals, on the right as a proportion of *C. elegans*-positive samples. The *x* axis represents position along the sampling transect, binned by 10-m intervals.

The Santeuil site displayed a strikingly low haplotypic diversity, with only three main haplotypes. However, unlike the haplotypic distribution in Orsay, the three Santeuil haplotypes were present at similar intermediate frequencies (21, 46, and 33% of the individuals and 17, 58, and 44% of the samples with HS1, HS2, and HS3, respectively) (Figure 1, B and C). HS2 and HS3 were found every year, whereas HS1 was found in 2009, 2010, and 2014 (Figure 3). Their proportion differed significantly among years (Pearson’s chi-squared test with simulated *P*-value based on 10^5^ replicates: *P* = 10^−4^).

The spatial signal was strong in the first year of sampling (2009), with HS1, HS2, and HS3 occupying successive positions along the north-south transect, with mixing of HS2 and HS3 in the southmost part (Figure 3 and Figure S7). The northern most part with haplotype HS1 corresponds to a patch containing the plant *Tussilago* sp. (Table S6), which is found nowhere else. This spatial signal faded in 2010 and 2011, when HS2 and HS3 coexisted all along the transect, including in the area where HS1 was found in 2009 (Figure S7 and Table S6). The 2009 spatial structure appeared to resume in 2013 and 2014, although sampling was scarcer.

### On small-scale structure and migration

In Orsay, the two sets we intensively sampled at a small spatial scale (four groups of five apples) contained several haplotypes (Félix and Duveau 2012) (Figure S6). In most cases, when two haplotypes were found locally in the same group of apple (within 30 cm), they were also found mixed within an apple (six instances). This result suggests that a single apple may often be colonized by several immigrating individuals.

In Santeuil, the co-occurrence of two haplotypes in a sample was low in the first year, corresponding to the strong spatial structure at the scale of 10–50 m along the transect. However, when two haplotypes were present within a few meters (often so in 2010 and 2011), they were also frequently found in the same sample (Table S6). Accordingly, the proportion of the differentiation among samples as estimated by the weighted Weir Cockerham *Fst*, which was 0.92 in 2009 and sharply decreased to 0.45 in 2010 and 0.26 in 2011 (139, 52, and 107 individuals and 29, 10, and 21 samples, respectively; numbers insufficient in later years).

The third location, Plougasnou, was only sampled once and yielded five different haplotypes, the major one being HP1 (64% individuals in six out of seven samples). Remarkably, a mix of five haplotypes was found in a single sample (P142, Figure S8 and Table S7).

In summary, in all three locations, different haplotypes were frequently found in a given sample, especially when present very locally within a few square meters.

Invertebrates being potential carriers, we also genotyped a few *C. elegans* individuals found on invertebrates. Their genotypes reflected what was sampled close by, for example, a slug on apple O831 (both carrying a rare haplotype; Table S5). We found a snail with two *C. elegans* genotypes (S162A, Table S6). The samples of leaf litter and soil that had *C. elegans* also generally carried the same genotypes as the stems around them (Table S6).

**Figure 4:**
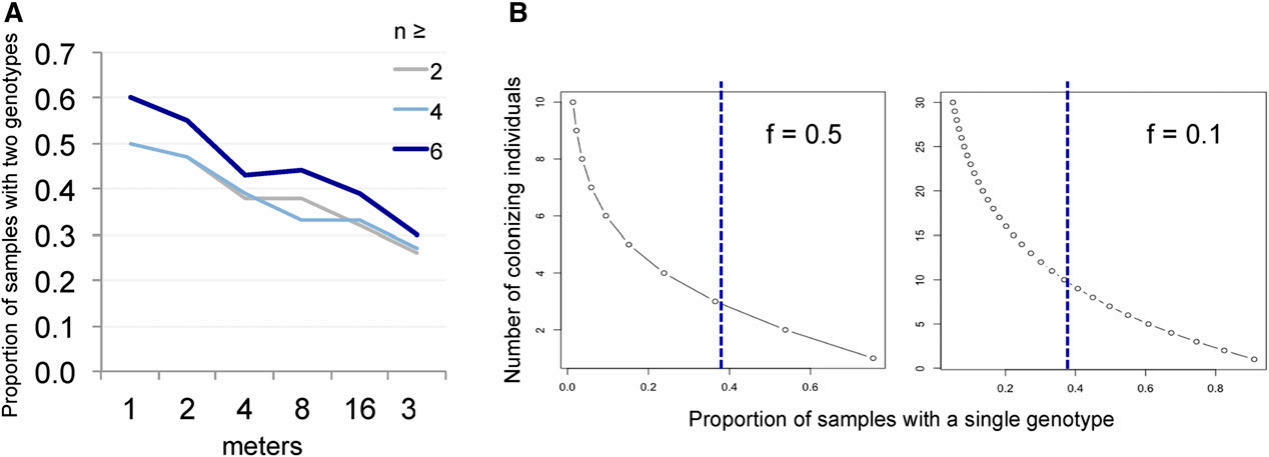
Co-occurrence and colonization. (A) Probability that two distinct haplotypes found at less than a given distance (*x* axis) are present in a sample, taking into account samples with ≥*n* genotyped individuals (color coded). (B) Graphs of number of colonizing individuals required to explain the data, if the frequency of both genotypes was always close to 0.5 each (left) or if the minor allele frequency was 0.1 (right). For random distributions of allele frequency, see text. The blue dashed line corresponds to the estimated proportion of samples with a single genotype.

We plotted the frequency of detection of two *C. elegans* haplotypes in a rotting fruit or stem sample given the presence of these two haplotypes within a given distance (Figure 4). This probability of co-occurrence of two haplotypes in the same rotting fruit or stem sample was high, especially when the two haplotypes were detected within a short distance, suggesting that each stem or fruit subpopulation was seeded by more than one individual. This probability of co-occurrence decreased with increasing distance at which two different haplotypes were found, strongly so at distances >10 m. As the number of genotyped individuals was low in some instances, the proportion is an underestimate. To account for this, assuming a random uniform distribution of genotype frequency in samples, we estimated the proportion of samples with more than one genotype to be 0.62 (± 0.04 SD) for the 1-m scale.

Keeping these data at the smallest scale, we then modeled using a Poisson distribution the number of colonizing individuals that could account for the proportion of 62% of the samples with more than one genotype, given the presence of two genotypes within 1 m. Depending on how the local haplotype frequencies are modeled (see *Materials and Methods*), the median number of the lambda coefficient of the Poisson distribution varied between 3 and 10. We thus conclude that in the context of a metapopulation with our samples corresponding to a deme or subpopulation, the demes are generally seeded by a mean of 3–10 immigrating individuals.

### Rare outcrossing

Despite the co-occurrence of different haplotypes in the same sample, we found very few heterozygotes, confirming our previous measures of a low outcrossing rate (Barrière and Félix 2005, 2007). We found few cases of populations with males or mated adult hermaphrodites (the latter inferred from the presence of many males in their progeny). From the genotyping results, some of these examples corresponded to crosses between hermaphrodites and males of similar genotype (Félix and Duveau 2012; Table S5, Table S6, and Table S7).

We found only three heterozygotes, all in Santeuil, corresponding to each pairwise combination between HS1, HS2, and HS3. Two were isolated as larvae (L1 and dauer) and the third as a mated (plugged) adult. The latter gave rise to many F1 males, thus in this very particular case the heterozygous genotype is likely that of the pooled progeny and not necessarily that of the sampled adult. Most surprisingly, we did not observe recombinants between haplotypes on the timescale of our study. The exception was a single individual in 2011 (S169.2), with a HS2 genotype over the whole genome except a right tip of chromosome V from HS3 (Table S3 and Table S6).

In Orsay, we did not detect any heterozygote. We further tested whether the six minor haplotypes that appeared after the first year could have been derived by local recombination. Five of them (HO9-13; the case is less clear for HO8) contained new immigrating alleles from other locations (this work; Cook *et al.* 2016), thus likely were not derived solely by local recombination (however we cannot rule out that they were products of recent recombinations with a very minor haplotype).

Overall, our results confirm previous results in these habitats showing a low level of outcrossing and very low effective recombination.

### Phenotypic characterization of Orsay isolates

Intrigued by the maintenance over years of minor haplotypes in Orsay, we sought to characterize the animals at the phenotypic level, focusing on this population. Especially, we wondered whether heterogenous selection in different environments and/or stages of the lifecycle could explain the maintenance of minor haplotypes.

**Figure 5:**
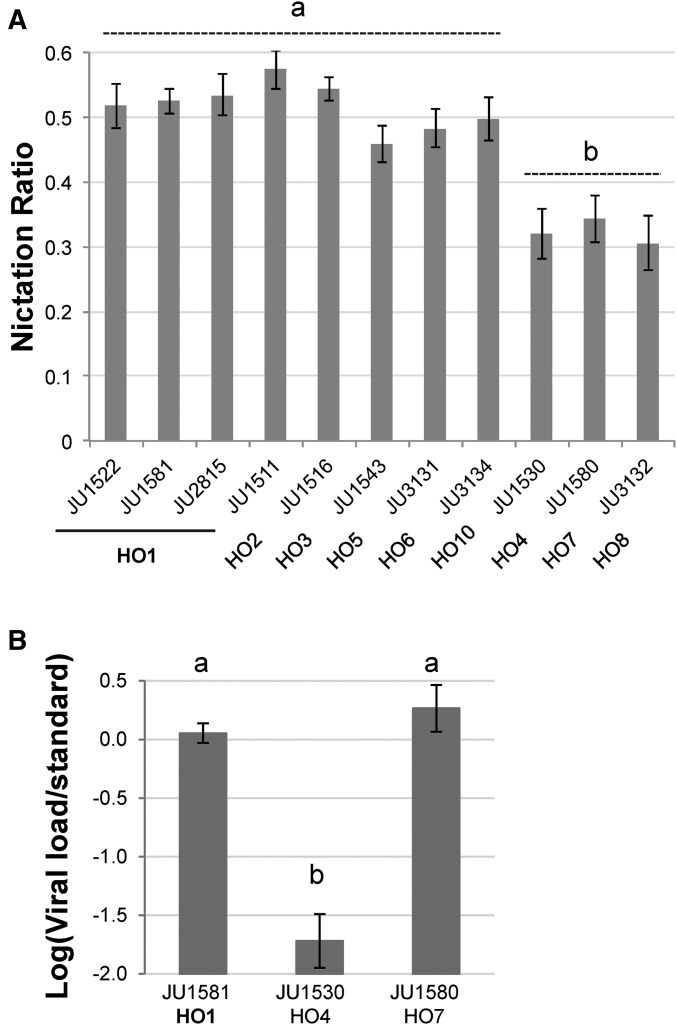
The Orsay strains differ from each other phenotypically. (A) Nictation ratio. The graph presents the mean nictation ratio, with bars indicating the SE over eight replicates. The haplotype groups are indicated under the graph below the strain name. (B) Viral load after laboratory infection with the Orsay virus, with bars indicating the SE over three replicates. Data replotted from data in Ashe *et al.* (2013). The Tukey *post-hoc* test groups (a and b) are indicated above each graph.

As a proxy for the ability to migrate between food sources and be carried away by a vector, we measured the capacity to nictate of dauer larvae of representative strains of haplotype HO1 (two from year 2008, one from year 2014) as well as representatives of minor haplotypes such as HO4 and HO7 (Figure 5A). We found a significant difference in the nictation ratio of the different haplotypes (*P* = 10^−14^). All three HO1 strains were in the group with the highest nictation ratio, which contrasted with the much lower nictation level of JU1530 (HO4) and some other minor haplotypes (HO7 and HO8). In conclusion, phenotypic diversity in nictation ratio can be observed among the Orsay haplotypes.

The Orsay virus was found on animals with the HO1 (three times) and HO7 (once) haplotype but none of the other haplotypes (L. Frézal and M.-A. Félix, personal communication). We assayed viral load after experimental infection by the Orsay virus in a worldwide set of *C. elegans* isolates that included three Orsay haplotypes (Ashe *et al.* 2013). Although JU1581 (HO1) and JU1580 (HO7) were among those showing the highest viral load, JU1530 (HO4) was able to replicate the virus at a much lower rate (Figure 1S1A in Ashe *et al.* (2013)) (Figure 5B). We conclude that a large variation in immunity to the Orsay virus is present in the Orsay orchard.

**Figure 6:**
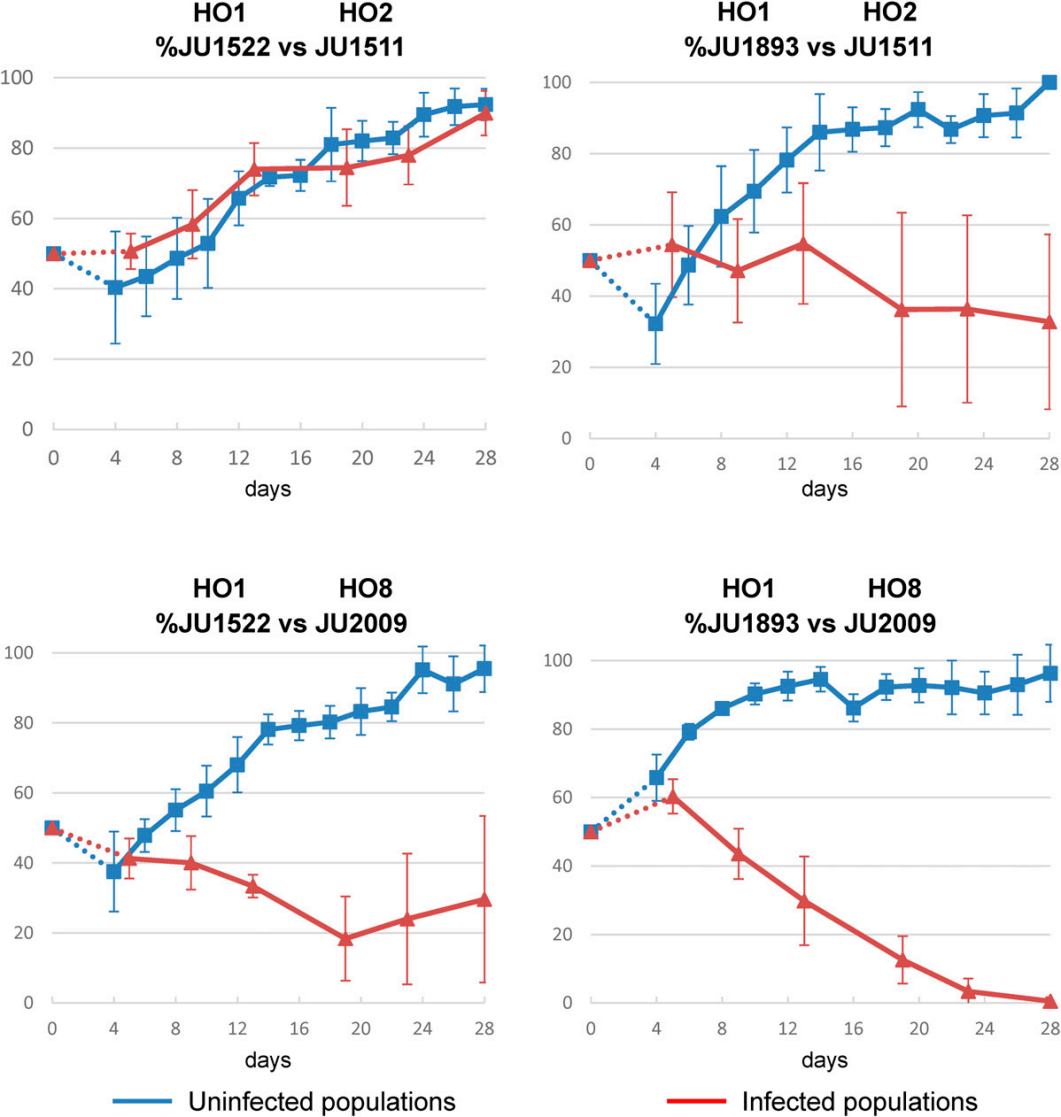
Experimental competition between Orsay haplotypes in two environments reveals heterogeneous selection. Each graph represents two competition assays between one HO1 strain and another strain (HO2 or HO8). The mean proportions (*y* axis) of five replicates of each treatment are indicated at each transfer. Error bars are SE over replicates. Blue lines are competition assays without microsporidia infection; red lines are competition assays in the presence of the microsporidia *N. ausubeli* JUm2009 (isolated from *C. elegans* JU2009 with the HO8 haplotype). The *x* axis represents the number of days of the competition assays. Each graph represents a competition assay between one HO1 strain and another strain (HO2 or HO8) (four pairs). The mean proportions (*y* axis) of five replicates of each treatment are indicated at each transfer. Error bars are SE over replicates. On the left are competition assays without microsporidia infection; on the right are competition assays in the presence of the microsporidia *N. ausubeli* JUm2009 (from *C. elegans* JU2009 with HO8 haplotype). The *x* axis represents the number of transfers. All competitions were performed in parallel.

The microsporidia *N. parisii* was found in Orsay on animals with the HO1 (once, JU1893) and HO13 (once, JU2816) haplotypes; *N. ausubeli* was found on animals with the HO2 (once, JU2671) and HO8 (once, JU2009) haplotypes. As 77% of sampled *C. elegans* in Orsay are of the HO1 haplotype but only one of those was detected with microsporidia infection, we wondered whether HO1 strains were particularly resistant or sensitive to microsporidia infection. Competition assays were thus performed between two different HO1 strains and one other strain representing the HO2 or HO8 haplotypes, in the presence or absence of microsporidia. Without microsporidia, the HO1 strain won over all other tested strains (Figure 6 and Table S8). With microsporidia infection, however, HO2 and especially HO8 strains showed higher resistance to microsporidia infection than HO1 strains. Most strikingly, in the competition between JU1893 (HO1) and JU2009 (HO8), JU2009 was fixed in all five replicates with microsporidia infection, whereas in the absence of microsporidia, JU1893 was always fixed (Figure 6 and Table S8). In conclusion, microsporidia infection reveals a possible source of varying selection that may explain the maintenance of minor haplotypes.

### Known causal polymorphisms

The *drh-1* deletion generally renders *C. elegans* sensitive to viral infection (Ashe *et al.* 2013). Out of the strains that were tested for viral resistance above, the sensitive HO1 and HO7 haplotypes carry the deletion, whereas the resistant HO4 has an intact *drh-1* gene, like many of the minor haplotypes in Orsay (Table S9). The *drh-1* deletion is fixed in Santeuil and Plougasnou.

The *zeel-1peel-1* deletion polymorphism was found to be involved in an incompatibility between genotypes (Seidel *et al.* 2008, 2011). We find that the *zeel-1peel-1* insertion was mostly fixed in each local population, but not completely so in Orsay, where the minor haplotypes HO6 and HO13 do not carry either of the two genes (Table S9). This shows that this polymorphism exists locally and may potentially result in hybrid dysgenesis and preferential spread of the insertion if the two genotypes happen to mate.

*C. elegans* chromosome centers display a much lower recombination rate than the arms. In a worldwide set of *C. elegans* wild isolates, large central regions of chromosomes I, IV, and V were shown to present imprints of recent selective sweeps (Andersen *et al.* 2012). Remarkably, the main Orsay haplotype HO1 retained a divergent chromosome I center region, whereas all other haplotypes in Orsay and elsewhere have the swept version (except HO11). In Santeuil, the most common haplotype HS2 (called HO7) has an unswept chromosome V, whereas HS1 and HS3 have the swept version. These two distinct major local haplotypes may perhaps have so far avoided the sweep because they are well adapted locally, better so than the genotype with the invading chromosomal region.

## Discussion

In this study, we report for the first time on the local structure of *C. elegans* populations. Compared with previous studies focusing on compost heap populations, our study has four novel components. First, we study the local substructure at the scale of 1–100 m, which could not be done in a compost heap. Second, we genotype individuals from demographically characterized subpopulations sampled from substrates on which they feed and reproduce. Third, we study populations that are not necessarily doomed to extinction, unlike those in compost heaps. Finally, we complement the study of genetic polymorphisms with the demonstration of local phenotypic polymorphism in ecologically relevant traits that concern the different metapopulation phases.

We can now answer the so far unanswered questions raised in the introduction: Can distinct genotypes be found within a single booming, reproductively active *C. elegans* subpopulation? Yes: distinct genotypes compete in a given stem or fruit in the feeding phase, as they also do in the migration phase. Given this co-existence, are heterozygote and recombination frequencies high? No: we detect a few rare heterozygotes but no obvious recombinants, except for a furtive singleton. Do *C. elegans* genotypes of the previous year reseed the orchard populations when they reappear in the fall? Yes: some full haplotypes occur locally stably over years, suggesting that they are maintained locally during the winter. It is still unclear where they may be found, maybe in host invertebrates or at low density in soil.

In addition, we could estimate for the first time the size of the bottleneck seeding each *C. elegans* subpopulation, given the observed intrademe diversity. Spatial structure is visible within a location (30–100 m) at some timepoints, but remarkably fades at a lower scale of 1–10 m, where several genotypes may coexist. Given this local occurrence and the intrademe diversity of genotypes, we estimated that an average of 3–10 incoming immigrating individuals colonize a given fruit or stem. These numbers make it plausible that a single invertebrate (isopod, slug, *etc*.) may generally be the vector of these several *C. elegans* individuals seeding a given substrate. As an important consequence of this bottleneck size, two (or more) *C. elegans* genotypes may often compete and coexist within a blooming population. They may thus interact with each other regarding feeding, dauer entry and exit, horizontal pathogen transmission, *etc*.

Our results also have implications concerning the very low level of outcrossing and recombination of the species. Despite the mixing of genotypes in a given sample, we found few heterozygotes in these actively reproducing populations and no obvious case of lasting recombinant over a few years. This is consistent with our previous results following compost populations over years (Barrière and Félix 2007), and with the low level of effective recombination in the whole species (Rockman and Kruglyak 2009; Andersen *et al.* 2012). What is new is that we could show that this coexistence without outcrossing occurred in feeding and reproducing populations and not only in dauer populations. Thus, the low frequency of outcrossing is more clearly demonstrated.

Low levels of outcrossing build coadapted multilocus combinations, which in turn results in selection against heterozygotes or recombinants (outbreeding depression; Barrière and Félix 2007; Dolgin *et al.* 2007). The low effective recombination that we observe despite the presence of some heterozygotes is consistent with the outbreeding depression observed among wild *C. elegans* strains (Dolgin *et al.* 2007). Interrogating an obvious candidate locus disfavoring outcrossing, the *zeel-1peel-1* locus, we found that the Orsay location included both alleles (presence and absence) of these linked incompatibility genes, but the allele corresponding to their absence only occurred at very low frequency. Moreover, the *zeel-1peel-1* polymorphism was absent in Santeuil (the insertion is fixed), thus outbreeding depression must be explained by other causes. The present strain collection will be a useful tool to assess local outbreeding depression and its mechanistic causes.

We did not focus on the differentiation and comparison among locations. However, we note that, as reported before (Barrière and Félix 2005, 2007; Haber *et al.* 2005; Sivasundar and Hey 2005), we observe population differentiation among locations. We emphasize, contra to Volkers *et al.* (2013), that this population differentiation does not demonstrate local adaptation, since a mosaic pattern can also be obtained with a neutral model and limited migration. In addition, we observe as previously that each local population displays polymorphism mostly from immigrating alleles, whereas the number of distinct haplotypes is very limited.

Local coexistence of haplotypes could be driven by neutral processes or by heterogeneous selection favoring one genotype in one environment and another genotype in another environment. In a metapopulation, possibly some genotypes are better at dispersal and others are better at exponential growth competition. For example, the Orsay HO1 genotype appears better at dauer nictation, a behavior favoring dispersal, than the HO4 genotype, whereas the latter is more resistant to the Orsay virus and may outcompete HO1 in exponential growth in the presence of virus. We indeed previously showed using the JU1580 HO7 genotype (with the *drh-1* deletion, like HO1) that viral infection results in a slower progeny production and a lower brood size and diminishes competitive fitness compared with the *drh-1* rescue strain (Ashe *et al.* 2013). Furthermore, we showed that Orsay HO1 strains win in the microsporidia-free environment, whereas HO8 wins in the presence of microsporidia, suggesting that microsporidia-resistant haplotypes such as HO8 could persist due to selection in the presence of microsporidian parasites. Our results suggest the presence of local phenotypic polymorphisms in traits on which natural selection may act, with a coexistence of genotypes that may each be more successful in a different local subenvironment. Our collection can be further probed for such polymorphisms, associated trade-offs, and possible frequency-dependent selection.

Besides competition and crosses among genotype groups, clonal evolution may also occur within a haplotype group undergoing a mutation-selection process. The natural metapopulation structure with bottlenecks and recovery is in principle remarkably similar to laboratory experiments of mutation accumulation at small population size followed by re-expansion (Estes and Lynch 2003). Outcrossing may be effective at this intralineage evolutionary scale (de Visser and Elena 2007; Anderson *et al.* 2010). We did not explore this “nanoevolutionary” dimension here, but our collection provides a excellent tool to do so in the future using whole-genome sequencing approaches.

## Supplementary Material

Supplemental material is available online at www.genetics.org/lookup/suppl/doi:10.1534/genetics.117.300564/-/DC1.
