# Supplementary material for: The Local Coexistence Pattern of Selfing Genotypes in Caenorhabditis elegans Natural Metapopulations
Source: Genetics. 2017 Dec 12;208(2):807–21. doi: 10.1534/genetics.117.300564 (PMC5788539; doi:10.1534/genetics.117.300564)
Supplement: Supplementary file 1 [file 807FigureS5.pdf]

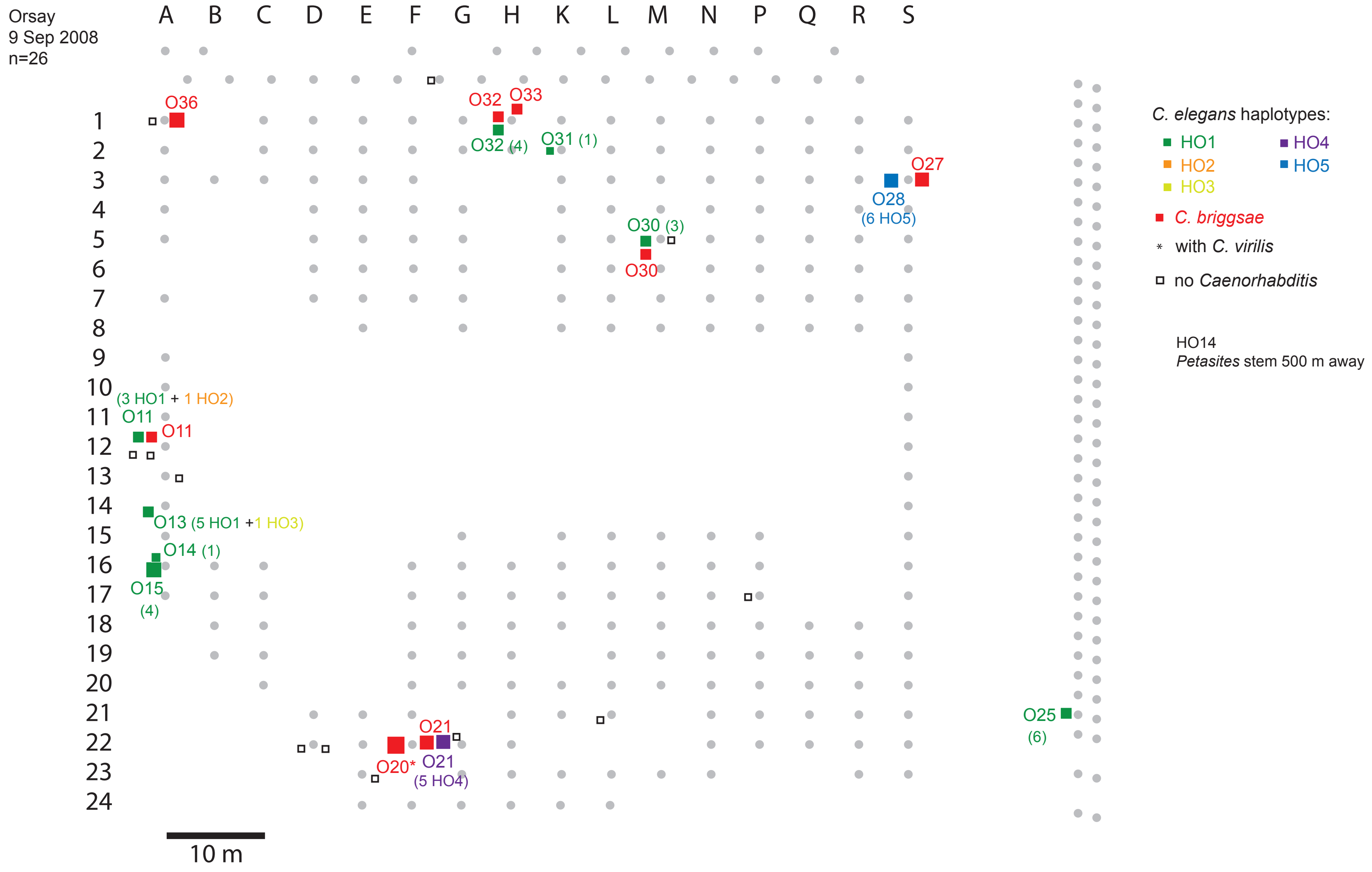

Orsay
9 Sep 2008
n=26
A B C D E F G H K L M N P Q R S
1 2 3 4 5 6 7 8 9 10 11 12 13 14 15 16 17 18 19 20 21 22 23 24
O36
O32
O33
O32 (4)
O31 (1)
O27
O28
(6 HO5)
O30 (3)
O30
(3 HO1 + 1 HO2)
O11
O11
O13 (5 HO1 +1 HO3)
O14 (1)
O15
(4)
O21
O20*
O21
(5 HO4)
O25
(6)
10 m
C. elegans haplotypes:
HO1
HO2
HO3
HO4
HO5
C. briggsae
with C. virilis
no Caenorhabditis
HO14
Petasites stem 500 m away

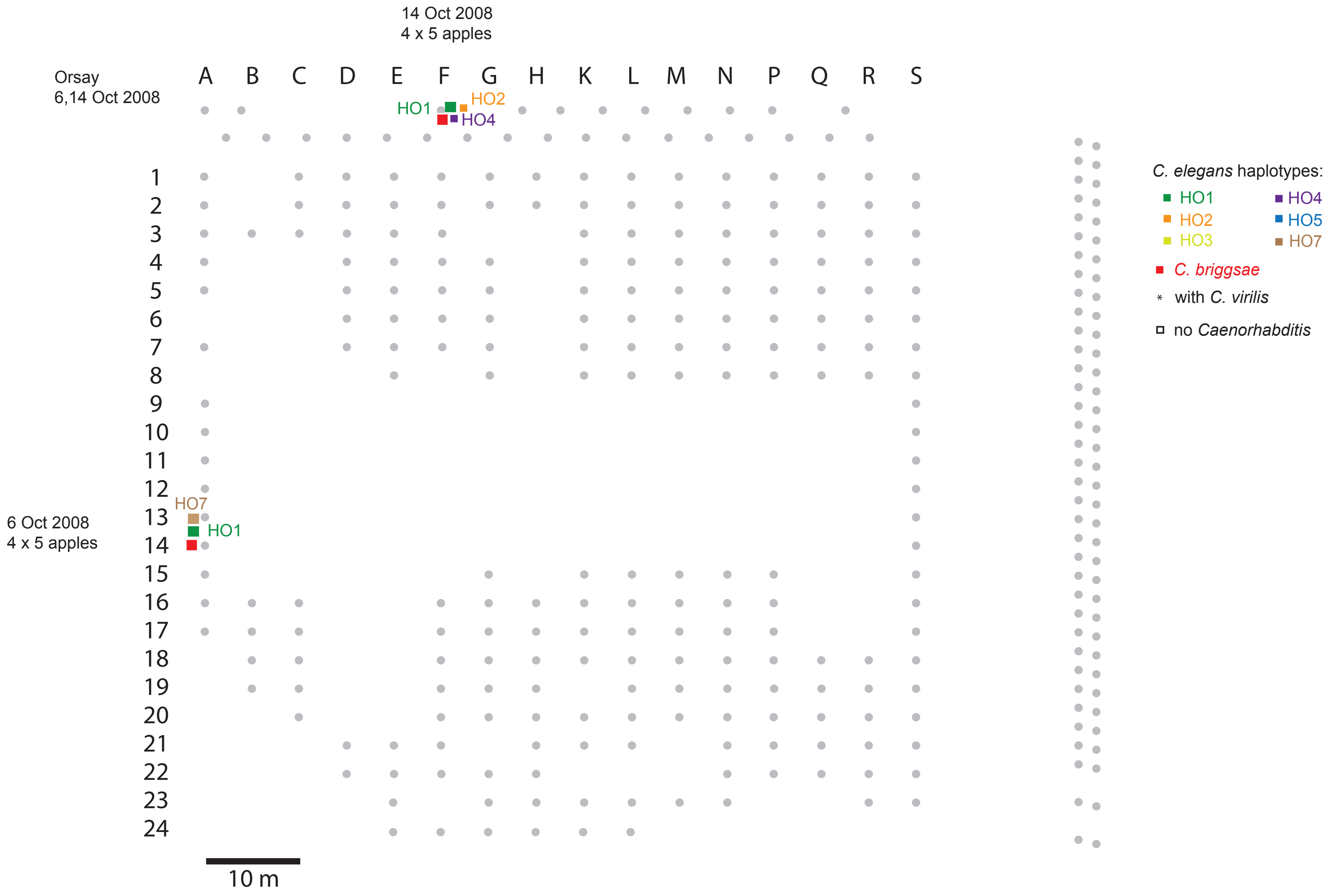
14 Oct 2008
4 x 5 apples
Orsay
6,14 Oct 2008
A B C D E F G H K L M N P Q R S
HO1
HO2
HO4
1 2 3 4 5 6 7 8 9 10 11 12 13 14 15 16 17 18 19 20 21 22 23 24
HO7
6 Oct 2008
4 x 5 apples
HO1
10 m
C. elegans haplotypes:
HO1
HO2
HO3
HO4
HO5
HO7
C. briggsae
with C. virilis
no Caenorhabditis

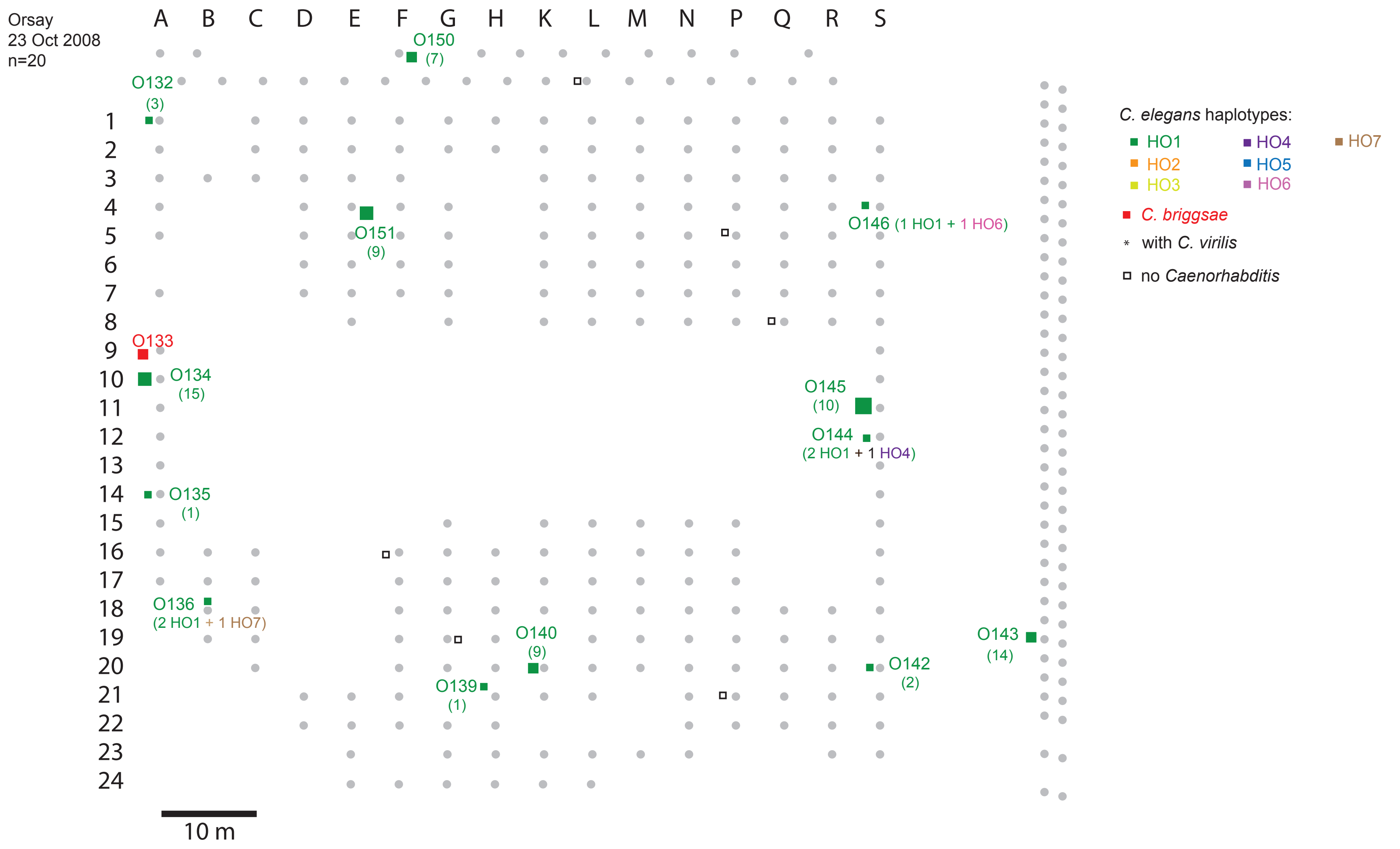
Orsay
23 Oct 2008
n=20
A B C D E F G H K L M N P Q R S
1 2 3 4 5 6 7 8 9 10 11 12 13 14 15 16 17 18 19 20 21 22 23 24
O150 (7)
O132 (3)
O151 (9)
O146 (1 HO1 + 1 HO6)
O133
O134 (15)
O145 (10)
O144 (2 HO1 + 1 HO4)
O135 (1)
O136 (2 HO1 + 1 HO7)
O140 (9)
O139 (1)
O142 (2)
O143 (14)
10 m
C. elegans haplotypes:
HO1
HO2
HO3
HO4
HO5
HO6
HO7
C. briggsae
with C. virilis
no Caenorhabditis

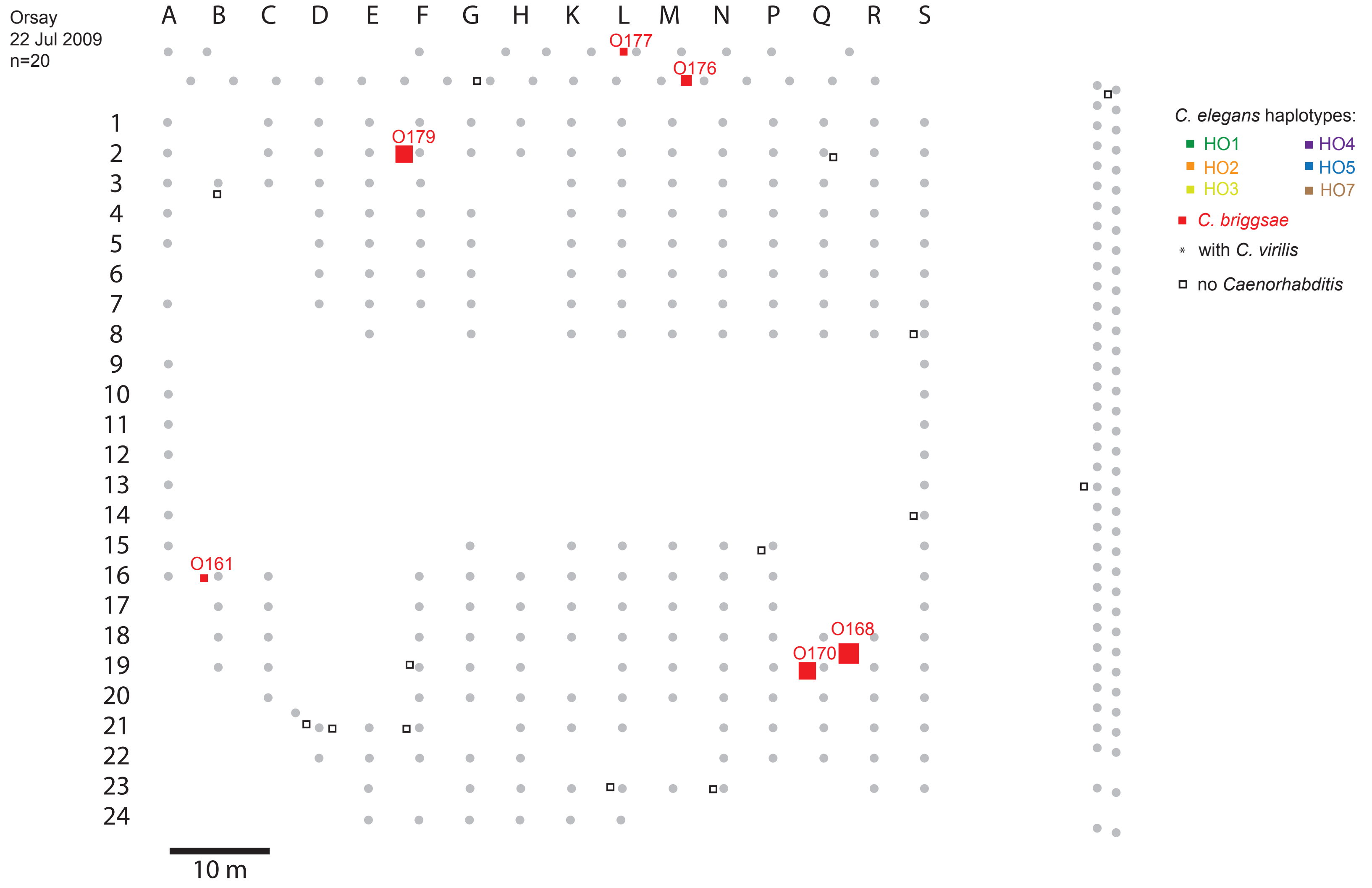

Orsay
22 Jul 2009
n=20
A B C D E F G H K L M N P Q R S
1 2 3 4 5 6 7 8 9 10 11 12 13 14 15 16 17 18 19 20 21 22 23 24
O177
O176
O179
O161
O168
O170
10 m
C. elegans haplotypes:
HO1
HO2
HO3
HO4
HO5
HO7
C. briggsae
with C. virilis
no Caenorhabditis

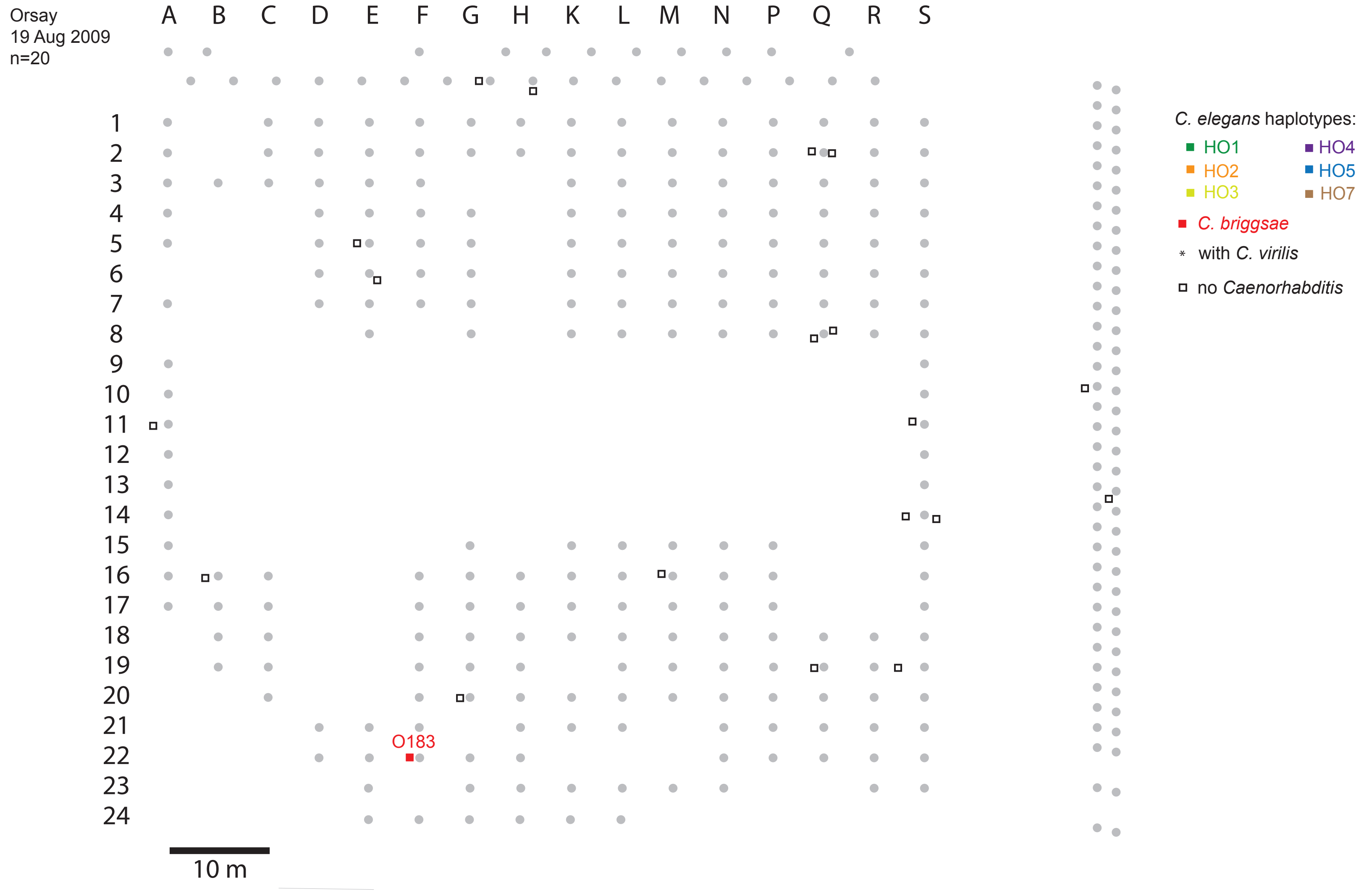

Orsay
19 Aug 2009
n=20
A B C D E F G H K L M N P Q R S
1 2 3 4 5 6 7 8 9 10 11 12 13 14 15 16 17 18 19 20 21 22 23 24
O183
10 m
C. elegans haplotypes:
HO1
HO2
HO3
HO4
HO5
HO7
C. briggsae
* with C. virilis
no Caenorhabditis

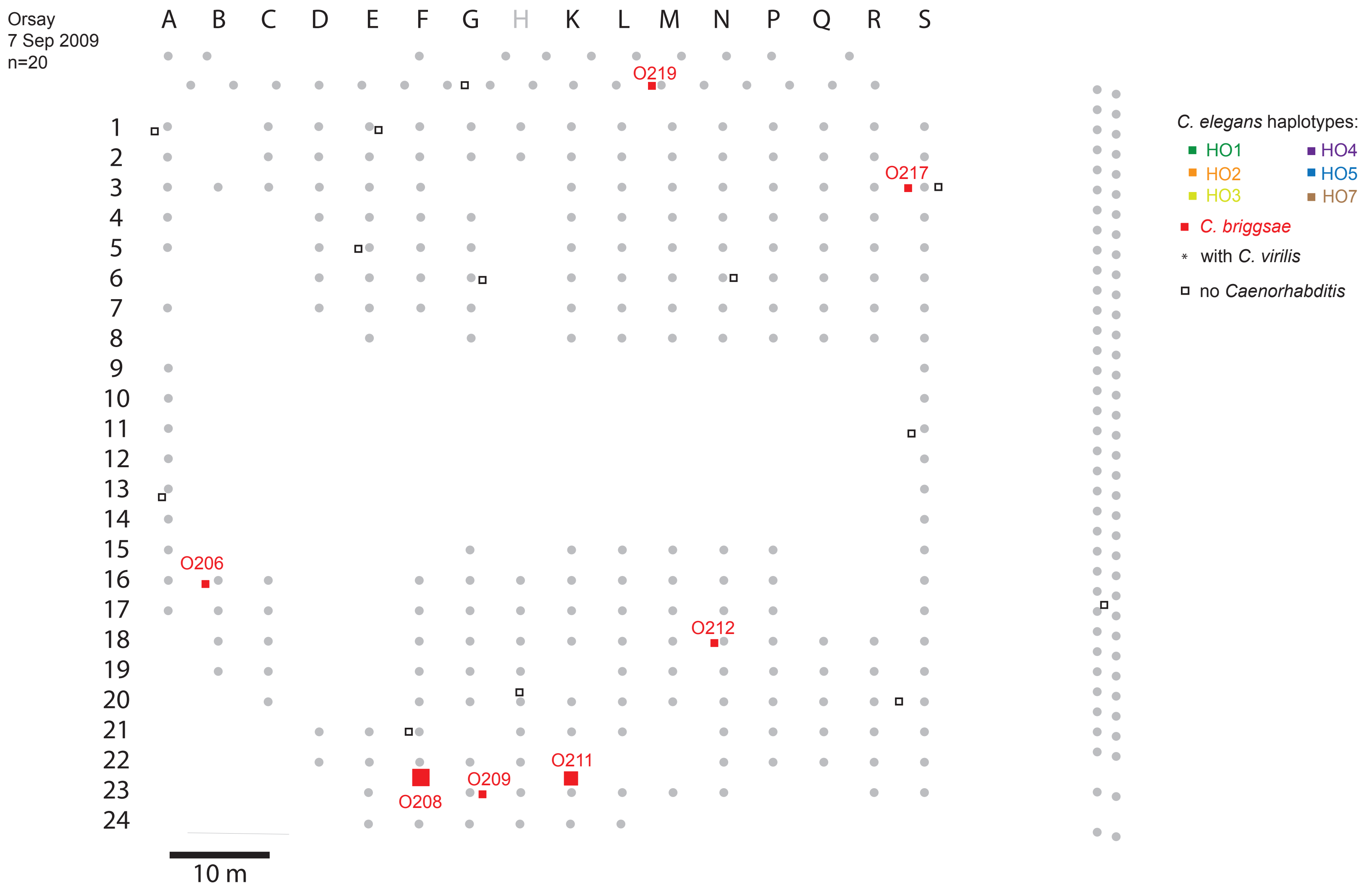
Orsay
7 Sep 2009
n=20
A B C D E F G H K L M N P Q R S
1 2 3 4 5 6 7 8 9 10 11 12 13 14 15 16 17 18 19 20 21 22 23 24
O219
O217
O206
O212
O208
O209
O211
10 m
C. elegans haplotypes:
HO1
HO2
HO3
HO4
HO5
HO7
C. briggsae
with C. virilis
no Caenorhabditis

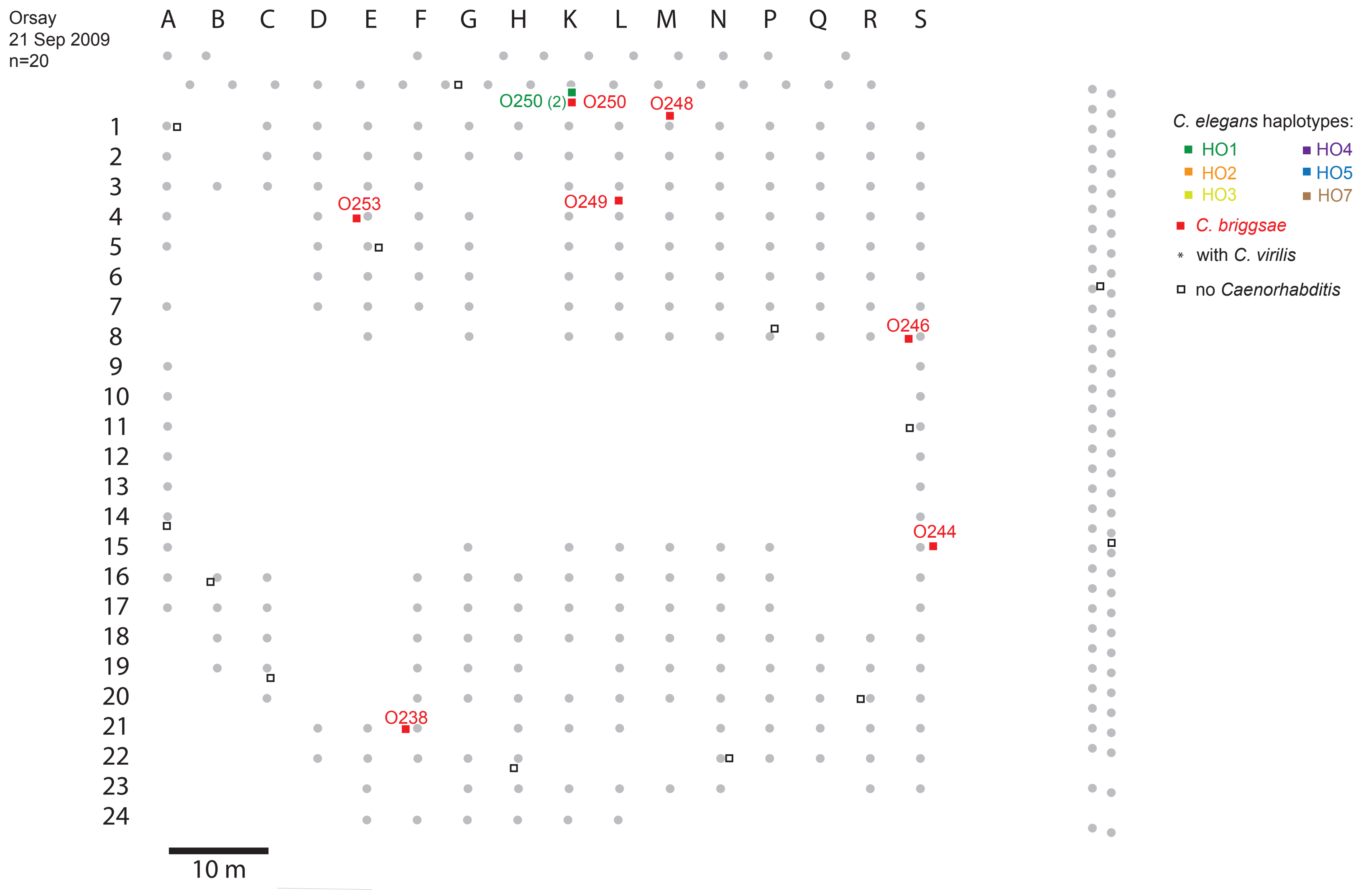
Orsay
21 Sep 2009
n=20
A B C D E F G H K L M N P Q R S
1 2 3 4 5 6 7 8 9 10 11 12 13 14 15 16 17 18 19 20 21 22 23 24
O250 (2)
O250
O248
O253
O249
O246
O244
O238
10 m
C. elegans haplotypes:
HO1
HO2
HO3
HO4
HO5
HO7
C. briggsae
with C. virilis
no Caenorhabditis

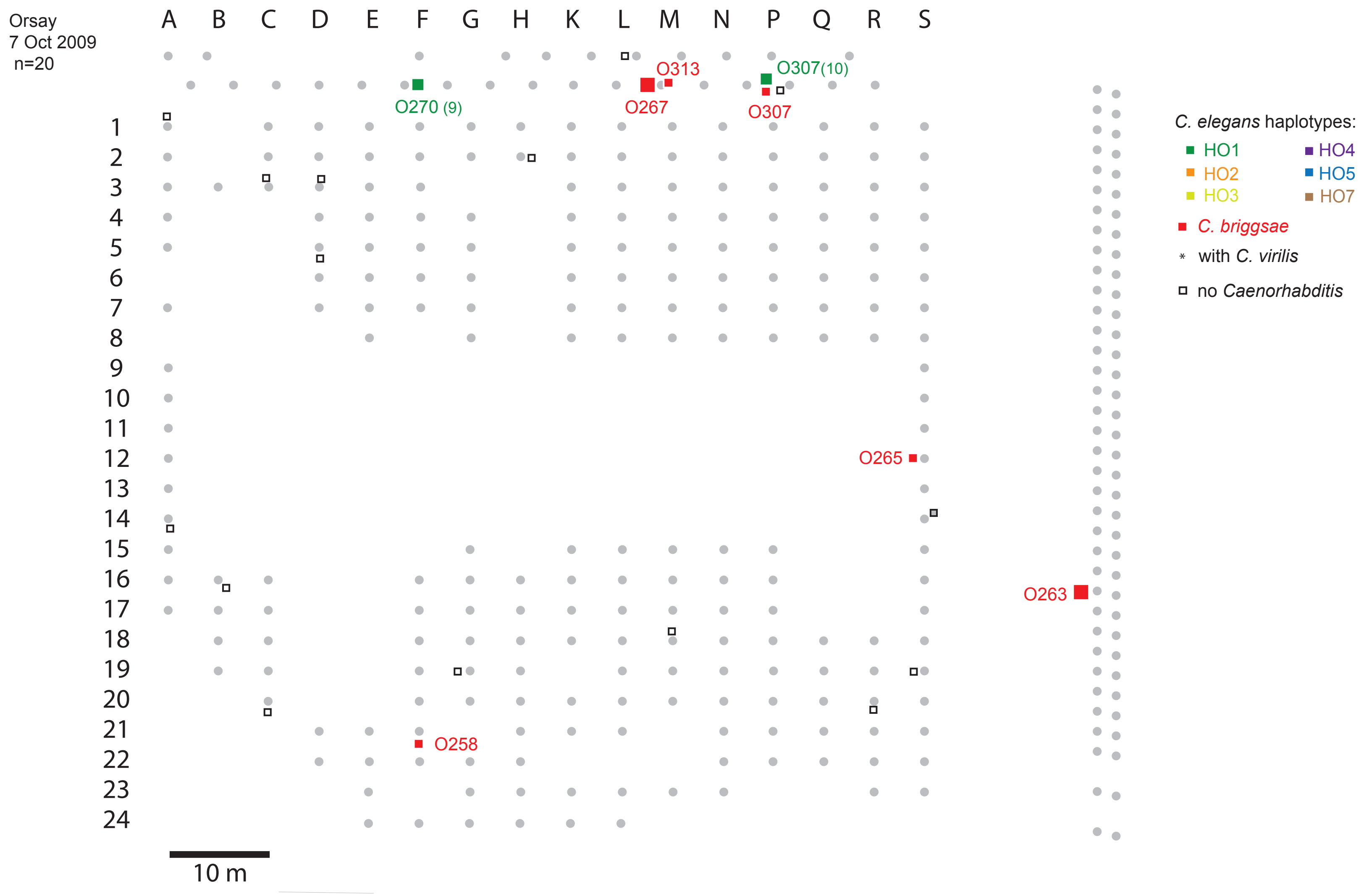

Orsay
7 Oct 2009
n=20
A B C D E F G H K L M N P Q R S
1 2 3 4 5 6 7 8 9 10 11 12 13 14 15 16 17 18 19 20 21 22 23 24
O270 (9)
O267
O313
O307(10)
O307
O265
O263
O258
10 m
C. elegans haplotypes:
HO1
HO2
HO3
HO4
HO5
HO7
C. briggsae
with C. virilis
no Caenorhabditis

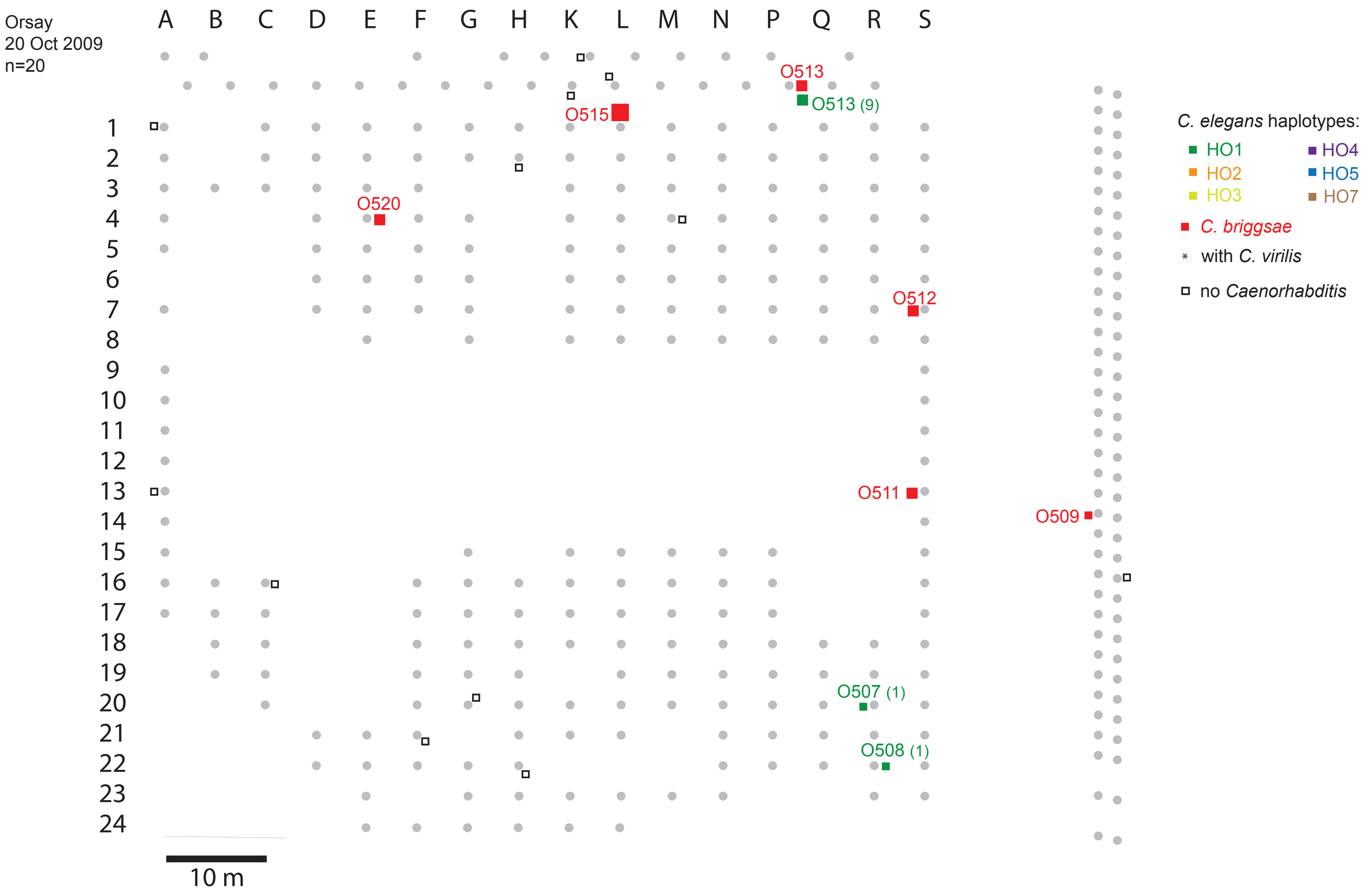
Orsay
20 Oct 2009
n=20
A B C D E F G H K L M N P Q R S
1 2 3 4 5 6 7 8 9 10 11 12 13 14 15 16 17 18 19 20 21 22 23 24
O513
O513 (9)
O515
O520
O512
O511
O509
O507 (1)
O508 (1)
10 m
C. elegans haplotypes:
HO1
HO2
HO3
HO4
HO5
HO7
C. briggsae
with C. virilis
no Caenorhabditis

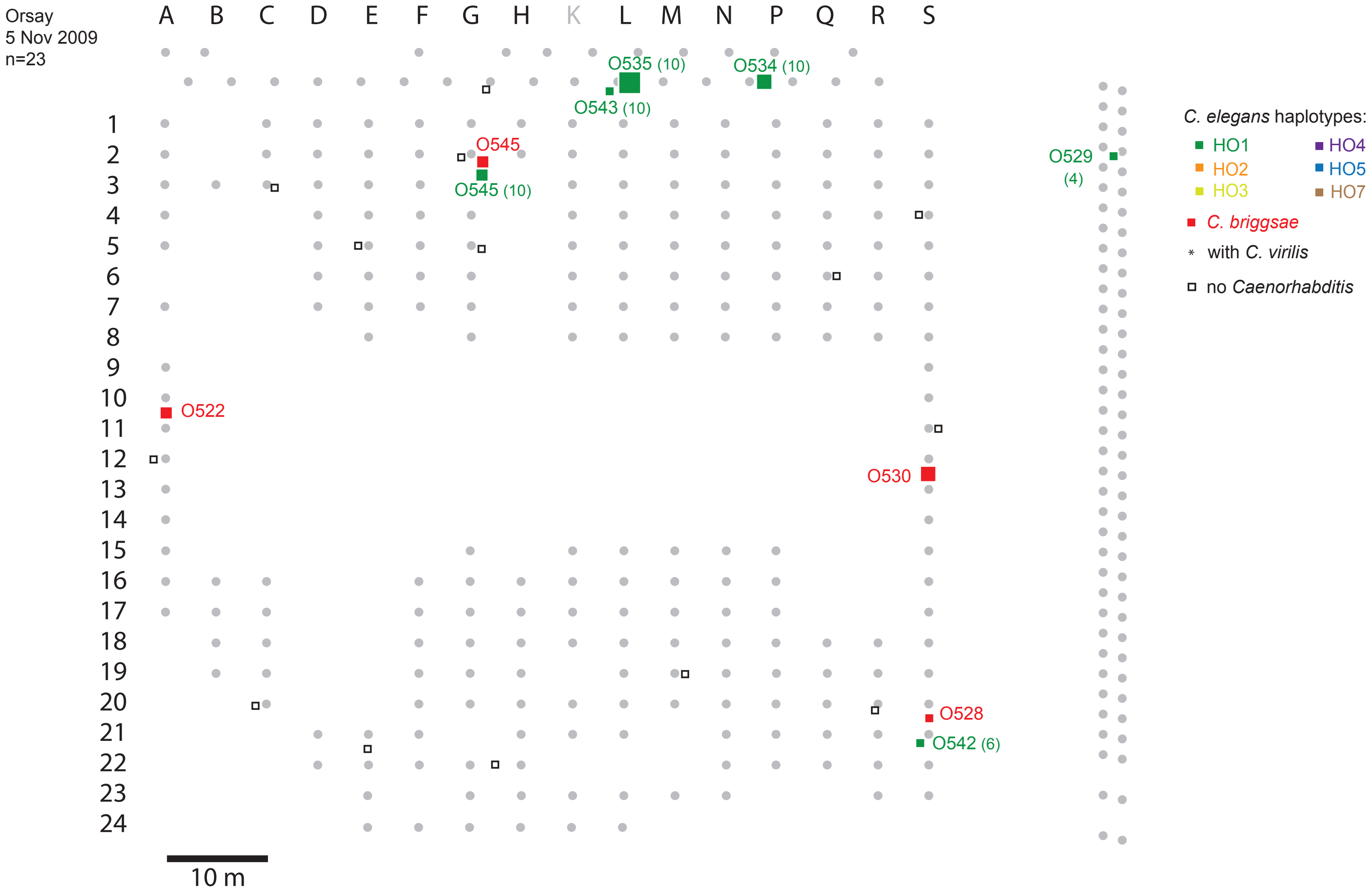
Orsay
5 Nov 2009
n=23
A B C D E F G H K L M N P Q R S
1 2 3 4 5 6 7 8 9 10 11 12 13 14 15 16 17 18 19 20 21 22 23 24
O535 (10)
O534 (10)
O543 (10)
O545
O545 (10)
O529
(4)
O522
O530
O528
O542 (6)
10 m
C. elegans haplotypes:
HO1
HO2
HO3
HO4
HO5
HO7
C. briggsae
with C. virilis
no Caenorhabditis

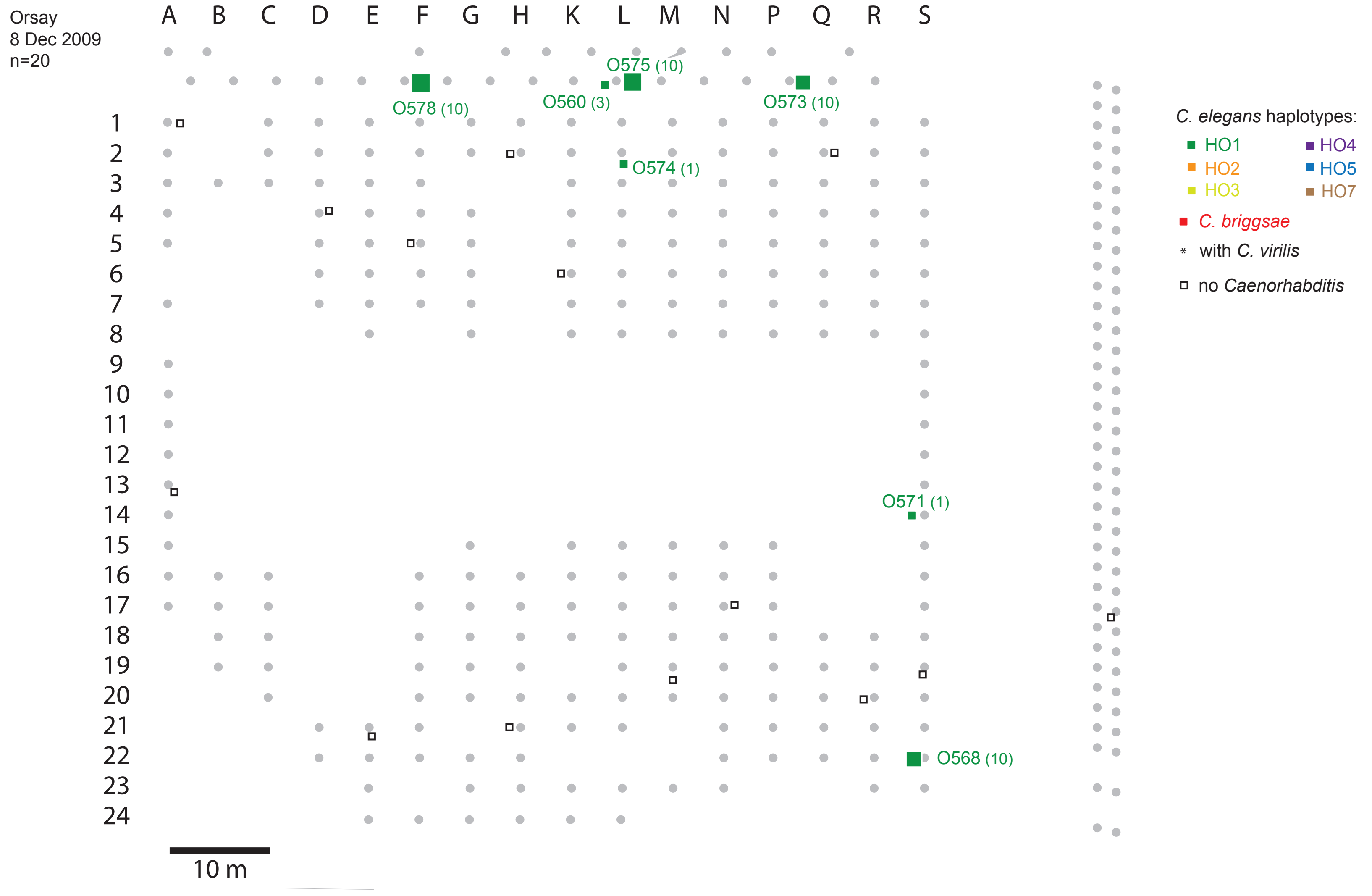
Orsay
8 Dec 2009
n=20
A B C D E F G H K L M N P Q R S
1 2 3 4 5 6 7 8 9 10 11 12 13 14 15 16 17 18 19 20 21 22 23 24
O578 (10)
O560 (3)
O575 (10)
O573 (10)
O574 (1)
O571 (1)
O568 (10)
10 m
C. elegans haplotypes:
HO1
HO2
HO3
HO4
HO5
HO7
C. briggsae
with C. virilis
no Caenorhabditis

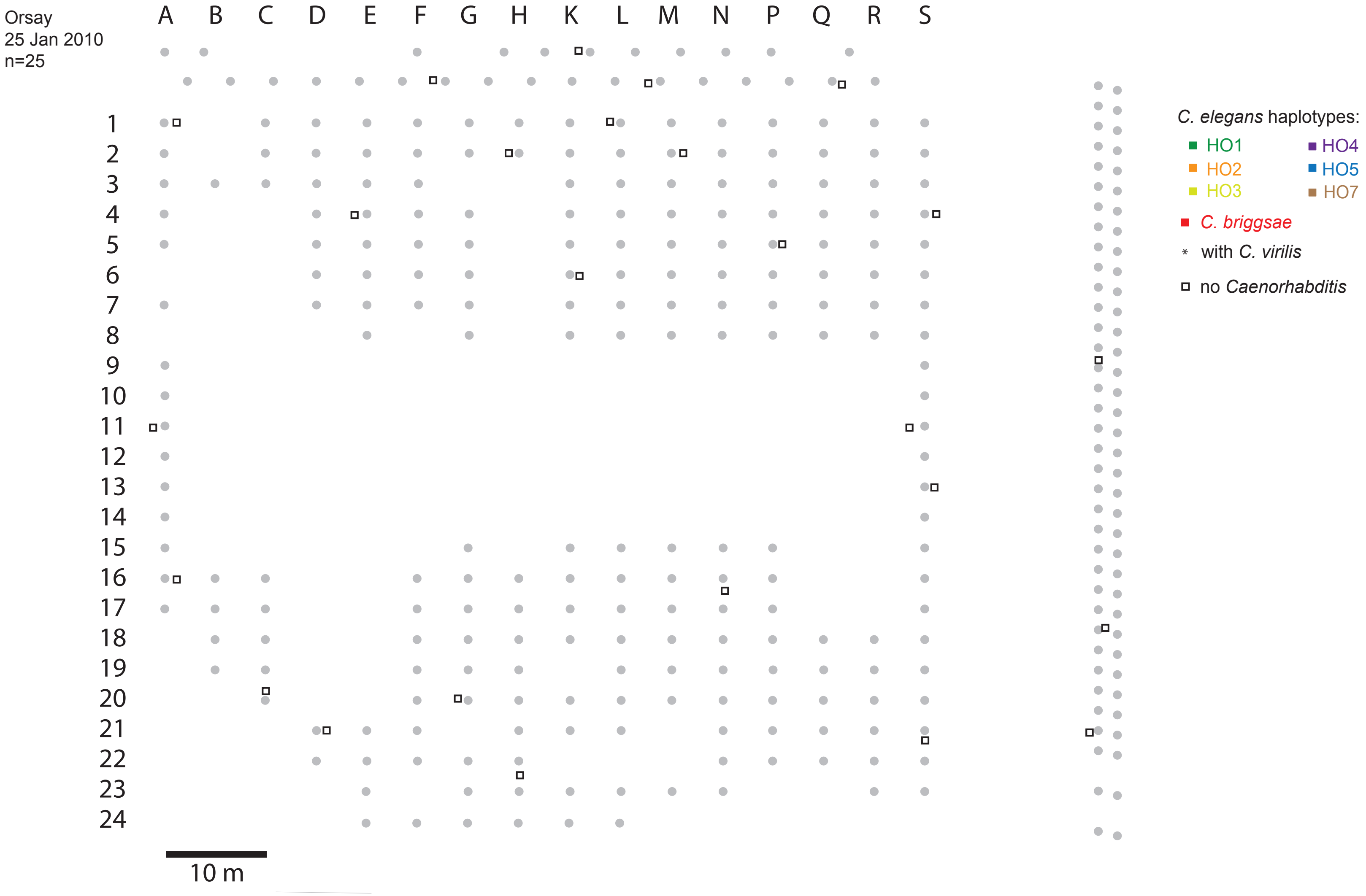
Orsay
25 Jan 2010
n=25
A B C D E F G H K L M N P Q R S
1 2 3 4 5 6 7 8 9 10 11 12 13 14 15 16 17 18 19 20 21 22 23 24
10 m
C. elegans haplotypes:
HO1
HO2
HO3
HO4
HO5
HO7
C. briggsae
with C. virilis
no Caenorhabditis

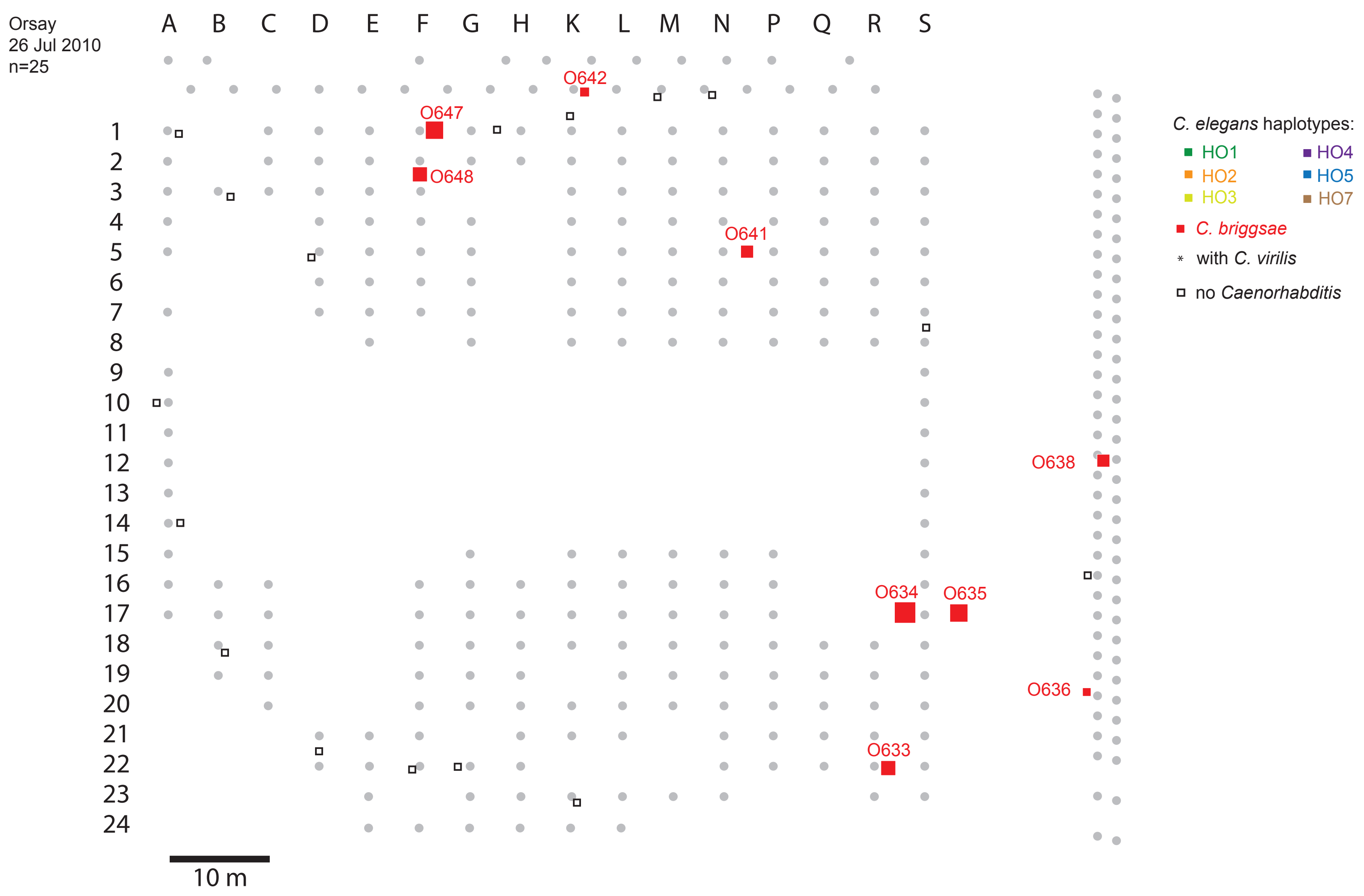
Orsay
26 Jul 2010
n=25
A B C D E F G H K L M N P Q R S
1 2 3 4 5 6 7 8 9 10 11 12 13 14 15 16 17 18 19 20 21 22 23 24
O642
O647
O648
O641
O638
O634
O635
O636
O633
10 m
C. elegans haplotypes:
HO1
HO2
HO3
HO4
HO5
HO7
C. briggsae
with C. virilis
no Caenorhabditis

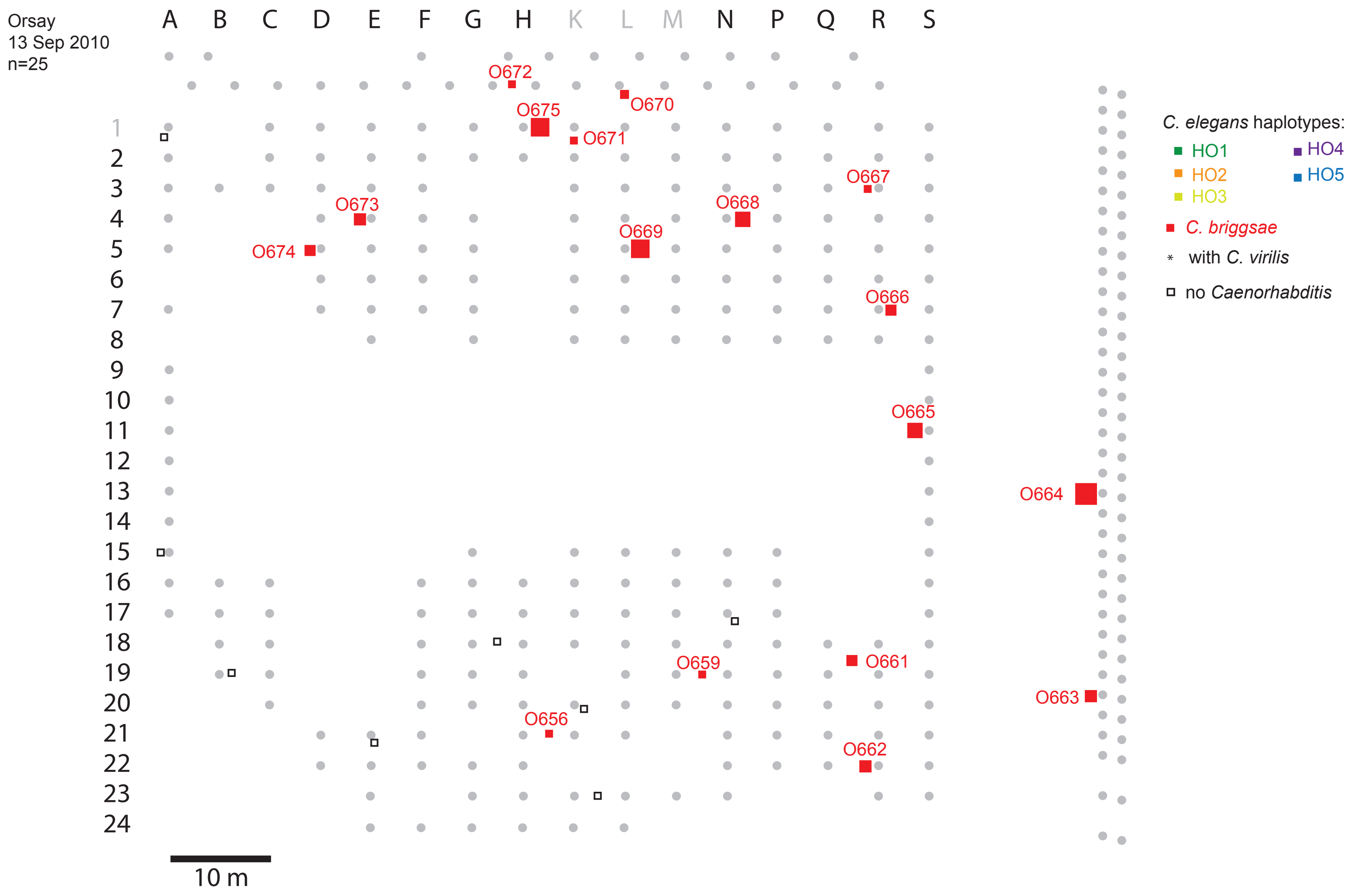

Orsay
13 Sep 2010
n=25
A B C D E F G H K L M N P Q R S
1 2 3 4 5 6 7 8 9 10 11 12 13 14 15 16 17 18 19 20 21 22 23 24
O672
O675
O670
O671
O667
O673
O668
O669
O674
O666
O665
O664
O659
O661
O663
O656
O662
10 m
C. elegans haplotypes:
HO1
HO2
HO3
HO4
HO5
C. briggsae
with C. virilis
no Caenorhabditis

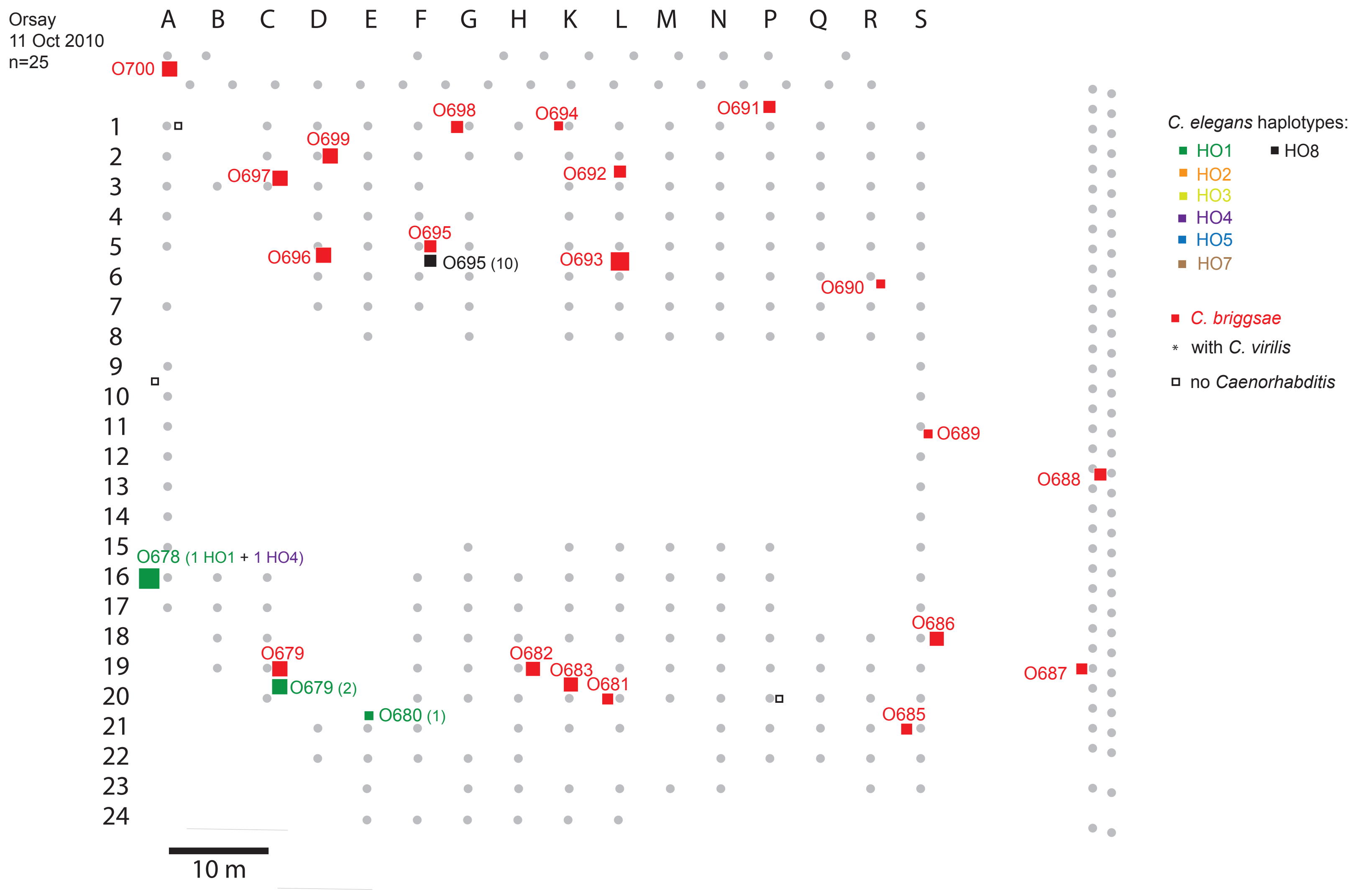
Orsay
11 Oct 2010
n=25
A B C D E F G H K L M N P Q R S
1 2 3 4 5 6 7 8 9 10 11 12 13 14 15 16 17 18 19 20 21 22 23 24
O700
O698
O694
O691
O699
O697
O692
O695
O696
O695 (10)
O693
O690
O689
O688
O678 (1 HO1 + 1 HO4)
O686
O679
O682
O683
O681
O687
O679 (2)
O680 (1)
O685
10 m
C. elegans haplotypes:
HO1
HO8
HO2
HO3
HO4
HO5
HO7
C. briggsae
with C. virilis
no Caenorhabditis

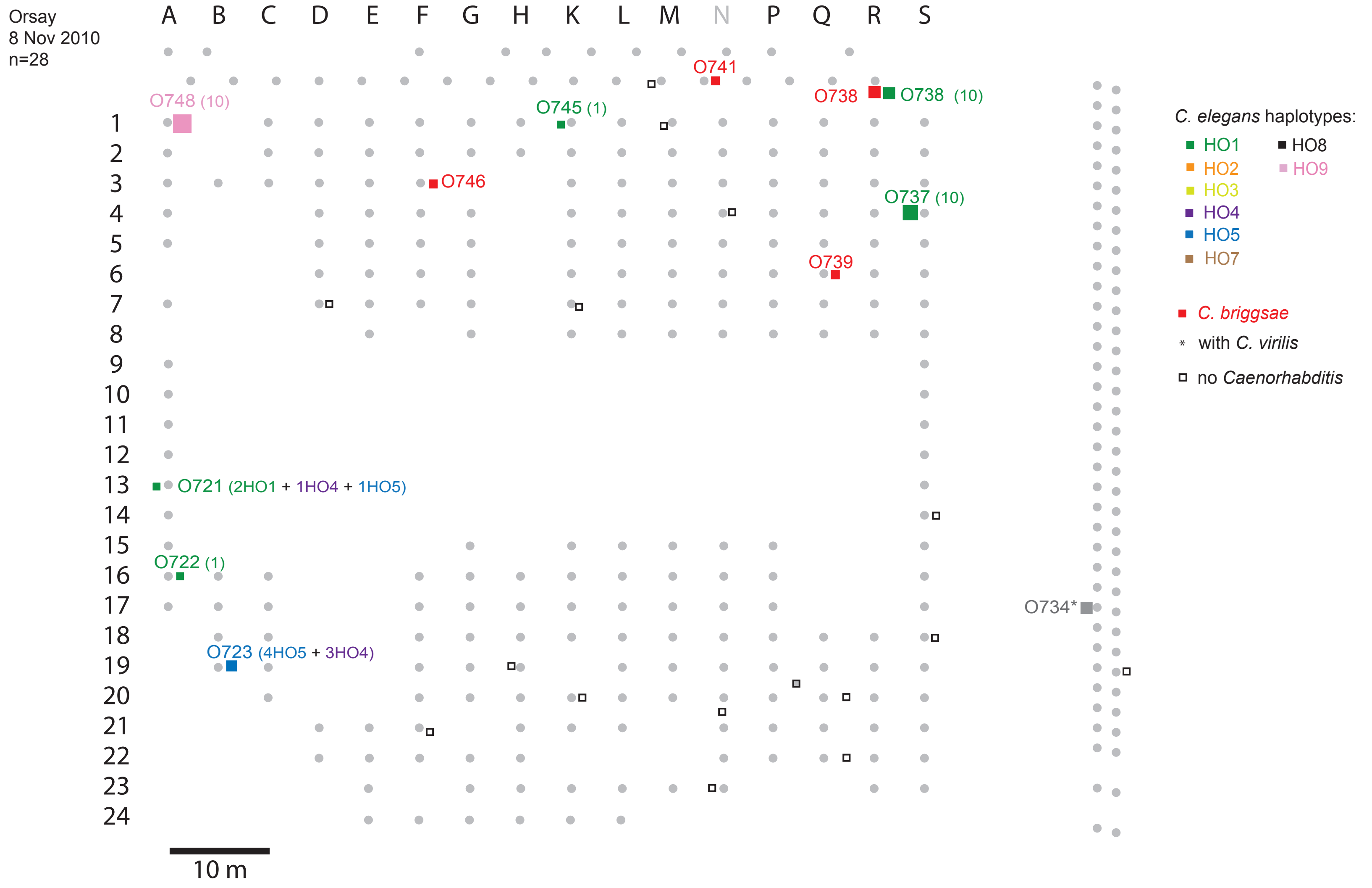
Orsay
8 Nov 2010
n=28
A B C D E F G H K L M N P Q R S
1 2 3 4 5 6 7 8 9 10 11 12 13 14 15 16 17 18 19 20 21 22 23 24
O748 (10)
O745 (1)
O741
O738
O738 (10)
O746
O737 (10)
O739
O721 (2HO1 + 1HO4 + 1HO5)
O722 (1)
O723 (4HO5 + 3HO4)
O734*
10 m
C. elegans haplotypes:
HO1
HO2
HO3
HO4
HO5
HO7
HO8
HO9
C. briggsae
* with C. virilis
no Caenorhabditis

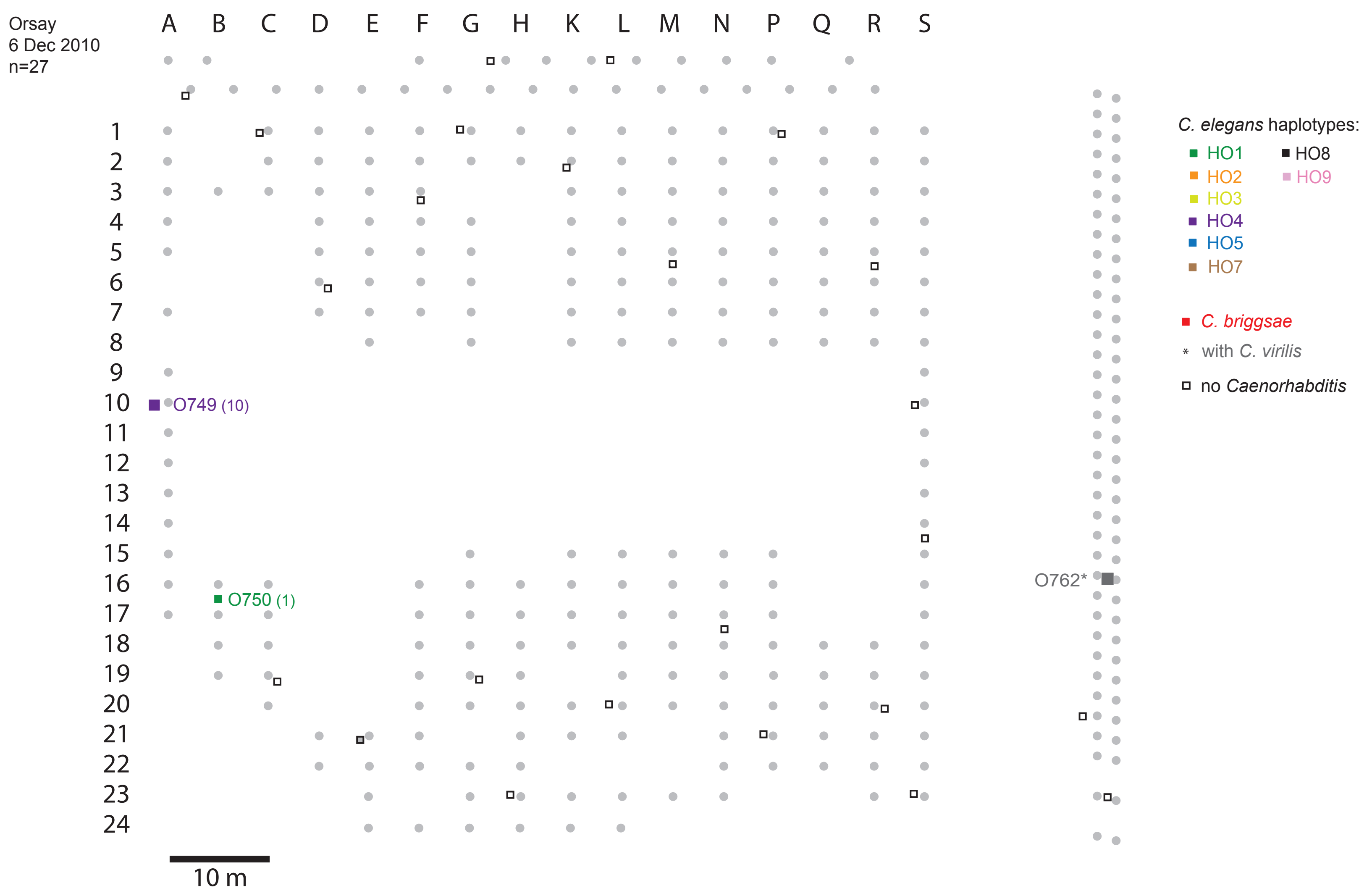
Orsay
6 Dec 2010
n=27
A B C D E F G H K L M N P Q R S
1 2 3 4 5 6 7 8 9 10 11 12 13 14 15 16 17 18 19 20 21 22 23 24
O749 (10)
O750 (1)
O762*
10 m
C. elegans haplotypes:
HO1
HO2
HO3
HO4
HO5
HO7
HO8
HO9
C. briggsae
* with C. virilis
no Caenorhabditis

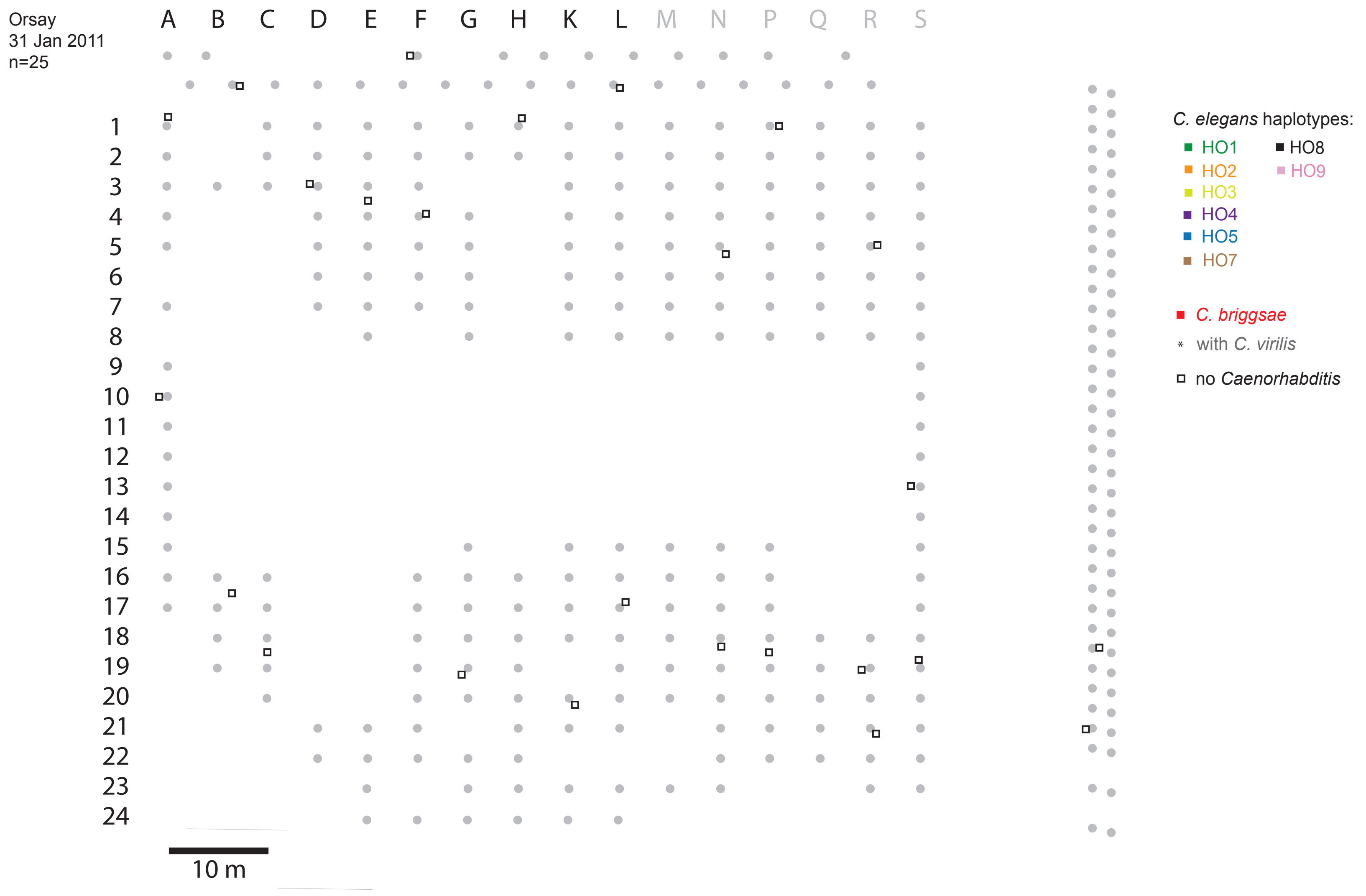
Orsay
31 Jan 2011
n=25
A B C D E F G H K L M N P Q R S
1
2
3
4
5
6
7
8
9
10
11
12
13
14
15
16
17
18
19
20
21
22
23
24
10 m
C. elegans haplotypes:
HO1
HO2
HO3
HO4
HO5
HO7
HO8
HO9
C. briggsae
* with C. virilis
no Caenorhabditis

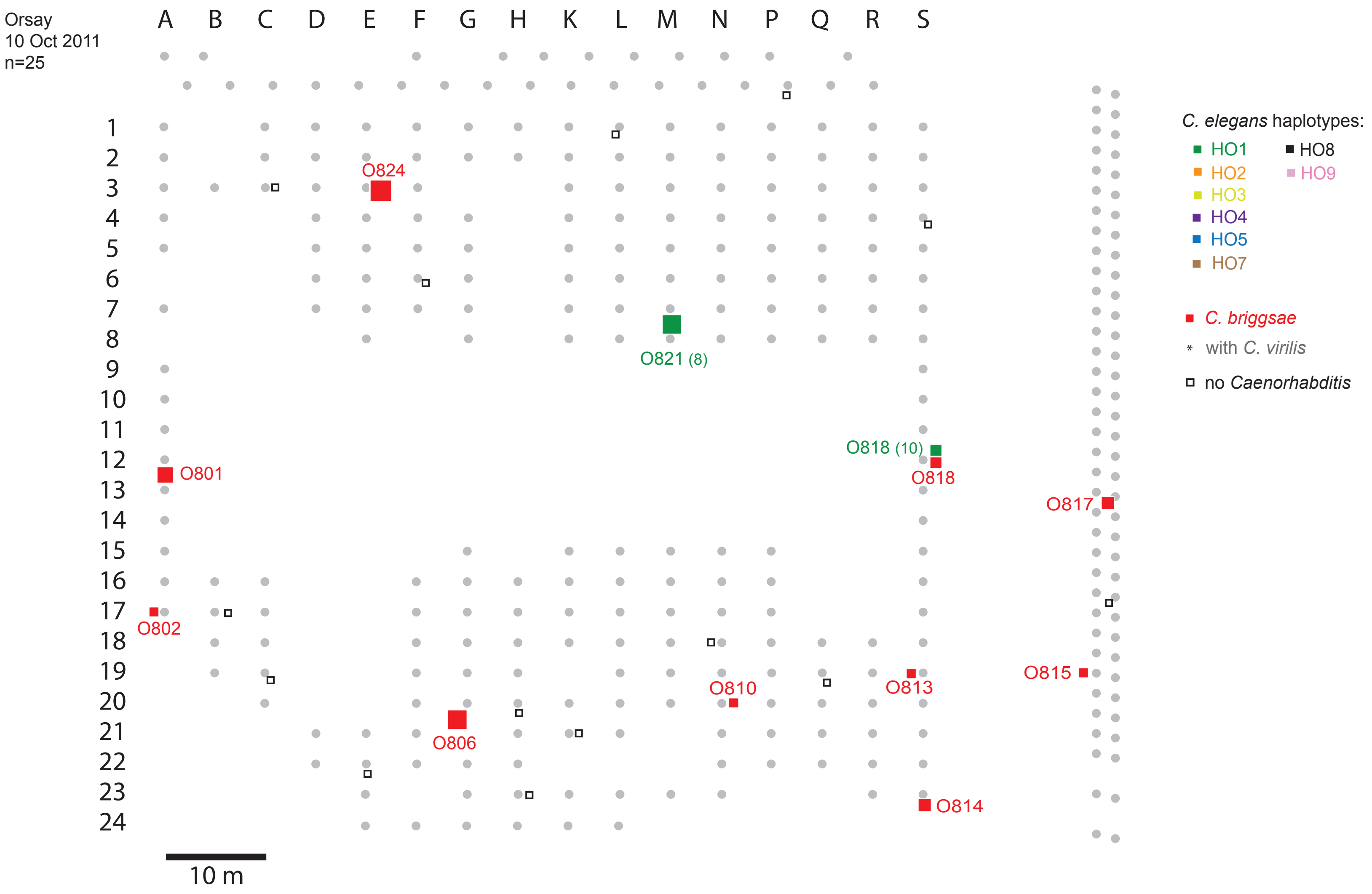

Orsay
10 Oct 2011
n=25
A B C D E F G H K L M N P Q R S
1 2 3 4 5 6 7 8 9 10 11 12 13 14 15 16 17 18 19 20 21 22 23 24
O824
O821 (8)
O818 (10)
O818
O801
O817
O802
O810
O813
O815
O806
O814
10 m
C. elegans haplotypes:
HO1
HO2
HO3
HO4
HO5
HO7
HO8
HO9
C. briggsae
with C. virilis
no Caenorhabditis

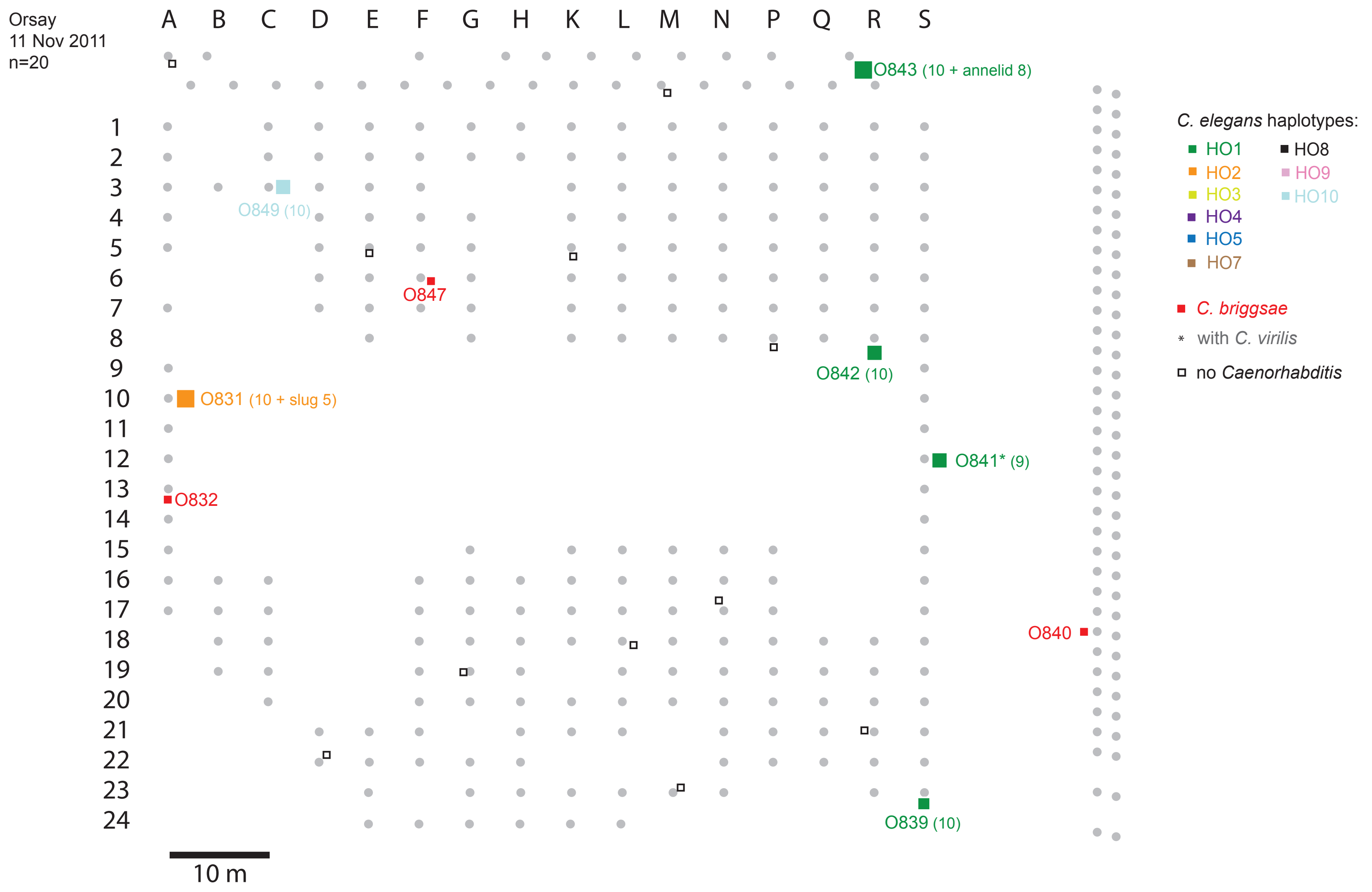
Orsay
11 Nov 2011
n=20
A B C D E F G H K L M N P Q R S
1 2 3 4 5 6 7 8 9 10 11 12 13 14 15 16 17 18 19 20 21 22 23 24
O843 (10 + annelid 8)
O849 (10)
O847
O842 (10)
O831 (10 + slug 5)
O841* (9)
O832
O840
O839 (10)
10 m
C. elegans haplotypes:
HO1
HO2
HO3
HO4
HO5
HO7
HO8
HO9
HO10
C. briggsae
* with C. virilis
no Caenorhabditis

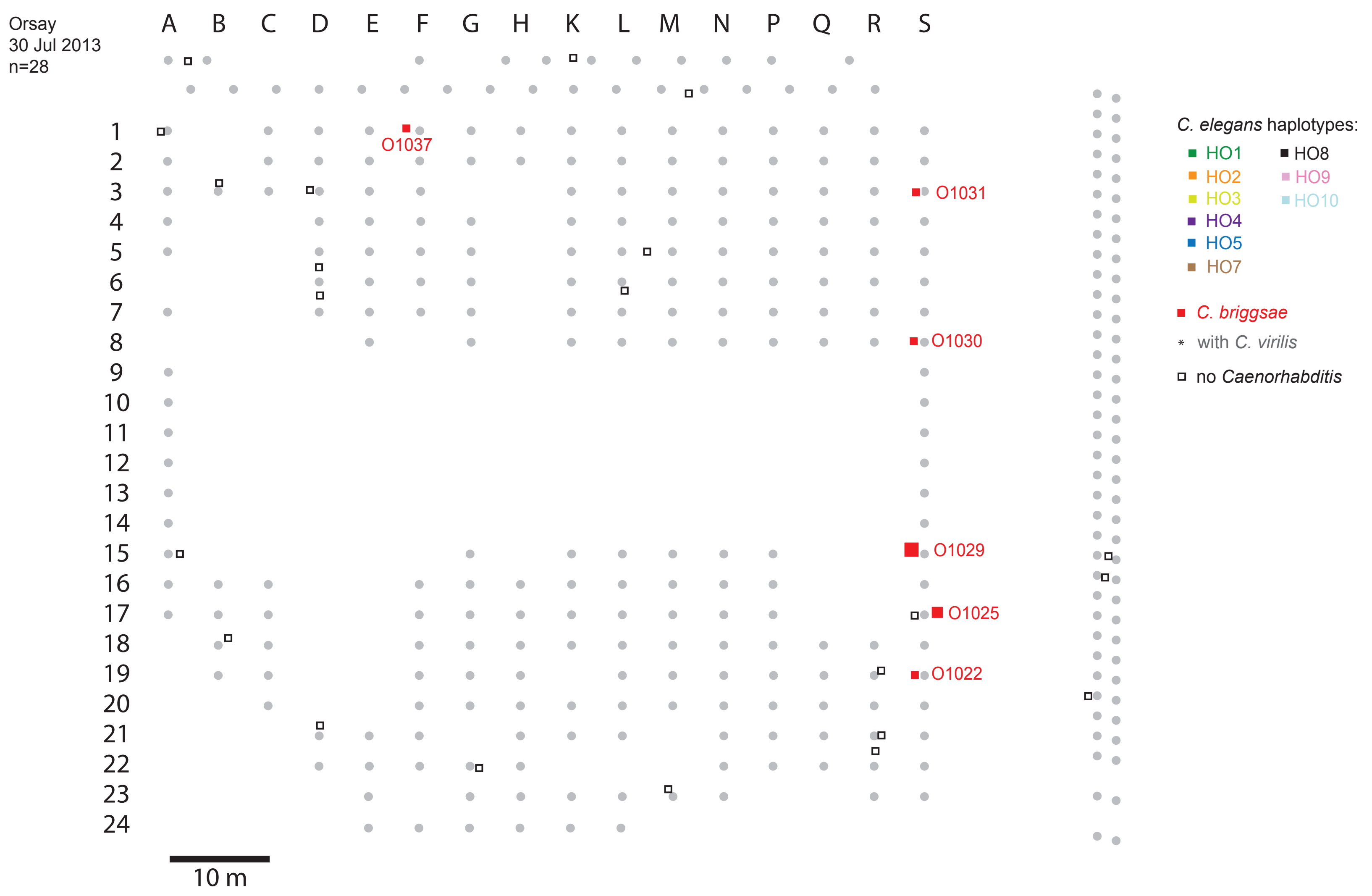

Orsay
30 Jul 2013
n=28
A B C D E F G H K L M N P Q R S
1 2 3 4 5 6 7 8 9 10 11 12 13 14 15 16 17 18 19 20 21 22 23 24
O1037
O1031
O1030
O1029
O1025
O1022
10 m
C. elegans haplotypes:
HO1
HO2
HO3
HO4
HO5
HO7
HO8
HO9
HO10
C. briggsae
with C. virilis
no Caenorhabditis

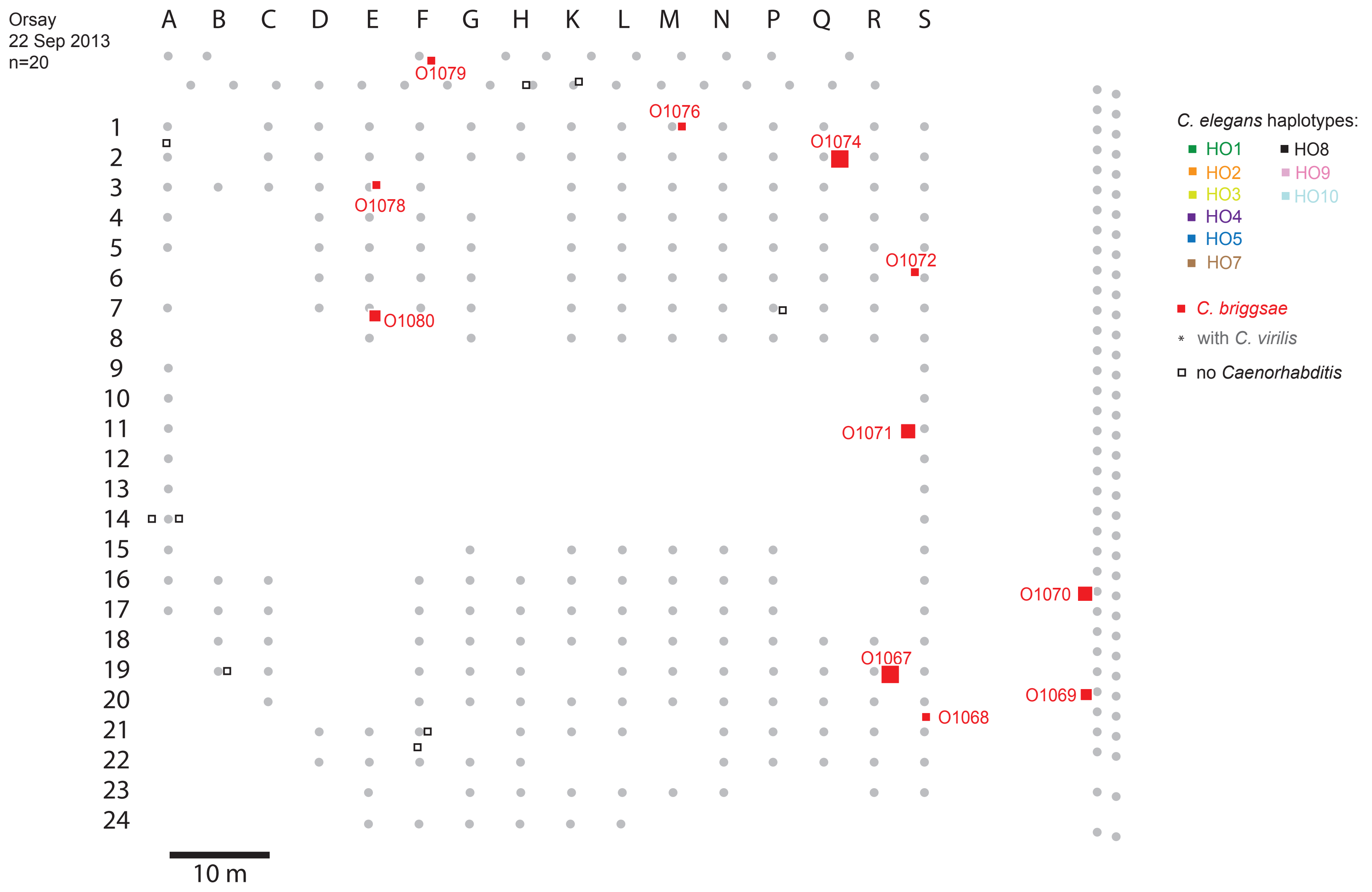
Orsay
22 Sep 2013
n=20
A B C D E F G H K L M N P Q R S
1 2 3 4 5 6 7 8 9 10 11 12 13 14 15 16 17 18 19 20 21 22 23 24
O1079
O1076
O1074
O1078
O1072
O1080
O1071
O1070
O1067
O1069
O1068
10 m
C. elegans haplotypes:
HO1
HO2
HO3
HO4
HO5
HO7
HO8
HO9
HO10
C. briggsae
with C. virilis
no Caenorhabditis

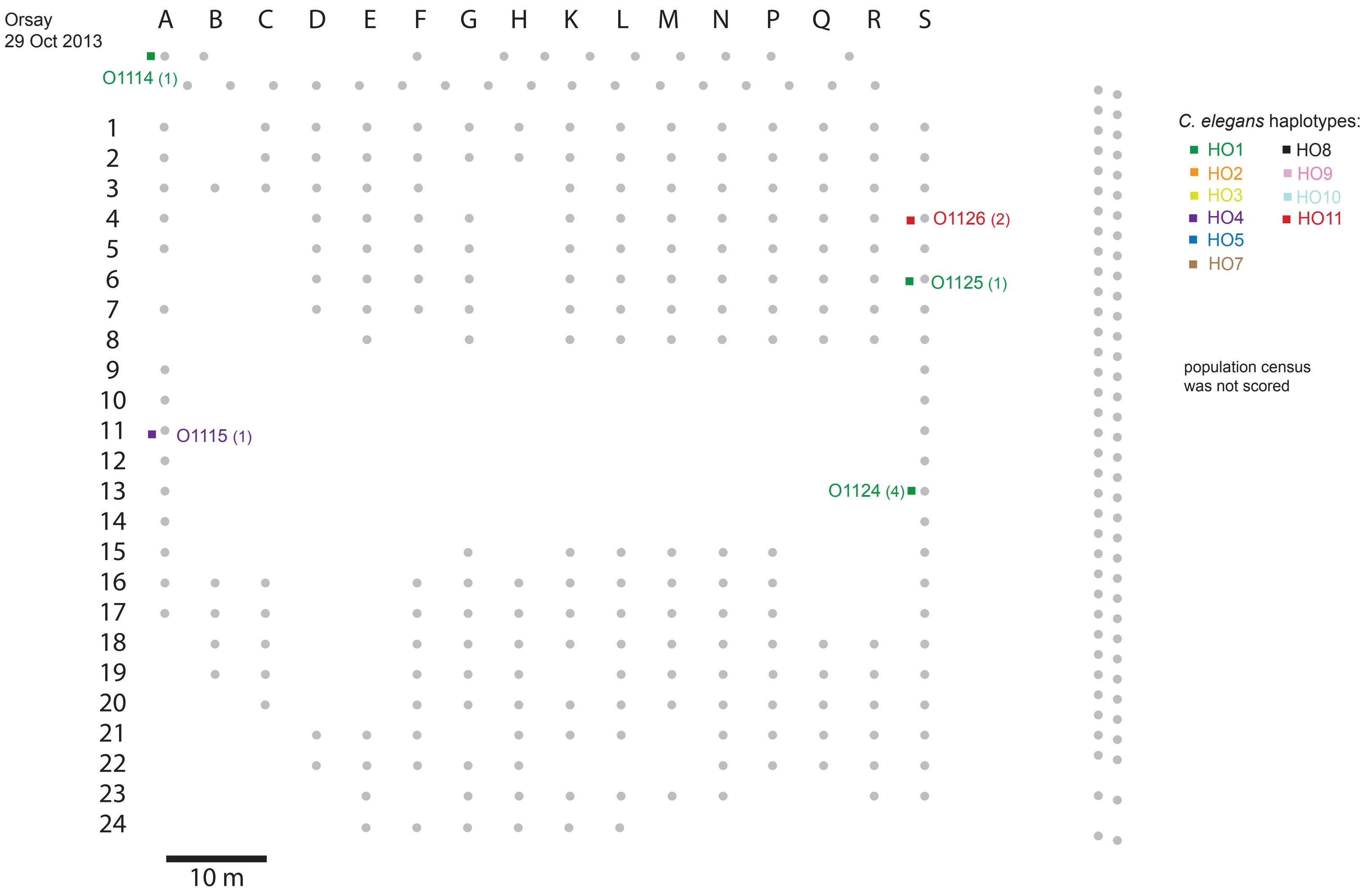
Orsay
29 Oct 2013
A B C D E F G H K L M N P Q R S
1 2 3 4 5 6 7 8 9 10 11 12 13 14 15 16 17 18 19 20 21 22 23 24
O1114 (1)
O1126 (2)
O1125 (1)
O1115 (1)
O1124 (4)
10 m
C. elegans haplotypes:
HO1
HO2
HO3
HO4
HO5
HO7
HO8
HO9
HO10
HO11
population census
was not scored

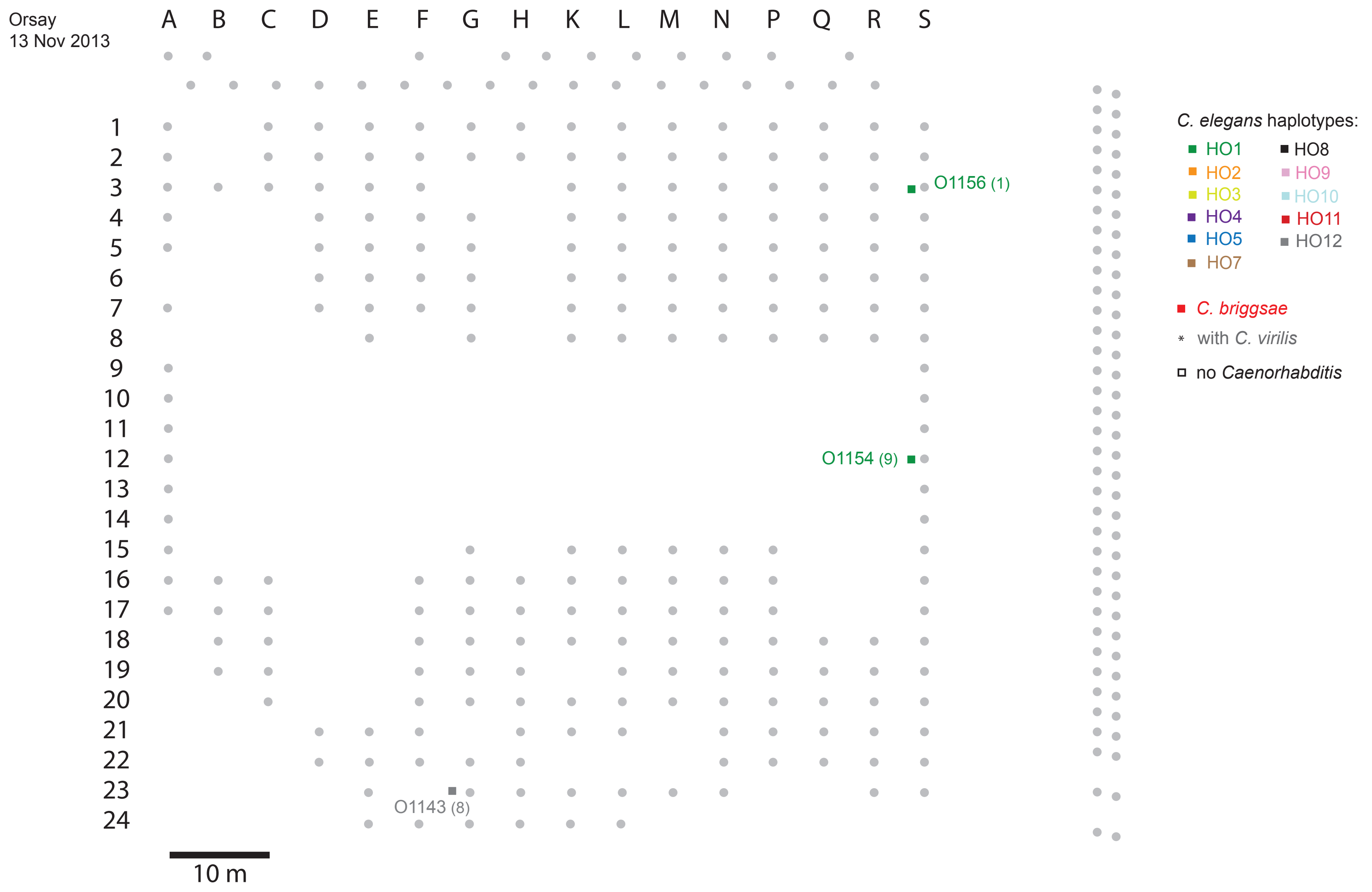
Orsay
13 Nov 2013
A B C D E F G H K L M N P Q R S
1
2
3
4
5
6
7
8
9
10
11
12
13
14
15
16
17
18
19
20
21
22
23
24
O1156 (1)
O1154 (9)
O1143 (8)
10 m
C. elegans haplotypes:
HO1
HO2
HO3
HO4
HO5
HO7
HO8
HO9
HO10
HO11
HO12
C. briggsae
with C. virilis
no Caenorhabditis

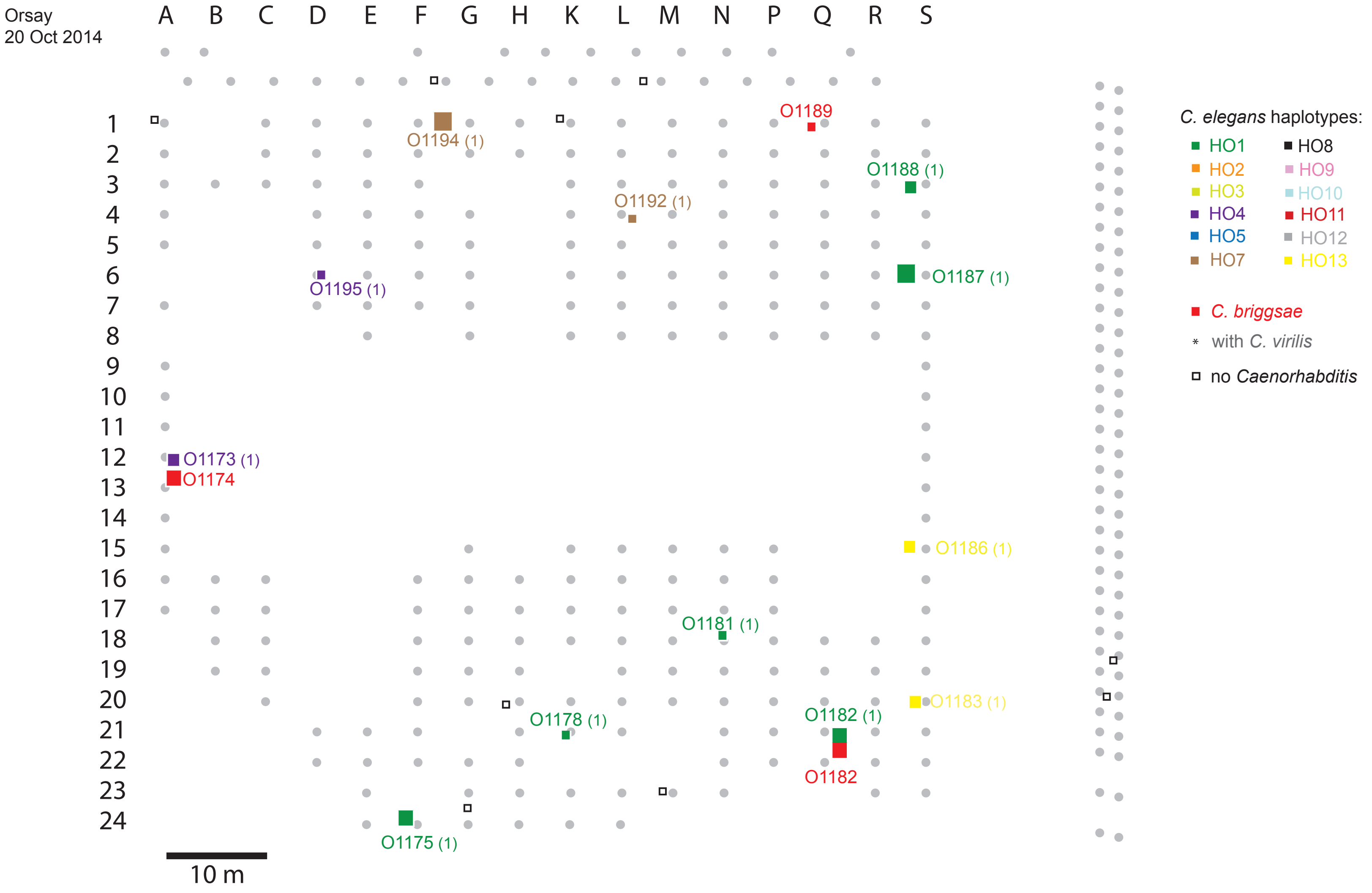
Orsay
20 Oct 2014
A B C D E F G H K L M N P Q R S
1
2
3
4
5
6
7
8
9
10
11
12
13
14
15
16
17
18
19
20
21
22
23
24
O1189
O1194 (1)
O1188 (1)
O1192 (1)
O1195 (1)
O1187 (1)
O1173 (1)
O1174
O1186 (1)
O1181 (1)
O1183 (1)
O1182 (1)
O1178 (1)
O1182
O1175 (1)
10 m
C. elegans haplotypes:
HO1
HO2
HO3
HO4
HO5
HO7
HO8
HO9
HO10
HO11
HO12
HO13
C. briggsae
with C. virilis
no Caenorhabditis
