# Supplementary material for: The Local Coexistence Pattern of Selfing Genotypes in Caenorhabditis elegans Natural Metapopulations
Source: Genetics. 2017 Dec 12;208(2):807–21. doi: 10.1534/genetics.117.300564 (PMC5788539; doi:10.1534/genetics.117.300564)
Supplement: Supplementary file 2 [file 807FigureS7.pdf]

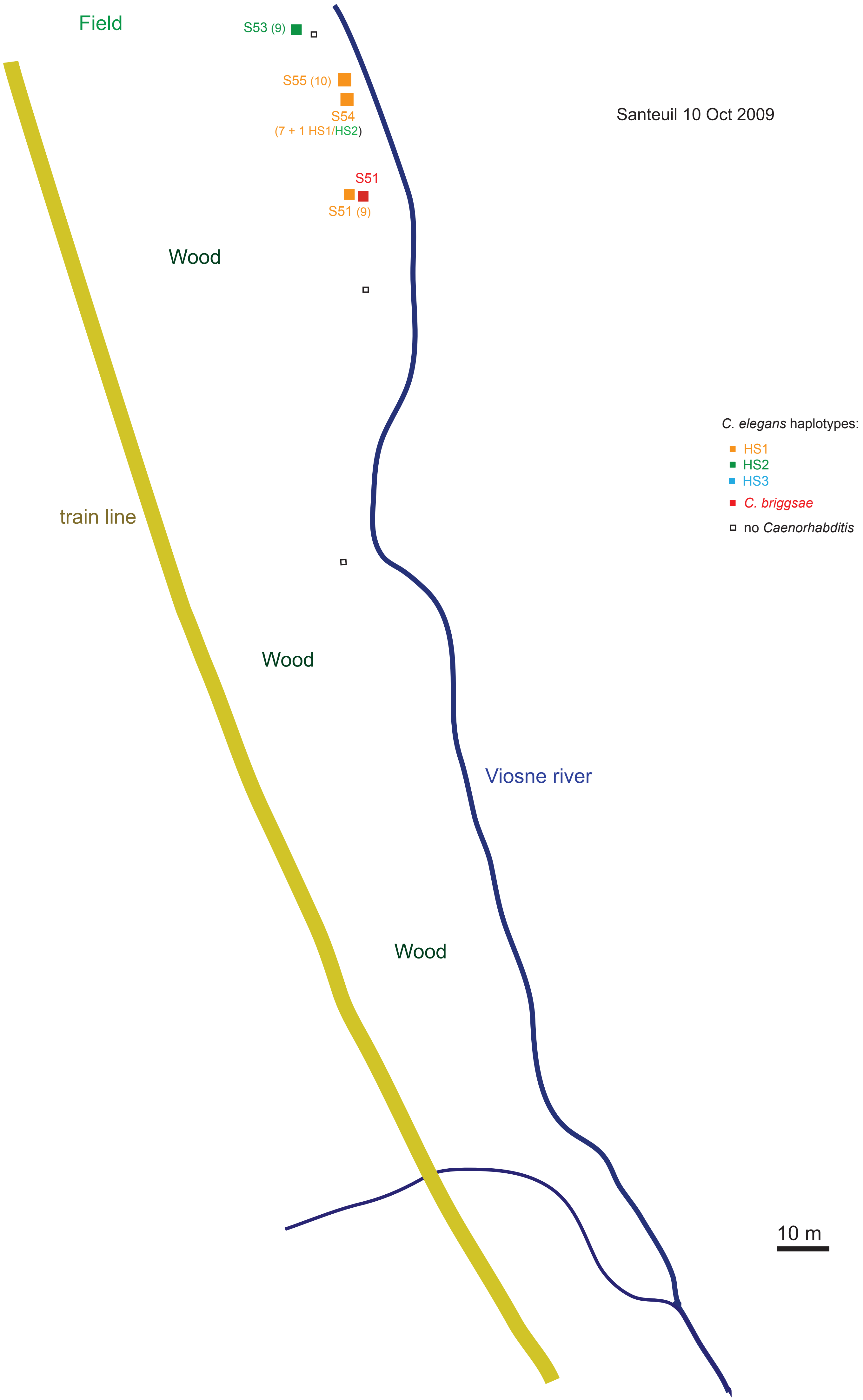

Field
S53 (9)
S55 (10)
S54
(7 + 1 HS1/HS2)
S51
S51 (9)
Santeuil 10 Oct 2009
Wood
C. elegans haplotypes:
HS1
HS2
HS3
C. briggsae
no Caenorhabditis
train line
Wood
Viosne river
Wood
10 m

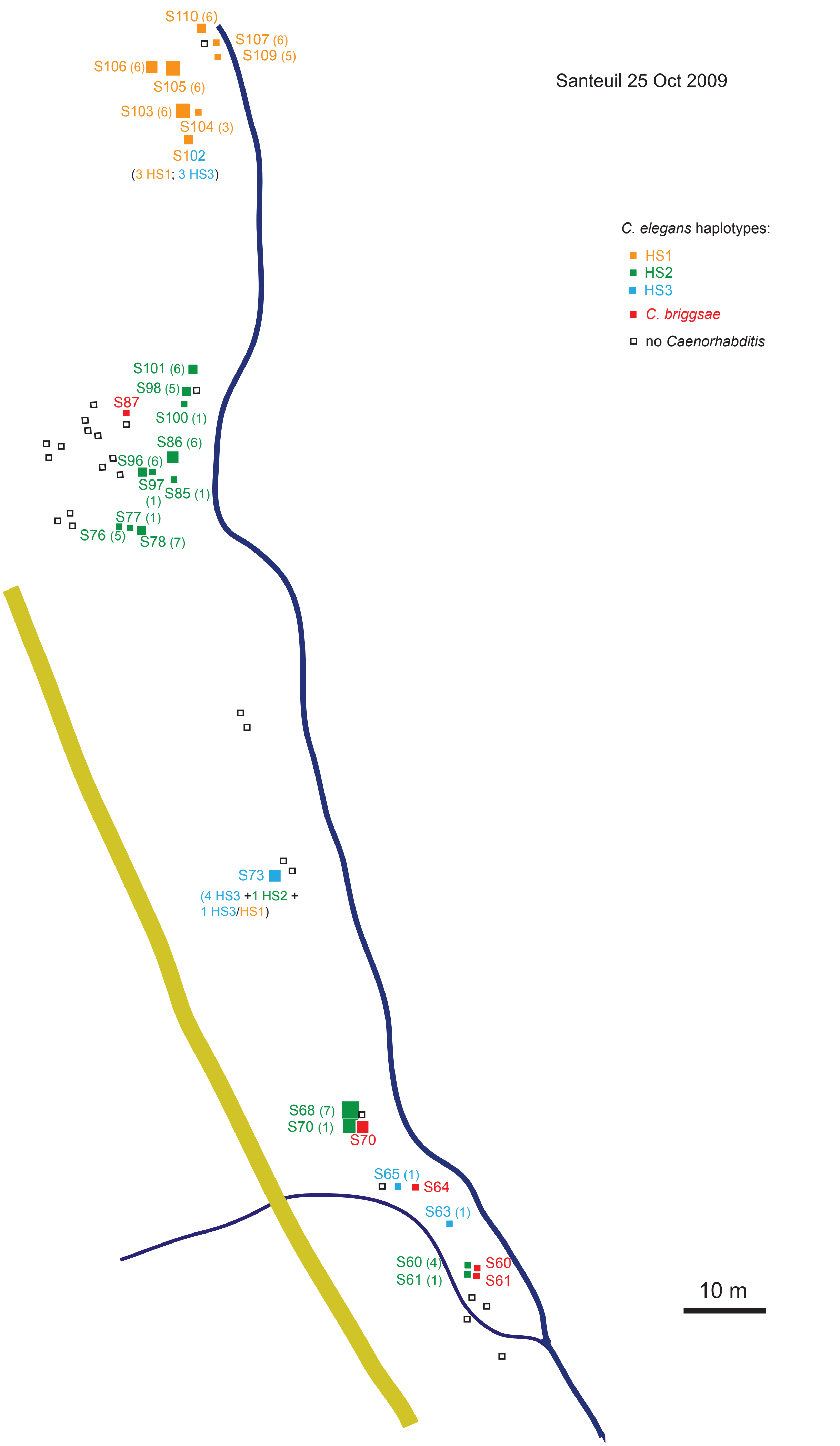
Santeuil 25 Oct 2009
C. elegans haplotypes:
HS1
HS2
HS3
C. briggsae
no Caenorhabditis
S110 (6)
S107 (6)
S109 (5)
S106 (6)
S105 (6)
S103 (6)
S104 (3)
S102
(3 HS1; 3 HS3)
S101 (6)
S98 (5)
S87
S100 (1)
S86 (6)
S96 (6)
S97
(1)
S85 (1)
S77 (1)
S76 (5)
S78 (7)
S73
(4 HS3 +1 HS2 +
1 HS3/HS1)
S68 (7)
S70 (1)
S70
S65 (1)
S64
S63 (1)
S60 (4)
S61 (1)
S60
S61
10 m

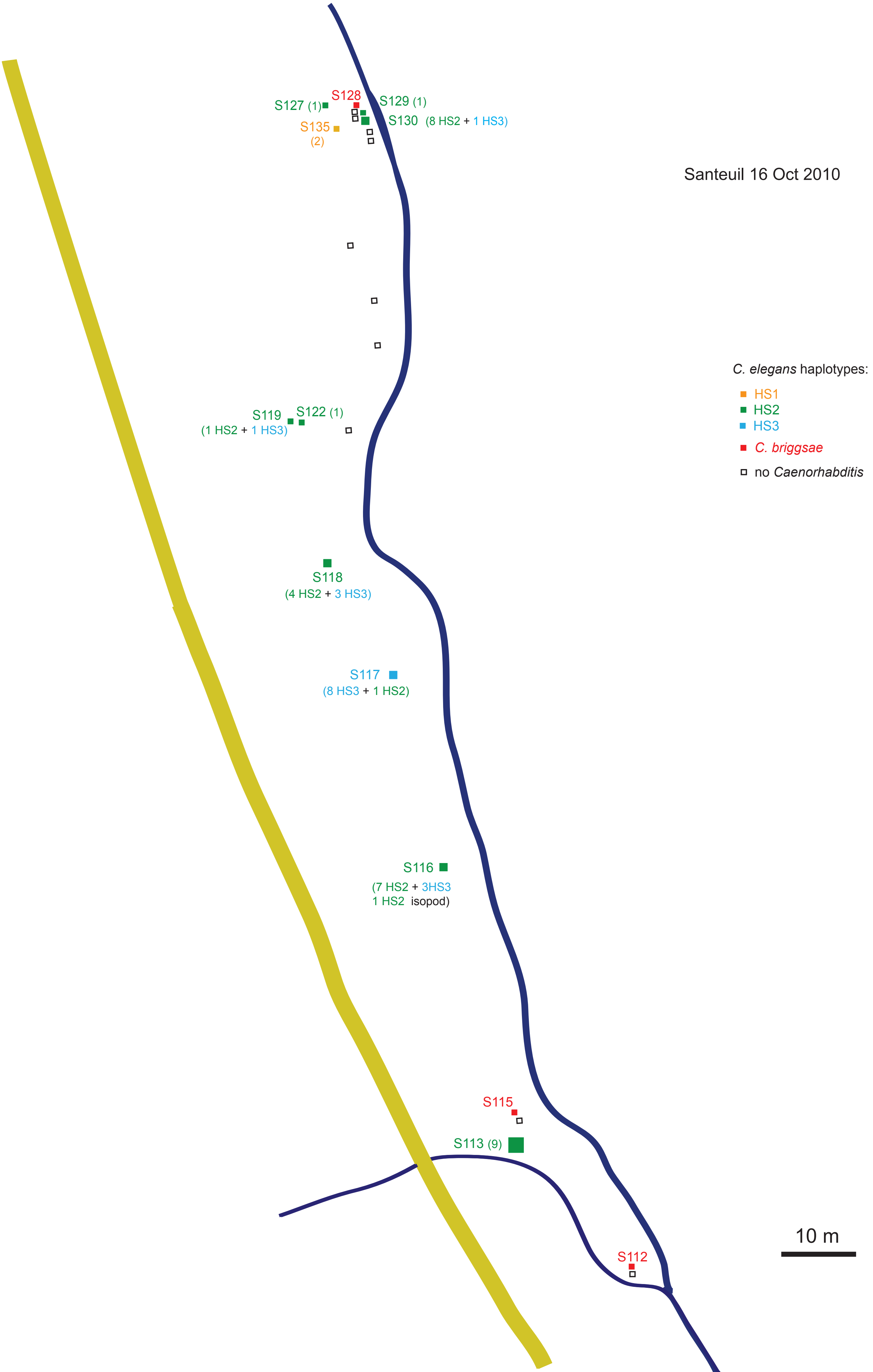

Santeuil 16 Oct 2010
C. elegans haplotypes:
HS1
HS2
HS3
C. briggsae
no Caenorhabditis
S128
S127 (1)
S129 (1)
S130 (8 HS2 + 1 HS3)
S135
(2)
S119
(1 HS2 + 1 HS3)
S122 (1)
S118
(4 HS2 + 3 HS3)
S117
(8 HS3 + 1 HS2)
S116
(7 HS2 + 3HS3
1 HS2 isopod)
S115
S113 (9)
S112
10 m

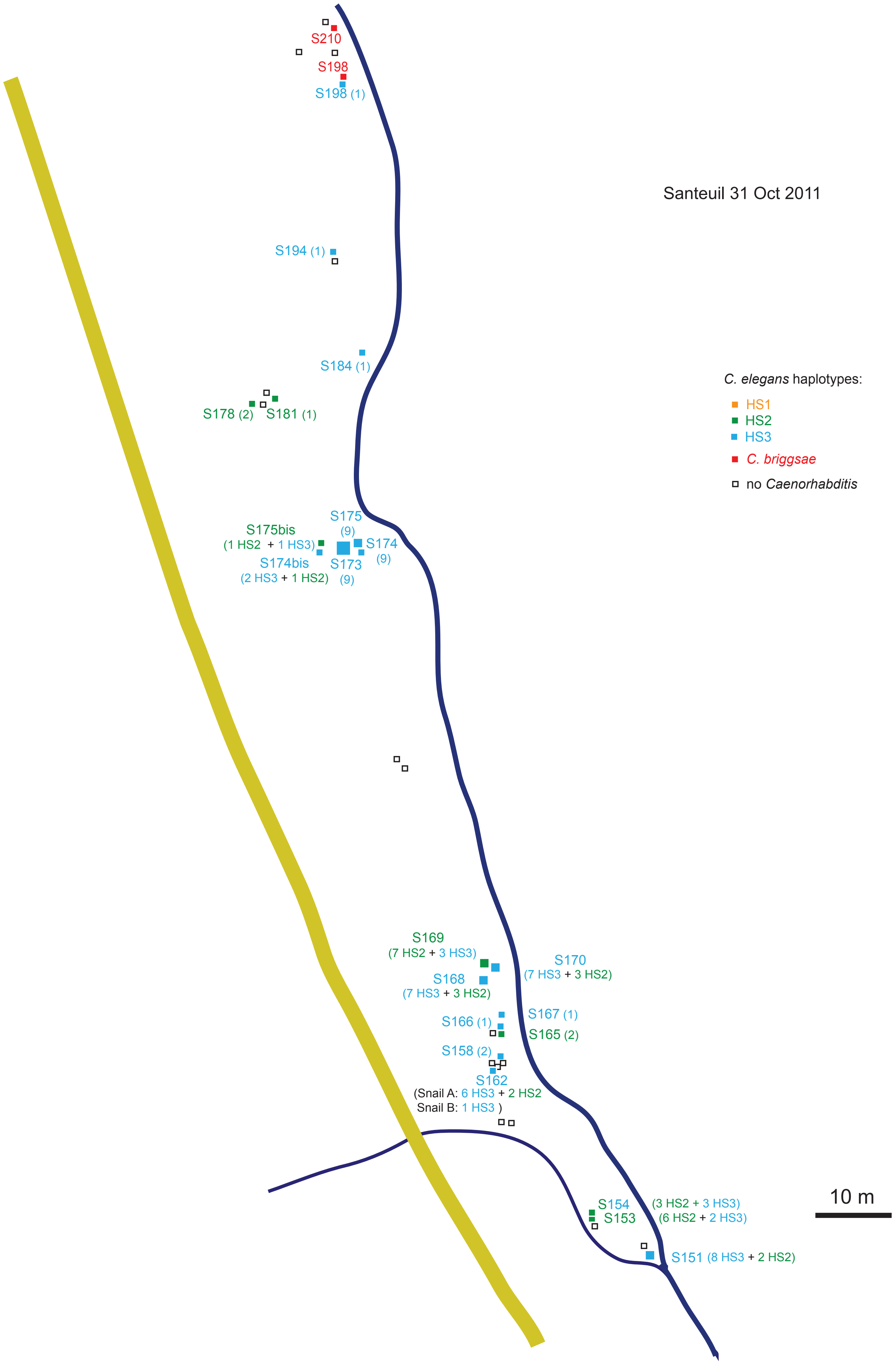

Santeuil 31 Oct 2011
C. elegans haplotypes:
HS1
HS2
HS3
C. briggsae
no Caenorhabditis
10 m
S210
S198
S198 (1)
S194 (1)
S184 (1)
S178 (2)
S181 (1)
S175
(9)
S175bis
(1 HS2 + 1 HS3)
S174
(9)
S174bis
(2 HS3 + 1 HS2)
S173
(9)
S169
(7 HS2 + 3 HS3)
S170
(7 HS3 + 3 HS2)
S168
(7 HS3 + 3 HS2)
S167 (1)
S166 (1)
S165 (2)
S158 (2)
S162
(Snail A: 6 HS3 + 2 HS2
Snail B: 1 HS3)
S154
S153
(3 HS2 + 3 HS3)
(6 HS2 + 2 HS3)
S151 (8 HS3 + 2 HS2)

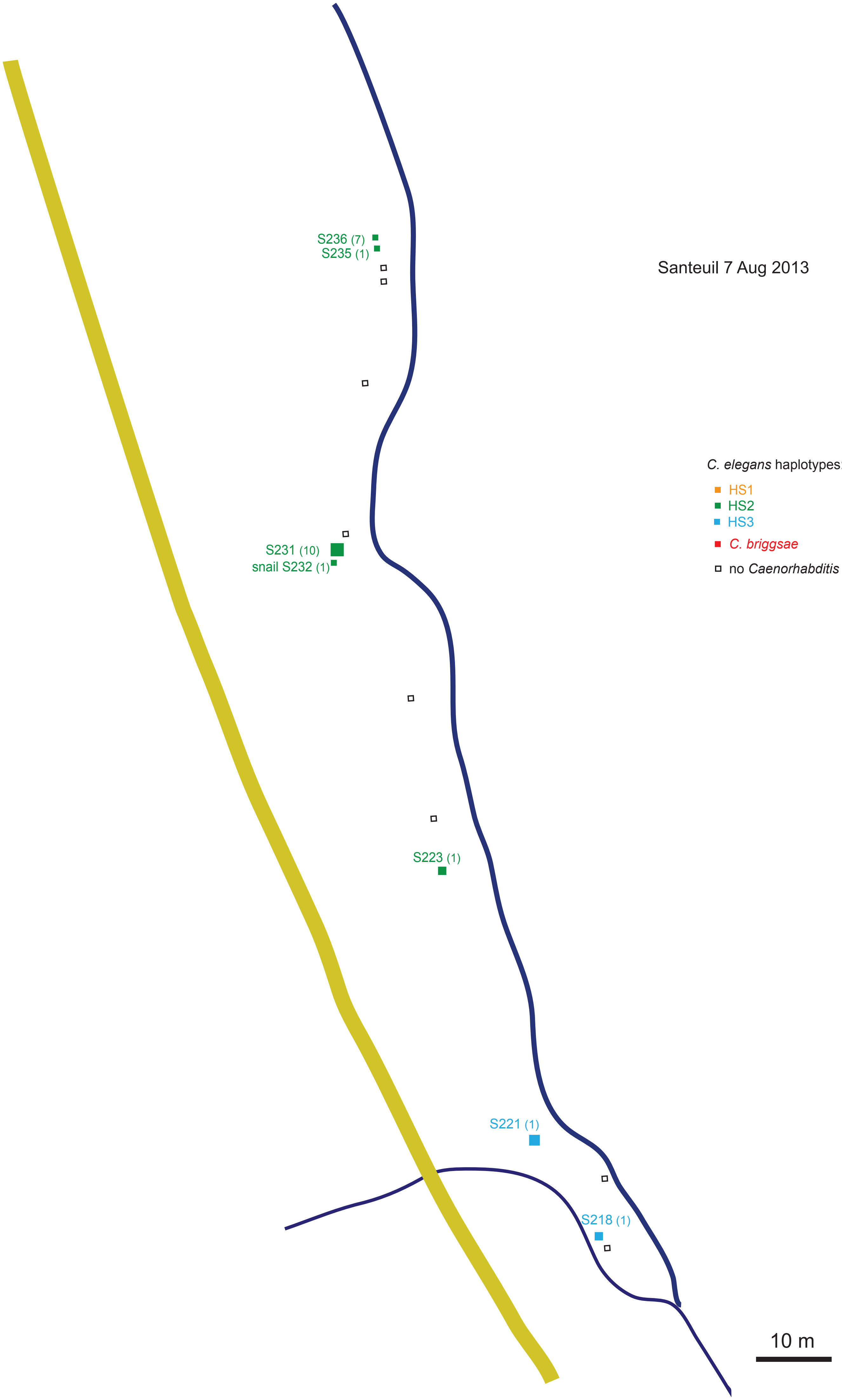
Santeuil 7 Aug 2013
C. elegans haplotypes:
HS1
HS2
HS3
C. briggsae
no Caenorhabditis
S236 (7)
S235 (1)
S231 (10)
snail S232 (1)
S223 (1)
S221 (1)
S218 (1)
10 m

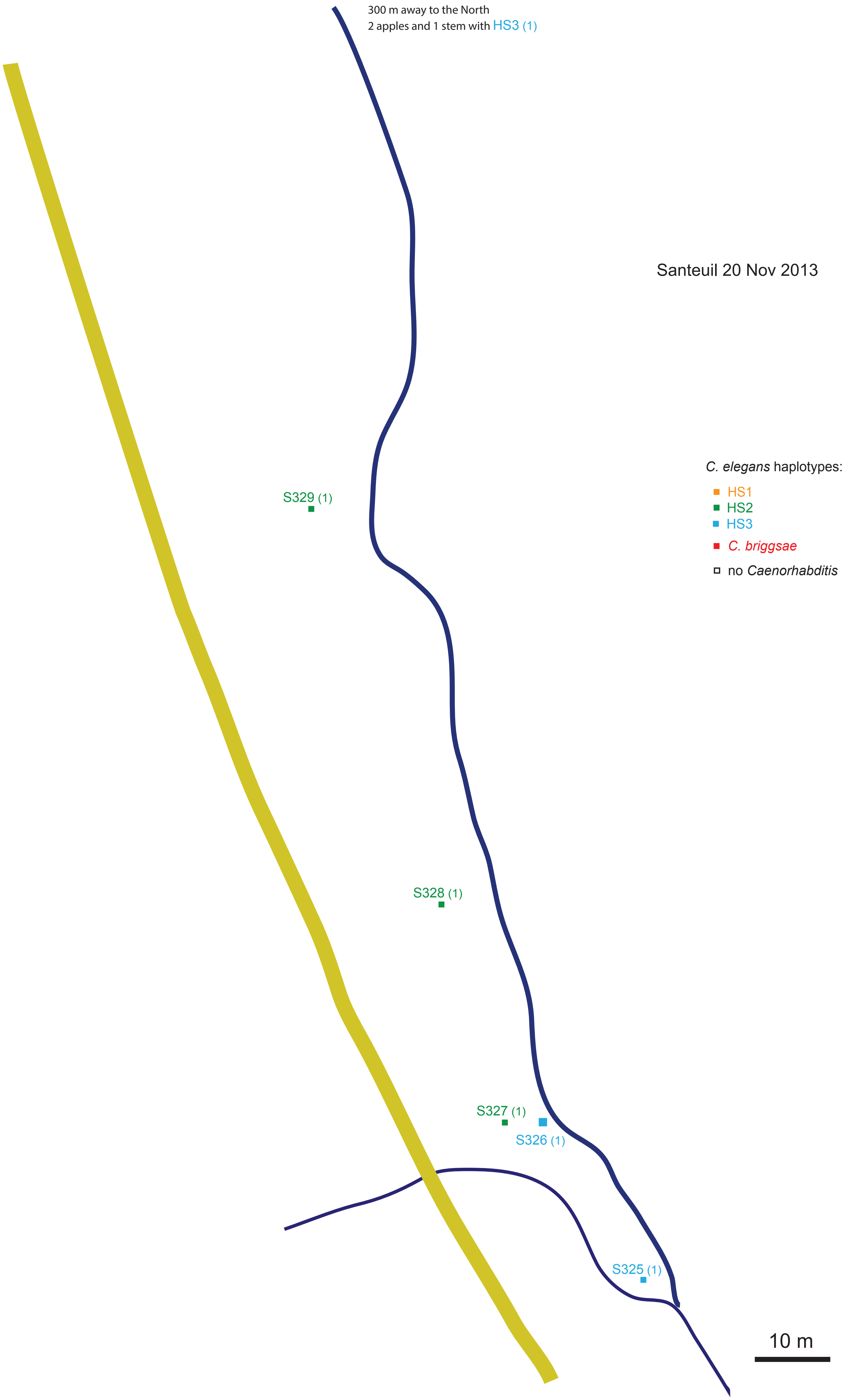
300 m away to the North
2 apples and 1 stem with HS3 (1)
Santeuil 20 Nov 2013
C. elegans haplotypes:
HS1
HS2
HS3
C. briggsae
no Caenorhabditis
S329 (1)
S328 (1)
S327 (1)
S326 (1)
S325 (1)
10 m

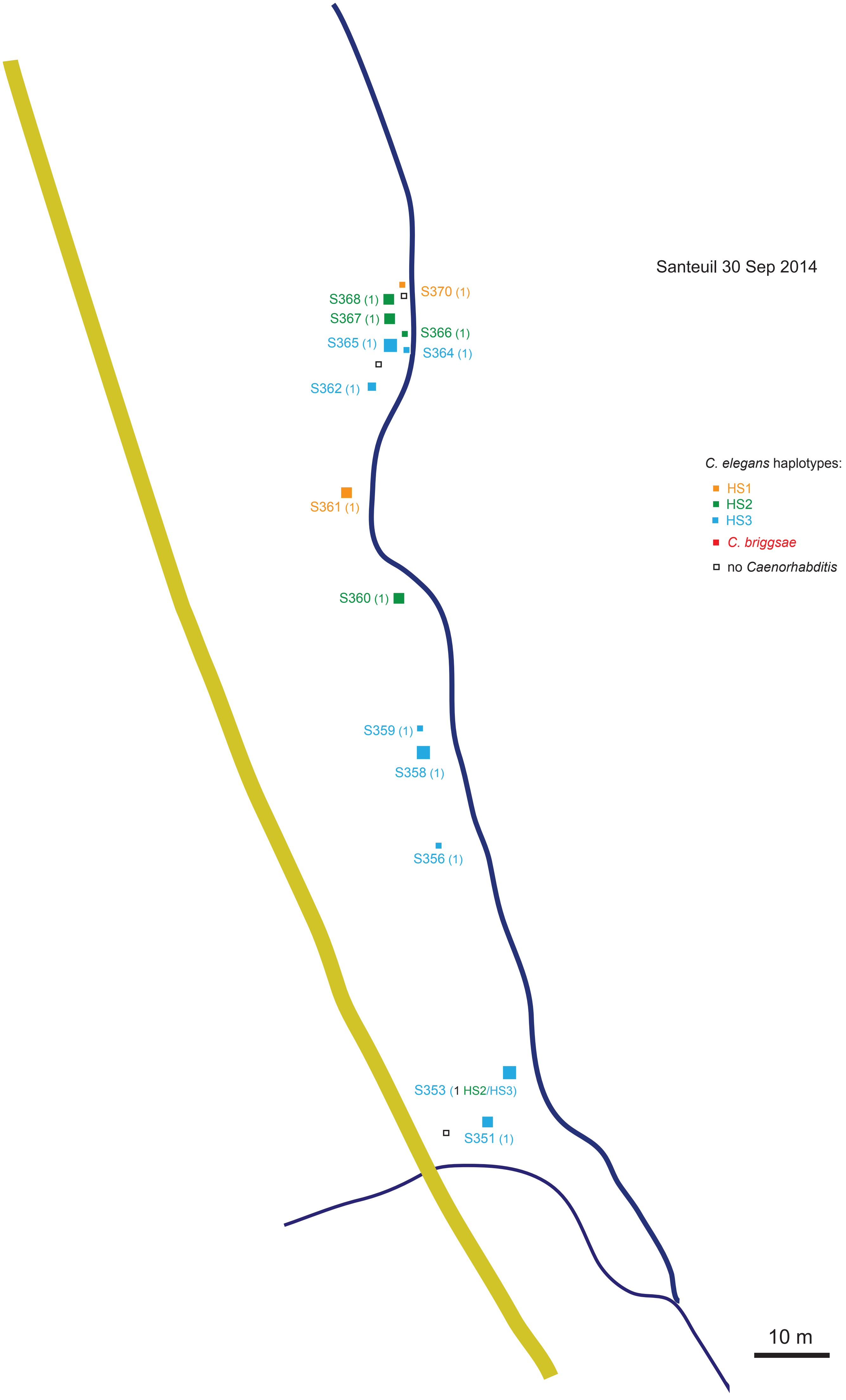

Santeuil 30 Sep 2014
S370 (1)
S368 (1)
S367 (1)
S366 (1)
S365 (1)
S364 (1)
S362 (1)
S361 (1)
S360 (1)
S359 (1)
S358 (1)
S356 (1)
S353 (1 HS2/HS3)
S351 (1)
C. elegans haplotypes:
HS1
HS2
HS3
C. briggsae
no Caenorhabditis
10 m
