# Supplementary figures and images for: The Local Coexistence Pattern of Selfing Genotypes in Caenorhabditis elegans Natural Metapopulations
Source: Genetics. 2017 Dec 12;208(2):807–21. doi: 10.1534/genetics.117.300564 (PMC5788539; doi:10.1534/genetics.117.300564)

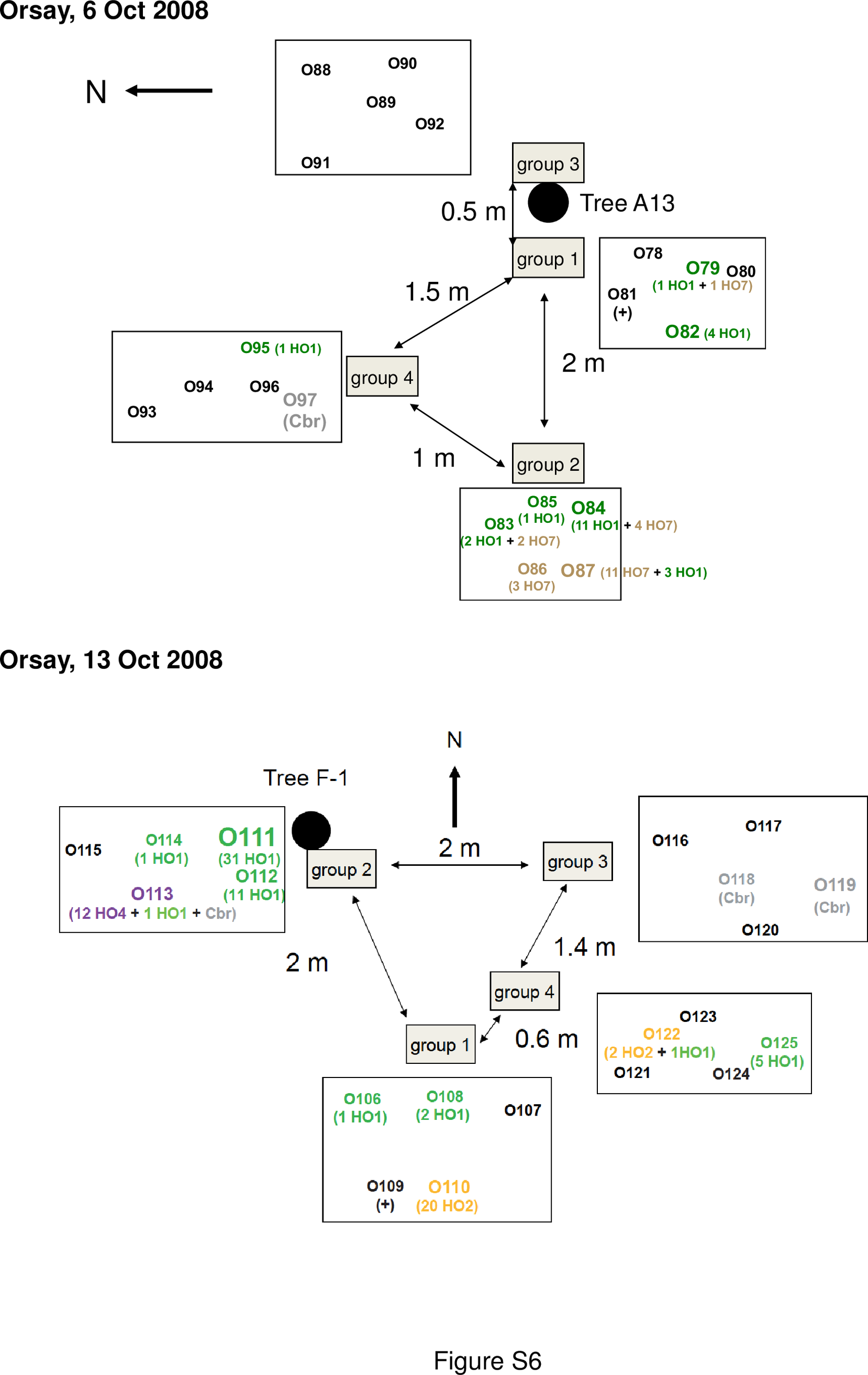

Supplement: Supplementary file 12 [file 807FileS1.zip › FigureS6.tiff]

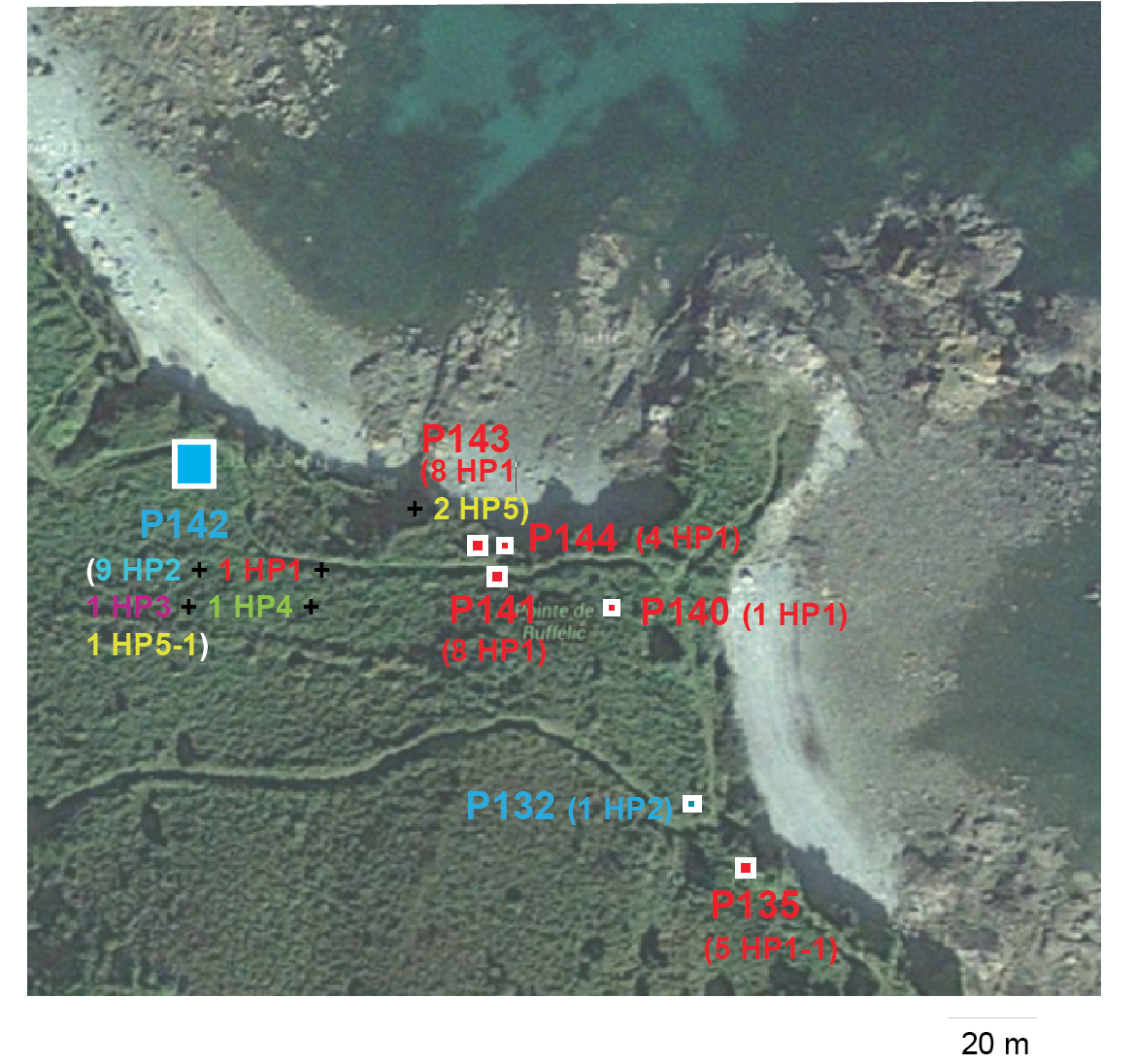

Supplement: Supplementary file 12 [file 807FileS1.zip › FigureS8.tiff]

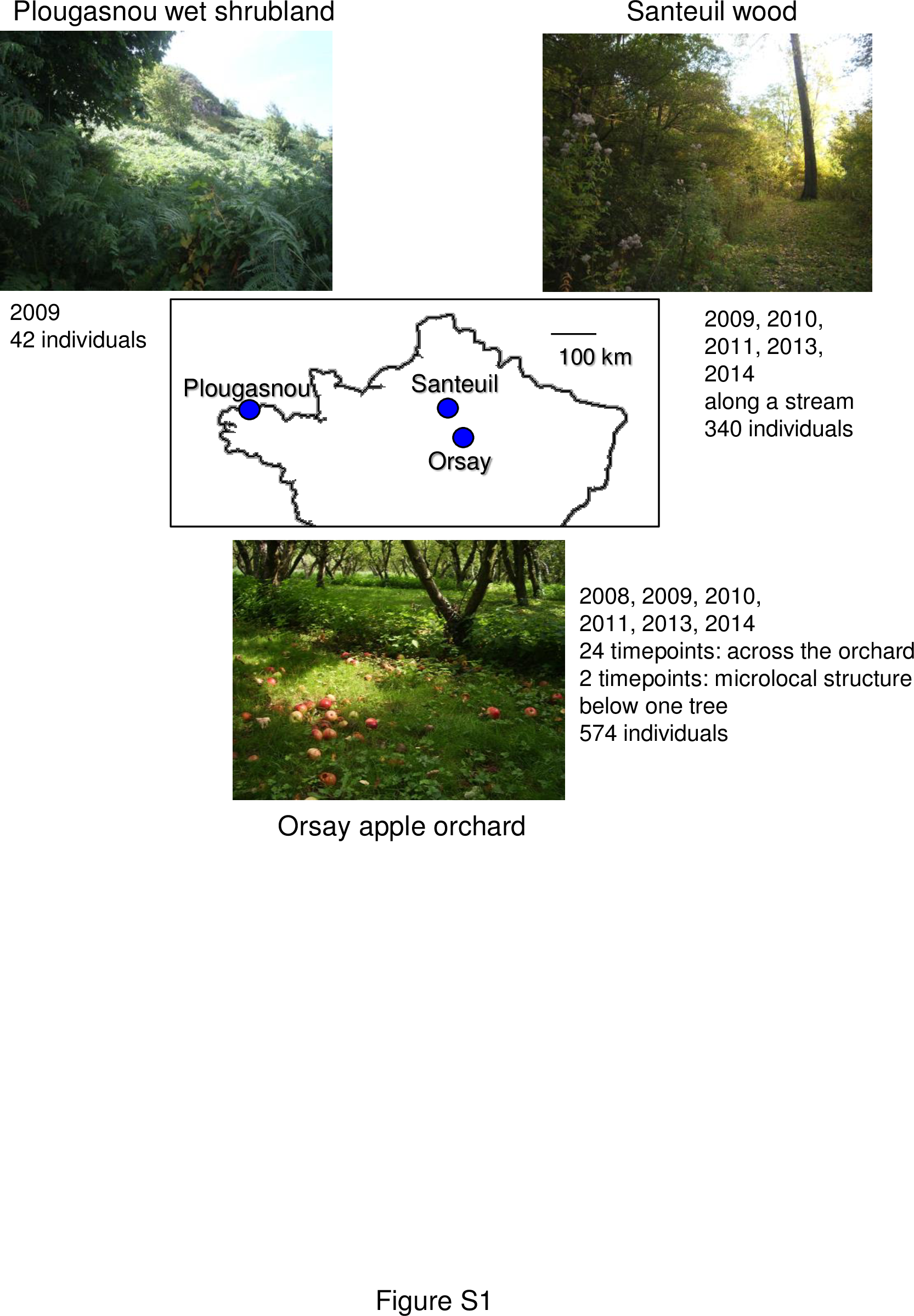

Supplement: Supplementary file 12 [file 807FileS1.zip › FigureS1.tiff]

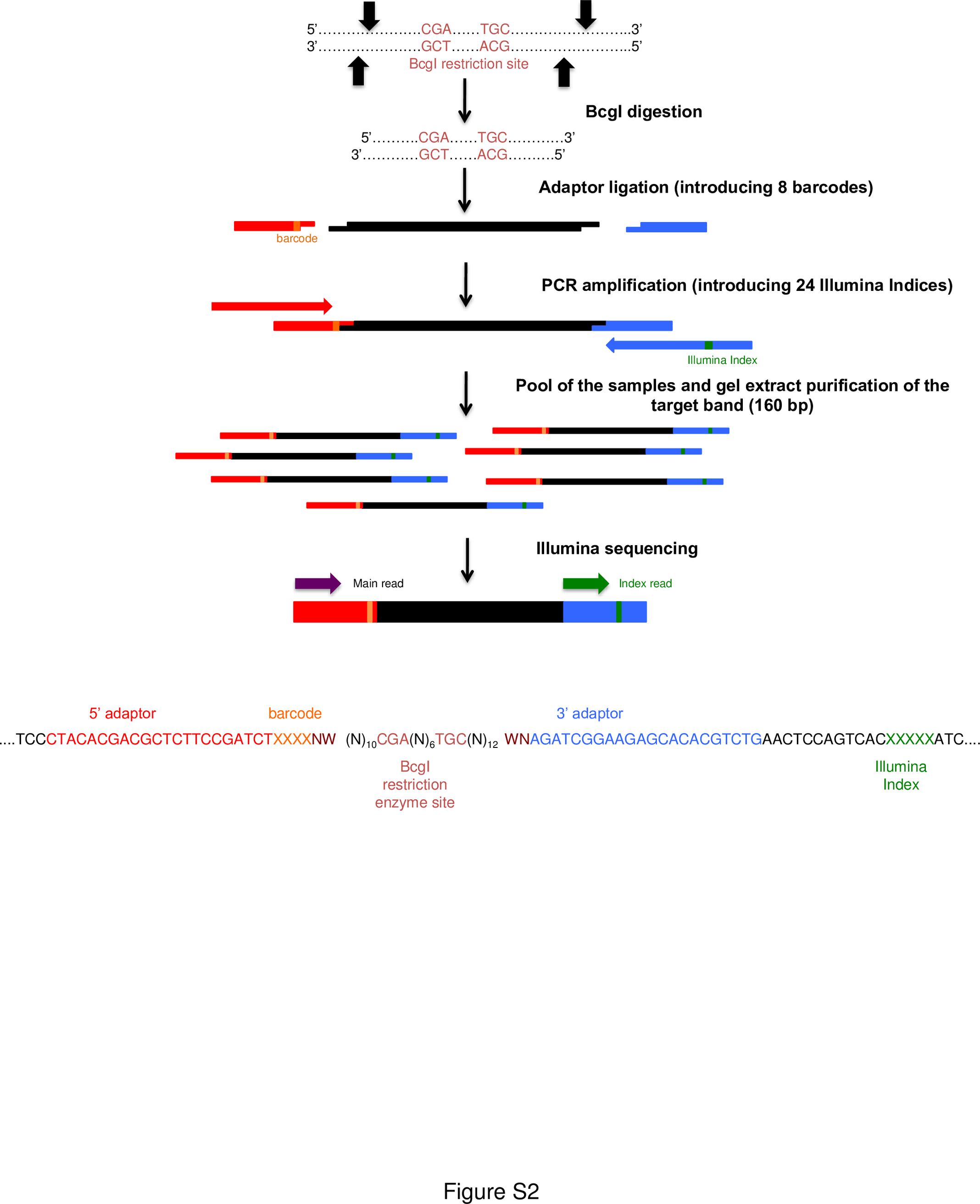

Supplement: Supplementary file 12 [file 807FileS1.zip › FigureS2.tiff]

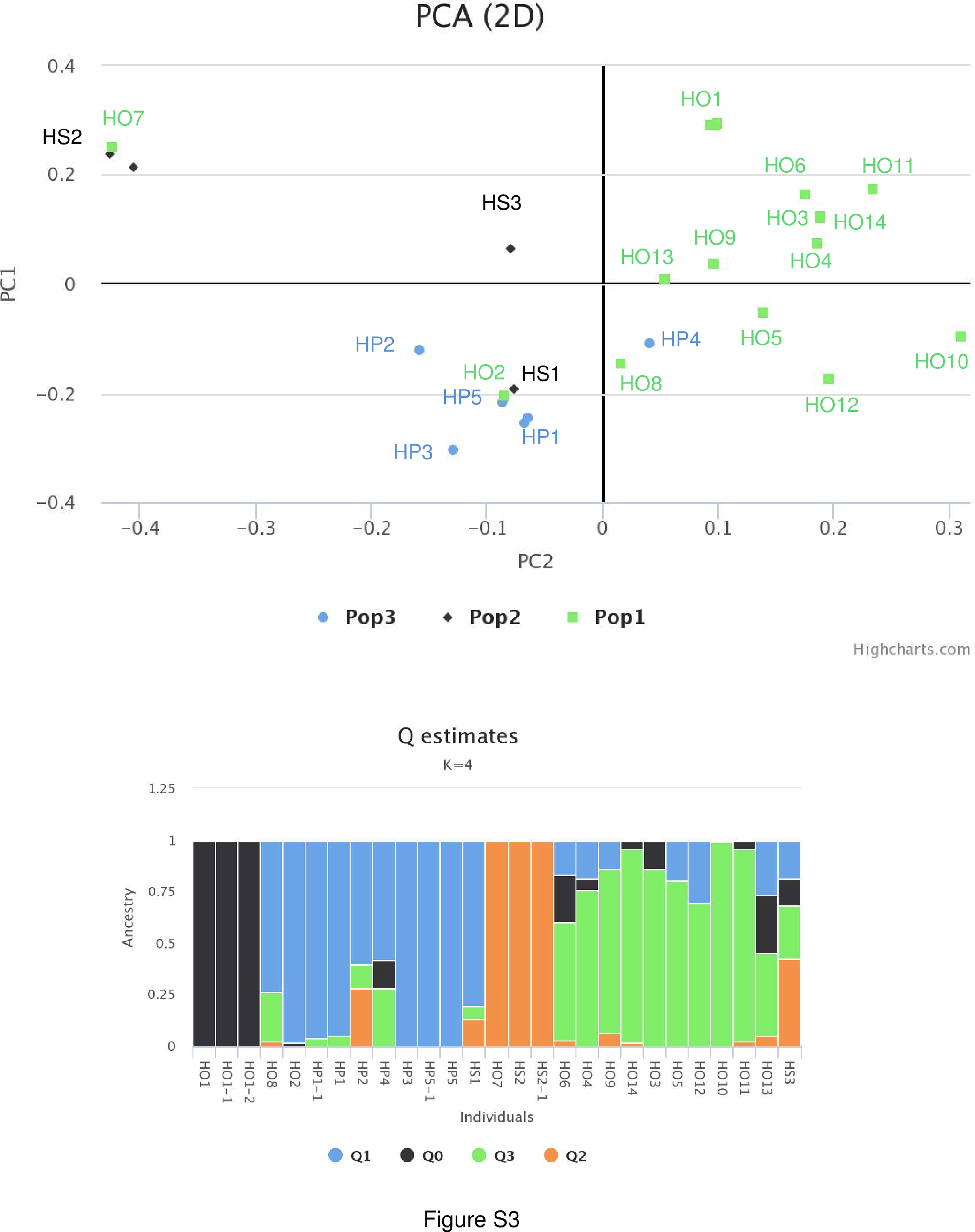

Supplement: Supplementary file 12 [file 807FileS1.zip › FigureS3.tiff]

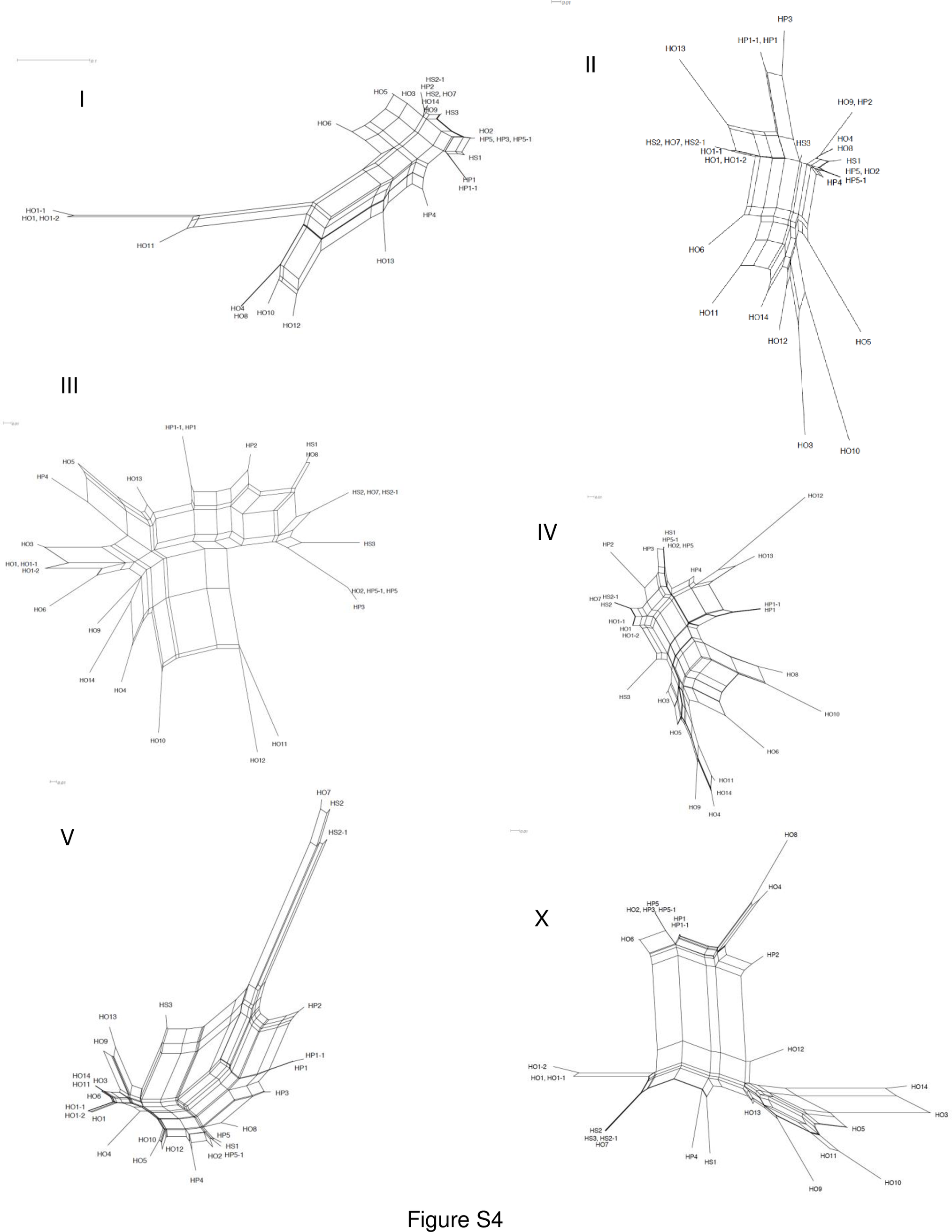

Supplement: Supplementary file 12 [file 807FileS1.zip › FigureS4.tiff]
